# Pharmacogenetics Guidelines: Overview and Comparison of the DPWG, CPIC, CPNDS, and RNPGx Guidelines

**DOI:** 10.3389/fphar.2020.595219

**Published:** 2021-01-25

**Authors:** Heshu Abdullah-Koolmees, Antonius M. van Keulen, Marga Nijenhuis, Vera H. M. Deneer

**Affiliations:** ^1^ Division of Laboratories, Pharmacy, and Biomedical Genetics, Department of Clinical Pharmacy, University Medical Center Utrecht, Utrecht, Netherlands; ^2^ Division of Pharmacoepidemiology and Clinical Pharmacology, Department of Pharmaceutical Sciences, Utrecht Institute for Pharmaceutical Sciences (UIPS), Utrecht University, Utrecht, Netherlands; ^3^ Royal Dutch Pharmacists Association (KNMP), Hague, Netherlands

**Keywords:** pharmacogenetics, guidelines, pharmacogenomics, DPWG, CPIC, CPNDS, RNPGx, recommendations

## Abstract

Many studies have shown that the efficacy and risk of side effects of drug treatment is influenced by genetic variants. Evidence based guidelines are essential for implementing pharmacogenetic knowledge in daily clinical practice to optimize pharmacotherapy of individual patients. A literature search was performed to select committees developing guidelines with recommendations being published in English. The Dutch Pharmacogenetics Working Group (DPWG), the Clinical Pharmacogenetics Implementation Consortium (CPIC), the Canadian Pharmacogenomics Network for Drug Safety (CPNDS), and the French National Network (Réseau) of Pharmacogenetics (RNPGx) were selected. Their guidelines were compared with regard to the methodology of development, translation of genotypes to predicted phenotypes, pharmacotherapeutic recommendations and recommendations on genotyping. A detailed overview of all recommendations for gene-drug combinations is given. The committees have similar methodologies of guideline development. However, the objectives differed at the start of their projects, which have led to unique profiles and strengths of their guidelines. DPWG and CPIC have a main focus on pharmacotherapeutic recommendations for a large number of drugs in combination with a patient’s genotype or predicted phenotype. DPWG, CPNDS and RNPGx also recommend on performing genetic testing in daily clinical practice, with RNPGx even describing specific clinical settings or medical conditions for which genotyping is recommended. Discordances exist, however committees also initiated harmonizing projects. The outcome of a consensus project was to rename “extensive metabolizer (EM)” to “normal metabolizer (NM)”. It was decided to translate a CYP2D6 genotype with one nonfunctional allele (activity score 1.0) into the predicted phenotype of intermediate metabolizer (IM). Differences in recommendations are the result of the methodologies used, such as assessment of dose adjustments of tricyclic antidepressants. In some cases, indication or dose specific recommendations are given for example for clopidogrel, codeine, irinotecan. The following drugs have recommendations on genetic testing with the highest level: abacavir (HLA), clopidogrel (CYP2C19), fluoropyrimidines (DPYD), thiopurines (TPMT), irinotecan (UGT1A1), codeine (CYP2D6), and cisplatin (TPMT). The guidelines cover many drugs and genes, genotypes, or predicted phenotypes. Because of this and their unique features, considering the totality of guidelines are of added value. In conclusion, many evidence based pharmacogenetics guidelines with clear recommendations are available for clinical decision making by healthcare professionals, patients and other stakeholders.

## Introduction

The effects of drugs in terms of the beneficial outcomes of drug treatment, development of side effects, and toxicity are influenced by genetic variants. Many studies have shown that the pharmacokinetics and effects of drugs differ among patients with specific genetic profiles. Sufficient evidence is available for a clinically relevant impact of patients’ genotype on the balance between the benefits and risk of a substantial number of drugs. Evidence-based guidelines with pharmacotherapeutic recommendations for combinations of specific drugs and genotypes or predicted phenotypes are essential for implementing acquired pharmacogenetics knowledge in daily clinical practice. The Dutch Pharmacogenetics Working Group (DPWG) and the Clinical Pharmacogenetics Implementation Consortium (CPIC) have been developing guidelines for more than a decade (Swen et al., 2008; Swen et al., 2011a; Caudle et al., 2017). Recommendations are preferably made available at the time of drug prescribing and dispensing for a patient with a genotype that requires an action, such as a dose reduction (Swen et al., 2008; Swen et al., 2011a; Deneer and van Schaik, 2013).

Additional sources of information regarding the pharmacogenetics of drugs are the Summary of Product Characteristics (SmPC) approved by the European Medicines Agency (EMA) and other agencies, as well as drug labels approved by the US Federal Drug Agency (FDA). The number of drugs with pharmacogenetics information in their SmPCs or labels has increased over the years because of regulatory guidelines and policy (TanKoi et al., 2018). In addition, the PharmGKB website is an open pharmacogenetics support tool with curated and graded evidence of combinations of drugs and genes (Barbarino et al., 2018).

Several genes encoding cytochrome P450 enzymes are highly polymorphic. This requires a translation of genotypes into predicted phenotypes, such as the metabolizer status of an intermediate metabolizer (IM). However, it has been recognized that translation methods differ among researchers, pharmacogenetic laboratories, and groups of professionals developing guidelines (Van Schaik et al., 2008; Deneer V.H.M., 2013).

Clinical decision making by healthcare professionals is primarily based on multidisciplinary pharmacogenetic guidelines, in daily clinical practice. Therefore, one should become familiar with the process of guideline development and differences between guidelines. In this article, we compare the guidelines with regard to the methodology of development, translation of genotypes to predicted phenotypes, pharmacotherapeutic recommendations and recommendations on genotyping.

## Methods of the Literature Search

A systematic literature search was performed on June 19, 2020 in the PubMed database to select committees, consortia or working groups that aim to develop pharmacogenetics guidelines and to select their published guidelines. The search was performed with “pharmacogenetics or pharmacogenomics and guidelines.” The labels’ languages (English), species (humans), and text availability (full text) were also added to the search criteria. The selection criterion was as follows: guidelines published in the English language developed by committees having the intention to recommend on several drugs irrespective of indication, disease, or medical specialty.

HA screened the results of the PubMed search for relevance by reading the title and abstract of the articles. VD also screened the included articles by reading the title and abstract. Additionally, if HA had doubts about any article, she read the article and discussed it with VD.

The pharmacogenetics guidelines by the DPWG, the CPIC, the Canadian Pharmacogenomics Network for Drug Safety (CPNDS), and the French National Network (Réseau) of Pharmacogenetics (RNPGx) were found. An overview and comparison of these guidelines were performed. Articles from and about these pharmacogenetics groups from the aforementioned PubMed search were used. Moreover, additional searches were performed on the websites of the DPWG (www.knmp.nl) and CPIC (https://cpicpgx.org/guidelines/), as well as that of PharmGKB (www.pharmgkb.org), a worldwide resource for pharmacogenomic information, to obtain an overview of the existing guidelines (Barbarino et al., 2018).

## Results of the Literature Search

Pharmacogenetics guidelines of the DPWG, CPIC, CPNDS and RNPGx were found through performing the PubMed search. Supplementary Table S1 presents an overview of the recommendations (Hicks et al., 2015; Hicks et al., 2016; Swen et al., 2011b; Hershfield et al., 2012; Martin et al., 2012; Madadi et al., 2013; Scott et al., 2013; Amstutz et al., 2014; Caudle et al., 2014b; Clancy et al., 2014; Crews et al., 2014; Muir et al., 2014; Ramsey et al., 2014; Relling et al., 2014; Birdwell et al., 2015; Gammal et al., 2015; Shaw et al., 2015; Aminkeng et al., 2016; Lee et al., 2016; Bell et al., 2017; Hicks et al., 2015; Hicks et al., 2016; Johnson et al., 2017; Lamoureux and Duflot, 2017; Moriyama et al., 2017; Quaranta et al., 2017; Quaranta and Thomas, 2017; Woillard et al., 2017; Amstutz et al., 2018; Goetz et al., 2018; Phillips et al., 2018; Maagdenberg et al., 2018; Brown et al., 2019; Desta et al., 2019; Drögemöller et al., 2019; Gonsalves et al., 2019; Relling et al., 2019; Theken et al., 2020;). The DPWG and CPIC guidelines were compared by Bank et al. (2018) based on the guidelines published until March 1, 2017. Therefore, in this article, we describe the changes that have been made since this comparison was published. Also the CPNDS and RNPGx are included in this comparison in terms of the process of literature assessment, the process of guideline development, and the content of their recommendations.

As of July 1, 2020, the DPWG had reviewed more than 100 gene-drug pairs. 60 gene-drug pairs are considered by the DPWG as gene-drug interaction requiring action such as adjustment of the dose or monitoring of adverse side effects. The remaining gene-drug pairs do not require additional action or monitoring due to pharmacogenetics. 18 gene-drug pairs are classified as gene-drug interaction for which no action is needed, and 29 gene-drug pairs considered as no gene-drug interaction and no actions needed to be made (The Dutch Pharmacogenomic Working Group, 2020a). The CPIC has reviewed more than 400 gene-drug pairs and has published recommendations on 106 gene-drug pairs with sufficient evidence for at least one prescribing action in 24 published guidelines, the CPNDS recommend on 13 gene-drug pairs, and the RNPGx on 8 gene-drug pairs. The DPWG publishes their recommendations on the website www.knmp.nl (search for pharmacogenetic recommendations) and also as of recently in the *European Journal of Human Genetics*. Previously an overview was given in two publications in *Clinical Pharmacology & Therapeutics* (Swen et al., 2008; Swen et al., 2011a). The CPIC publishes their guidelines on their website https://cpicpgx.org/guidelines/as well as in *Clinical Pharmacology & Therapeutics*. The CPNDS publishes their guidelines in several journals, while the guidelines of the RNPGx are published in *Thérapie*. All the guidelines can also be found on the website of PharmGKB: www.pharmgkb.org (Ross et al., 2010; Picard et al., 2017; CPIC, 2020; PharmGKB, 2020). Furthermore, each group has its own method of grouping genes and/or drugs or drug classes; for example, some guidelines include one gene and one drug, whereas others include two to three genes and one or more drugs (Amstutz et al., 2014; Picard et al., 2017; CPIC, 2020; The Dutch Pharmacogenomic Working Group, 2020a).

## Methodology for Guideline Development

The objectives of the DPWG and CPIC and their multidisciplinary processes of guideline development have been described and compared previously (Swen et al., 2008; Caudle et al., 2014a; Bank et al., 2018). Bank et al. compared the DPWG and CPIC based on the guidelines published until March 1, 2017. Bank et al. compared 30 gene-drug pairs, and since then, the DPWG has updated 24 of these gene-drug pairs, and the CPIC has updated 22 gene-drug pairs (Bank et al., 2018; CPIC, 2020; The Dutch Pharmacogenomic Working Group, 2020a).

The CPNDS, founded in 2004, aims to uncover the genetic and mechanistic basis of drug response phenotypes and improve the safety and efficacy of medications used in children and adults (Amstutz et al., 2014). The approaches of the DPWG, CPIC, and CPNDS are generally similar. An additional strength of the CPNDS is the involvement of patients and other stakeholders. The guideline development group is multidisciplinary, and patients and healthcare policy makers also participate. In short, guideline development consists of 1) a systematic literature search; 2) critical appraisal of the retrieved evidence; 3) development of clinical practice recommendations during a workshop meeting for guideline development group members; 4) internal review of draft guidelines by the guideline development group members; and 5) external review by content experts and members of the intended target audience (Amstutz et al., 2014).

The RNPGx has approximately 30 members across France as well as other French-speaking nations (i.e., Belgium and more recently Switzerland and Canada). The RNPGx board was elected by the members. Active RNPGx members are professionals with hospital activities in pharmacogenetics. The guidelines are elaborated by working groups of active members with specific expertise in the domain concerned, and are subsequently validated by the RNPGx board.

Methodologies for grading scientific evidence and the strength of the recommendation differ among the four groups of guideline developers (Amstutz et al., 2014; Picard et al., 2017; CPIC, 2020; The Dutch Pharmacogenomic Working Group, 2020a), see Supplementary Table S2. Furthermore, the DPWG, CPNDS, and RNPGx provide directions or recommendations regarding pharmacogenetic testing in daily clinical practice for a specific gene-drug combination.

### Dutch Pharmacogenetics Working Group

The methodology used by the DPWG for scoring the level of evidence and clinical impact is applied to all medication surveillance functionalities—such as drug interactions—of the drug database incorporated in drug prescribing and dispensing software in The Netherlands. Healthcare professionals have been familiar with this methodology for many years (Van Roon et al., 2005). A five-point-scale scoring system is used for the level of evidence (0–4) and a seven-point scale (AA^#^–F) is used for clinical relevance or impact, to which AA# has been added for statistically significant, positive clinical effects. For every scientific publication, both scores are assigned to each combination of genotype or predicted phenotype and a specific drug. Finally, the overall score of each combination is the highest level of evidence and the highest level of relevance assigned to any of the articles included in the assessment (Swen et al., 2008; Deneer and van Schaik, 2013). Adjustments of the starting dose are calculated based on the pharmacokinetic data in patients with a specific genotype or predicted phenotype. The method has previously been described in detail (Swen et al., 2008; Deneer and van Schaik, 2013). Recently, the DPWG has developed the clinical implementation score to advise and direct healthcare professionals on ordering pharmacogenetic testing before starting treatment with a specific drug or during treatment (Swen et al., 2018). The criteria for this score are as follows: 1) clinical effect, 2) level of evidence, 3) number needed to genotype, and 4) pharmacogenetics information in SmPCs. The total score is translated to three levels of pharmacogenetic testing in clinical practice, namely potentially beneficial, beneficial, and essential (Supplementary Table S3) (Swen et al., 2018).

### Clinical Pharmacogenetics Implementation Consortium

The CPIC guidelines are established according to a standardized format. The CPIC has a standard system for grading levels of evidence linking genotype to phenotype using three levels namely high, moderate, and weak. They use a system with three categories for their recommendations namely strong, moderate, and optional. For gene-drug combinations in the categories strong and moderate guidelines are being developed and published. “Strong recommendation” means “The evidence is high quality and the desirable effects clearly outweigh the undesirable effects” and a recommendation classified as “Moderate” means “There is a close or uncertain balance as to whether the evidence is high quality and the desirable clearly outweigh the undesirable effects” (CPIC, 2020).

### Canadian Pharmacogenomics Network for Drug Safety

Two grading schemes are used by the CPNDS. The quality of individual studies selected from the literature search are assessed. The grading scheme for the totality of evidence is based on the quality criteria of the Appraisal of Guidelines Research and Evaluation Enterprise (AGREE) and consists of four grades (+ to ++++) varying from the lowest grade +, meaning “Inconsistent or insufficient quantity/quality, discouraging” (further described as follows: “No conclusions can be drawn or conclusions are likely to change based on future studies, and current evidence is discouraging”) to the highest grade ++++, meaning “Consistent, generalizable” (further described as follows: “Strong general conclusions can be drawn that are unlikely to change based on further research”). The grading scheme for clinical practice recommendations (about the genotype guided treatment) has three levels (i.e., A: strong, B: moderate, and C: optional) varying from “Based on strong scientific evidence; benefits clearly outweigh risks (A)” to “Based mainly on expert opinion, for use with evidence development in a research context (C).” Furthermore, preferences of patients are taken into account when developing the recommendations. A strong recommendation (level A) is expected to be chosen by a majority of informed healthcare providers and patients. A recommendation in the category “Moderate” is expected to require individualized informed decision making by patients and healthcare providers (Ross et al., 2010; Amstutz et al., 2014; Tanoshima et al., 2019).

### French National Network (Réseau) of Pharmacogenetics

The focus and aim of the RNPGx differ from the others and concern recommending pharmacogenetic testing. First, the level of evidence for the functionality of a variant of a pharmacogene is considered and assigned to a three-level scale: demonstrated, probable, or potential functionality. Only variants with demonstrated or probable functionality are considered for a recommendation of testing. Depending on the total evidence the network has issued, three levels of recommendation for pharmacogenetic tests namely essential test, advisable test, or possibly helpful test. These vary from “Demonstrated impact on a major clinical phenotype i.e. efficacy or toxicity (essential test)” to “Probable impact that remains to be demonstrated having led to expert consensus in favor of testing (possibly helpful test)” (Picard et al., 2017).

### Terminology of Predicted Phenotypes

In daily clinical practice, the term “extensive metabolizer” (EM) does not appear particularly intuitive and is often mistaken by healthcare professionals for a genotype predicted phenotype with increased enzyme function. The outcome of a consensus project initiated by CPIC was to rename it “normal metabolized” (NM). Additional terms for predicted phenotypes for drug metabolizing enzymes are “poor metabolizer” (PM), “intermediate metabolizer” (IM), “rapid metabolizer” (RM), and “ultrarapid metabolizer” (UM) (Caudle et al., 2017). The DPWG has adopted “normal metabolizer” (NM) for CYP2D6, CYP2C19, CYP3A4, CYP1A2, CYP2B6, TPMT, and NUDT15 (The Dutch Pharmacogenomic Working Group, 2020b). The CPNDS also used “normal metabolizer” in its guideline on tamoxifen (Tanoshima et al., 2019). However, for CYP3A5 the DPWG decided to maintain the phenotype terminology, “CYP3A5 non-expressor,” for carriers of two nonfunctional alleles (CYP3A5*3/*3), “CYP3A5 heterozygous-expressor” and “CYP3A5 homozygous-expressor.” In European populations, the CYP3A5*3/*3 prevalence is around 80% (Shuker et al., 2016). Furthermore, regulatory guidelines require clinical studies in European populations. This implies that drug dosages in SmPCs, namely standard dosages, are suitable for patients assigned to the CPIC’s PM phenotype, and in some cases higher dosages might be required in patients assigned to the CPIC’s NM phenotype. It was decided that this might increase prescribing errors in a European context because many drugs are CYP3A5 substrates, and healthcare professionals might intuitively reduce dosages for patients with a PM metabolizer phenotype.

The DPWG did not introduce RM as a metabolizer status, applicable to the CYP2C19 *1/*17 genotype, instead of NM. This would have no impact on the current guidelines, and the recommendations would remain unchanged. The guideline on antidepressants by the RNPGx shows that “slow metabolizer” is used as a predicted phenotype term instead of “poor metabolizer” for CYP2D6 and CYP2C19 (Quaranta et al., 2017).

For dihydropyrimidine dehydrogenase (DPD), encoded by the DPYD gene it is common to translate genetic test results into a gene activity score (AS), which is the sum of activity scores of the alleles a patient carries. In the last update, the DPWG restricted DPYD phenotype terms to AS and decided to no longer use metabolizer status. Others also use AS which is also translated into metabolizer groups. Furthermore, patients with two fully dysfunctional alleles and those with one fully dysfunctional allele and one associated with reduced activity of the DPD enzyme were assigned PM as a predicted phenotype. However, in the updated guideline, recommendations differ for these two groups of patients (Lunenburg et al., 2020).

### Genotype-To-Phenotype Translations

Translations from genotypes to predicted phenotypes differ among researchers. This has to be considered when assessing studies for guideline development. From the researchers’ perspective it is logical because they mostly study a specific drug. The effect of a genetic variant is drug dependent. It is quite common that a specific genotype of a CYP enzyme has a large effect on the pharmacokinetics of one drug while having no effect on others, although both drugs are substrates of the CYP enzyme. The metabolic ratio of the CYP2D6 probe drug dextromethorphan is increased by a factor of 3.0 in carriers of one nonfunctional CYP2D6 allele compared with subjects without such an allele (Sachse et al., 1997). The area under the plasma concentration vs. the time curve of trimipramine in subjects with one nonfunctional allele is increased by a factor of 2.5 compared with those without such an allele (Kirchheiner et al., 2003). However, the clearance of haloperidol in both groups is similar with the ratio of those with one nonfunctional allele to those without being 0.9 (Brockmöller et al., 2002).

Furthermore, it has been recognized that translation methods differ among the DPWG and CPIC, and more specifically in the genotype-to-phenotype translation of CYP2D6, DPYD, and CYP2C19.

At the start of guideline development in 2005, the DPWG reached a consensus with Dutch laboratory specialists in the field of pharmacogenetics. They decided to translate a CYP2D6 genotype with one nonfunctional allele with AS 1.0, for example, CYP2D6 *1/*4, into a predicted phenotype of IM. The result of the consensus meeting on this topic was shared with professionals involved in genetic testing in The Netherlands (Van Schaik et al., 2008). In their guideline on tamoxifen, the CPNDS also classified AS 1.0 for CYP2D6 as IM.

By contrast, the CPIC and RNPGx classified such a genotype as EM (Quaranta et al., 2017). Furthermore, members of the CPIC and DPWG established an international expert panel and recently initiated a consensus procedure using a modified Delphi method. The procedures and results have been described extensively (Caudle et al., 2020). Moreover, the decision was made to downgrade the value of the CYP2D6*10 allele to 0.25 for AS instead of 0.5. Furthermore, the consensus definitions are as follows: PM (AS 0), IM (AS 0.25–1.0), NM (AS 1.25–2.25), and UM (AS > 2.25).

Since the previous comparison by Bank et al. (2018), the guidelines of both the DPWG and CPIC have been updated regarding DPYD genetic variants, the translation of genotypes into activity scores of DPD, other phenotype categories such as PM and IM, and therapeutic recommendations for capecitabine among others. The DPWG translates genotypes into activity scores unless two variants associated with the reduced functionality of DPYD, such as c.1236G>A/c.2846A>T, are detected, or in case one variant is associated with fully dysfunctional DPD activity and one with reduced functionality, such as *2A/c.1236G>A. It was decided that DPD enzyme activity cannot be predicted correctly because compound heterozygosity is uncertain. In such cases, an additional phenotyping test is required to determine the DPD enzyme activity. Currently, measuring DPD enzyme activity in peripheral blood mononuclear cells is the most frequently used test in The Netherlands (Meulendijks et al., 2016; Lunenburg et al., 2020). The starting doses of capecitabine and 5-fluorouracil, for example, should be selected based on the totality of genotyping and phenotyping test results. The CPIC, on the other hand, assigns AS of 0 and 0.5 to PM and of 1 and 1.5 to IM (Amstutz et al. 2018). In the RNPGx guideline on the pharmacogenetics of anticancer drugs, only DPYD alleles and genotypes are mentioned without them being translated into predicted phenotypes. The DPWG also recommends performing additional phenotyping in patients with an AS of 0.

### Pharmacotherapeutic Recommendations

A detailed overview of the recommendations of the DPWG, CPIC, CPNDS, and RNPGx are included in Supplementary Table S1. Gene-drug combinations for which the DPWG concluded that no gene-drug interaction exists for example CYP1A2-clozapine, as well as for example SLC01B1-atorvastatin classified by CPIC as no gene-drug interaction, are not summarized. A selection of the discordances are discussed. Main differences from a clinical perspective are shown in Supplementary Table S4.

### Discordances Fluoropyrimidines and Irinotecan

The committees have published advises on several anti-cancer drugs. Pharmacotherapeutic recommendations of the DPWG and the CPIC guidelines on fluoropyrimidines are different in some aspects. The DPWG advises to initiate fluorouracil or capecitabine in patients with DPD AS of 1.0 or 1.5 at a starting dose of 50%, while the CPIC also recommends 50% followed by dose titration based on clinical judgement of the healthcare professional and TDM. One indicates that the drugs should be avoided or are contraindicated in patients with DPYD AS 0, while the DPWG adds that if this is not possible the residual DPD activity in mononuclear cells from peripheral blood should be determined, and the initial dose should be adjusted accordingly. The DPWG guideline also includes tegafur and cutaneous fluorouracil (CPIC, 2020; Lunenburg et al., 2020). The RNPGx refers to dose and pharmacotherapeutic recommendations of others (Quaranta and Thomas, 2017).

In addition, the DPWG recommends giving 70% of the irinotecan dose in patients with UGT1A1 PM predicted phenotype and increasing it if the patient can tolerate said dosage, which must be guided by the neutrophil count. The RNPGx recommends a 25–30% dose reduction in UGT1A1 *28/*28 patients for irinotecan 180–230 mg/m^2^ spaced by 2–3 weeks intervals. The RNPGx marks irinotecan at a higher dose (240 mg/m^2^ or higher spaced by 2–3 weeks intervals) as contraindicated for patients with the UGT1A1*28/*28 genotype (Quaranta and Thomas, 2017).

### Discordances Clopidogrel, Warfarin, Statins

Within the field of cardiovascular diseases, the pharmacogenetics of clopidogrel has been extensively studied. The DPWG, CPIC, and RNPGx have guidelines for the CYP2C19-clopidogrel pair (Scott et al., 2013; Lamoureux and Duflot, 2017; The Dutch Pharmacogenomic Working Group, 2020a). The DPWG recommends patients with the CYP2C19 IM phenotype being treated with an alternative to clopidogrel for the indications of percutaneous coronary intervention, stroke, or Transient Ischemic Attack (TIA), or that they double the daily dosage to 150 mg. They also recommend considering an alternative for CYP2C19 PM in case of percutaneous coronary intervention, stroke, or TIA. Moreover, the DPWG recommends no action in case of other indications for CYP2C19 IM patients, while one suggests measuring platelet function testing and selecting an alternative in case of high on treatment reactivity in PM patients. The CPIC and RNPGx recommend an alternative to clopidogrel in CYP2C19 IM and PM patients. The CIPC’s recommendation is only applicable for patients with an acute coronary syndrome undergoing percutaneous coronary intervention.

All the four committees developed guidelines for the CYP2C9-VKORC1-warfarin pair in terms of dose adjustments or for calculating the dose using an algorithm; see Supplementary Table S1 (Shaw et al., 2015; The Dutch Pharmacogenomic Working Group, 2016; Lamoureux and Duflot, 2017). The DPWG recommends using the EU-PACT algorithm (which includes maintenance dose and k = elimination rate constant per genotype) or dose reduction as shown in Supplementary Table S1. The CPIC and CPNDS recommend using www.WarfarinDosing.org to calculate the dosage by for example genetic information, co-medication, and target International Normalized Ratio (INR). The CPIC recommends also to use the Gage or the IWPC algorithms or both.

For patients with the SLCO1B1 *5/*5 and *1/*5 genotype, the RNPGx recommends avoiding high doses of statins and the concomitant use of OATP1B1 inhibitors and/or CYP3A4 inhibitors (such as amiodarone, verapamil, and diltiazem). They advise to lower the simvastatin dose to 20 mg per day or to select another statin (Lamoureux and Duflot, 2017; Picard et al., 2017). The DPWG recommends choosing an alternative for simvastatin in both SLCO1B1 521 CC and TC patients; see Supplementary Table S1 for the recommendations (The Dutch Pharmacogenomic Working Group, 2020a). In SLCO1B1 TC patients simvastatin doses exceeding 40 mg/day should be avoided, if selecting an alternative is not an option. On the other hand, the CPIC recommends reducing the normal dose for patients with intermediate or low function of the transporter or choosing an alternative (e.g. rosuvastatin or pravastatin) (Ramsey et al., 2014). Furthermore, the CPIC has no recommendations for atorvastatin while the DPWG recommends an alternative for atorvastatin in patients with the SLCO1B1 521 CC and TC genotype, and additional risk factors for statin induced myopathy as for simvastatin (Ramsey et al., 2014; The Dutch Pharmacogenomic Working Group, 2020a).

### Discordances Antidepressants

The dose recommendations from the CPIC and the RNPGx for CYP2D6-CYP2C19-tricyclic antidepressants (TCA) are mostly the same (Hicks et al., 2015; Hicks et al., 2016; Swen et al., 2011b; Quaranta and Thomas, 2017; The Dutch Pharmacogenomic Working Group, 2020a). They recommend avoiding high dose of tricyclic antidepressants in CYP2D6 UM and PM patients and CYP2C19 UM and PM patients, and also reducing the dose in patients with CYP2D6 IM (CPIC: −25% of the recommended dose, RNPGx: −50% of the recommended dose) in case depression is the indication; see Supplementary Table S1 (Hicks et al., 2015; Hicks et al., 2016; Quaranta and Thomas, 2017). The recommendations for TCAs are based on amitriptyline literature and extrapolated to other tertiary amines (clomipramine, doxepin, imipramine, and trimipramine) because of comparable pharmacokinetic properties

The DPWG recommends increasing the dose of tricyclic antidepressants in CYP2D6 UM patients, and has specific calculated dose recommendations per gene-drug pair in CYP2D6 IM and PM patients (The Dutch Pharmacogenomic Working Group, 2020a). Patients with CYP2C19 UM metabolize amitriptyline in a greater extent into nortriptyline. According to the DPWG no additional action is required for CYP2C19 UM patients starting amitriptyline (The Dutch Pharmacogenomic Working Group, 2020a). On the other hand, the CPIC recommends starting nortriptyline instead of amitriptyline (Hicks et al., 2015; Hicks et al., 2016; CPIC, 2020). The CPIC mentions that nortriptyline and desipramine are secondary amines TCAs and CYP2D6 is the main gene for their metabolism. Another example is the DPWG recommending to avoid clomipramine in patients with CYP2C19 UM for the indications obsessive compulsive disorder (OCD) and anxiety disorder. In case of depression, the DPWG also recommends avoiding clomipramine in CYP2D6 PM (The Dutch Pharmacogenomic Working Group, 2020a).

There is no reason for any adjustment in patients with the CYP2D6 PM predicted phenotype according to the DPWG (The Dutch Pharmacogenomic Working Group, 2020a). However, the CPIC recommends choosing an alternative for paroxetine or considering a 50% reduction and a 25–50% reduction for fluvoxamine (Hicks et al., 2015; CPIC, 2020). In addition, the DPWG recommends adjusting the maximum dose instead of adjusting the starting dose of (es)citalopram because of the risk of Torsade de Pointes at high plasma concentrations (The Dutch Pharmacogenomic Working Group, 2020a).

### Discordances Indication and Patient Population Specific Recommendations

Other differences between the recommendations is distinguishing between indications, as for example the DPWG has different recommendations for CYP2D6-codeine for cough and pain while the CPIC and RNPGx mention no indication (Picard et al., 2017; CPIC, 2020; The Dutch Pharmacogenomic Working Group, 2020a). Furthermore, the CPIC has specific recommendations for adults and pediatrics with respect to voriconazole and atomoxetine (Krebs and Milani, 2019; CPIC, 2020).

### Discordances Pharmacotherapeutic Recommendations Normal Metabolizers

Supplementary Table S1 shows that the CPIC, the RNPGx, and in some cases also the CPNDS give advices on the treatment of patients with a predicted phenotype of NM such as starting with standard dose, adjusting dose based on guidelines, and standard monitoring of treatment effect. In contrast to these committees, the DPWG does not recommend on drug treatment in NM patients. One exception is tacrolimus initiation in CYP3A5 homozygous expressors, named by CPIC as NM patients. Furthermore, the difference in the dose recommendations for CYP3A5-tacrolimus is as follows. The CPIC and the RNPGx recommend 1.5-2 times of the standard starting dose for both NM and IM as mentioned by the CPIC and *1/*1 and *1/*3 as mentioned by the RNPGx (Birdwell et al., 2015; Woillard et al., 2017). On the other hand, the DPWG recommends 1.5 times the normal dose for the CYP3A5 heterozygote expressor and 2.5 times for CYP3A5 homozygous expressor phenotype (The Dutch Pharmacogenomic Working Group, 2020a).

### Genetic Testing Recommendations

The DPWG, CPNDS, and RNPGx provide recommendations regarding pharmacogenetic testing in daily clinical practice for specific gene-drug combinations (Supplementary Table S1 and Supplementary Table S5). The grading schemes they use for these recommendations differ, but they all have three levels. The DPWG’s highest level is “essential” meaning “PGx testing for this gene-drug pair is essential for drug safety or efficacy". Genotyping must be performed before drug therapy has been initiated to guide drug and dose selection (The Dutch Pharmacogenomic Working Group, 2020a). The grading scheme of the CPNDS has “strong” as highest level (A), defined as “based on strong scientific evidence; benefits clearly outweigh risks” (Amstutz et al., 2014), while for the RNPGx the highest classification is “essential test” with the following description “demonstrated impact on a major clinical phenotype [response (efficacy, resistance)/toxicity] for therapeutic management; difficult or impossible to predict with a non-genetic approach; having led to expert agreement in favor of systematic testing” (Picard et al., 2017).

### Genetic Testing Anti-cancer Drugs

Among anti-cancer agents, both the DPWG and the RNPGx consider UGT1A1 genotyping essential before the start of the treatment with irinotecan and the RNPGx recommending genotyping more specifically for patients who will receive the intensified dose (>240 mg/m^2^). The CPNDS strongly recommends (level A) genetic testing before cisplatin initiation for the associated functional TPMT variants (*3A, *3B, and*3C) in all patients, and the functionally inactive TPMT *2 variant in children receiving cisplatin to prevent cisplatin-induced hearing loss (Lee et al., 2016). Also, the CPNDS recommends genotyping (level B – moderate) for RARG rs2229774, SLC28A3 rs7853758 and UGT1A6*4 rs17863783 variants in all children with cancer that will initiate doxorubicin or daunorubicin to prevent antracycline-induced cardiotoxicity.

Genotype testing is not recommended in adults, also not in children receiving other anthracyclines (level C – optional) (Aminkeng et al., 2016). Furthermore on the anticancer drugs, the CPNDS recommendation including testing for CYP2D6 before initiation of adjuvant tamoxifen treatment, is classified as level B – moderate (Drögemöller et al., 2019).

The RNPGx prefers phenotyping by measuring the physiological dihydro-uracil/uracil (UH2/U) metabolic ratio in serum, over DPYD genotyping (Quaranta and Thomas, 2017; Loriot et al., 2018). If phenotyping is not available, genotyping should be performed pretreatment with dose reductions if genetic variants are detected (ref). They refer to the DPWG and CPIC guidelines for dose reductions. A phenotyping test in addition to genotyping should be performed, according to the DPWG, if two genetic variants are detected. In these rare cases the selection of the starting dose is at the discretion of the treating physician and other health care professionals involved, taking both genotyping and phenotyping test results into account.

### Genetic Testing Clopidogrel and Warfarin

Also, the DPWG and the RNPGx consider CYP2C19 genotyping essential in patients who will be treated with clopidogrel because of a percutaneous coronary intervention. The DPWG also recommends testing in the case of stroke or TIA. Genotyping is advisable for CYP2C9-VKORC1-warfarin according to the RNPGx (Picard et al., 2017). The CPNDS recommends testing all warfarin-naïve patients for VKORC1 (−1639G>A), CYP2C9*2, and CYP2C9*3 before warfarin is started or within first 2 weeks of therapy (level B – moderate) (Shaw et al., 2015).

### Genetic Testing Antidepressants

Moreover, the recommendations by the DPWG differ per gene-drug pair for tricyclic antidepressants, as shown in Supplementary Table S1. The DPWG classifies genotyping for tricyclic antidepressants as potentially beneficial meaning genotyping can be considered per patient. The RNPGx classifies genotyping for CYP2C19-CYP2D6-tricyclic antidepressants advisable (Quaranta and Thomas, 2017).

### Genetic Testing Codeine

Guidelines regarding CYP2D6 genotype testing and codeine treatment are also available. This is considered essential according to the DPWG in case of planned doses of more than 20 mg every 6 h for adults and more than 10 mg every 6 h for children aged 12 years and older or in case of additional risk factors, such as comedication with CYP3A4 inhibitors and/or reduced kidney function. The CPNDS classifies genotyping as strong – level A in the treatment of young children and women with postpartum pain while breastfeeding meaning that they should be tested for CYP2D6 (Madadi et al., 2013). Furthermore, CPNDS assigned other levels of recommendations for children and adults having pain despite high doses of codeine (level B – moderate recommendation), and patients who receive codeine for the first time to rule out non-responders and the ones who are susceptible to adverse side effects of codeine (level C – optional recommendation).

### Genetic Testing Antiepileptics

As shown in Supplementary Table S1, the DPWG have published genotyping recommendations on the antiepileptics: phenytoine, lamotrigine, and oxcarbazepine in case of HLA-B*15:02. HLA-B*15:02 is common in patients of Asian descent, other than Japanese or Korean descent, and therefore is genotyping beneficial before (or directly after) starting the pharmacotherapy (The Dutch Pharmacogenomic Working Group, 2018a; The Dutch Pharmacogenomic Working Group, 2018b; The Dutch Pharmacogenomic Working Group, 2018c; The Dutch Pharmacogenomic Working Group, 2020a). Also the CPNDS recommends genotype testing for HLA-B*15:02 for all carbamazepine naïve patients prior to initiation of the pharmacotherapy (level A- strong) for those originating from populations where HLA-B*15:02 is common or its frequency is unknown or whose origin is unknown. The CPNDS classifies genotyping recommendation as optional (level C) in patients from populations where HLA-B*15:02 is rare. Testing for HLA-A*31:01 is classified as moderate (level B) by the CPNDS for all carbamazepine naïve patients before pharmacotherapy initiation (Amstutz et al., 2014).

### Genetic Testing Several Drugs

Furthermore, the DPWG recommends to genotype for HLA-B-abacavir, TPMT and NUDT15-azathioprine/mercaptopurine/thioguanine, before initiation of the pharmacotherapy (classified as essential) (The Dutch Pharmacogenomic Working Group, 2020a). Genotyping is considered beneficial for VKORC1-acenocoumarol/phenprocoumon according to the DPWG. This means that genotyping the patient can be performed before (or directly after) pharmacotherapy therapy has been initiated (The Dutch Pharmacogenomic Working Group, 2020a). Genotyping is advisable for CYP3A5-tacrolimus according to the RNPGx (Picard et al., 2017).

## Discussion

Four committees develop pharmacogenetics guidelines regarding drugs in different drug classes, irrespective of indication, disease, or medical specialty. Their processes of guideline development have been described. They have published methodologies, guidelines, recommendations, and advice in scientific journals in English. In general, their recommendations add to the effective and safe use of drugs. However, the objectives differed at the start of each project, which have led to unique profiles and strengths of their approaches and guidelines.

The DPWG was first to start its project in 2005, and from the beginning all the pharmacotherapeutic recommendations were included in the drug database incorporated in electronic healthcare systems in The Netherlands. This implies that an alert is generated in case a drug is prescribed or dispensed to a patient with a genotype that requires an action. The system presents a short text addressing the pharmacotherapeutic advice, a summary of the underlying mechanism of the interaction between the gene and drug, and clinical and/or pharmacokinetic effects (Deneer and van Schaik, 2013). The methodology is equal to other medication surveillance functionalities, such as on drug interactions, within the electronic systems. This has facilitated healthcare professionals becoming familiar with pharmacogenetics.

The guidelines translating genetic laboratory test results into actionable prescribing decisions are essential for implementation in daily clinical practice. The CPIC guidelines are sent out in the review process to over 400 CPIC members. The CPIC has published all guidelines in a peer-reviewed scientific journal, and they are therefore reviewed by external experts. Detailed, and evidence-based information regarding allele definition, functionality, frequencies, and phenotype translations from genotype data are given. They have been the initiators of several projects to harmonize and reach a consensus on topics in the field of pharmacogenetics (Caudle et al., 2017; Caudle et al., 2020).

The CPNDS was established to focus on severe adverse drug reactions, which is also reflected by the drugs and genes they have selected for their guideline development. Participants enroll patients with serious adverse drug reaction into the CPNDS program. They aim to identify novel predictive genomic markers of severe adverse drug reactions and to provide clinical genetic information to the patient and healthcare professional (Ross et al., 2010). A broad panel of stakeholders, including patients and healthcare policy makers, are involved in guideline development.

The RNPGx has an interest in pharmacogenetic testing in daily clinical practice. They indicate which genetic variants should be included in a test offered in daily clinical practice to improve pharmacotherapy. The RNPGx also states whether genotype testing is recommended in daily clinical practice (Picard et al., 2017). They specifically recommend clinical indications and circumstances for genotyping either before the start of treatment or in case of problems when a patient is being treated with a specific drug (Lamoureux and Duflot, 2017).

The DPWG has assessed many gene-drug pairs with a relatively large number of pairs that require no action. The members of the DPWG select gene-drug combinations for further analysis. Even if the literature search shows that the genotype of the gene involved, does not influence the effect of the drug or to an extent that is not clinically relevant, a complete assessment report is prepared and made available by including it in the drug database. Although no action, such as adjustments of the dose and additional monitoring, is necessary, it appeared that the information and the report are of value for healthcare professionals. For example, healthcare professionals generally considered the CYP1A2 genotype to be relevant for clozapine. However, the assessment report summarized the results of the studies and concluded that CYP1A2 genotypes and the effect of clozapine or its blood concentrations are not associated with for example any adverse drug event, considering the totality of available evidence (The Dutch Pharmacogenomic Working Group, 2020a).

As previously recognized by Bank et al., there are several other explanations for differences between the guidelines (Bank et al., 2018). Differences in methodology, in time points at which literature searches have been performed or guidelines have been updated, and differences in daily clinical practices between countries result in discordances in recommendations. The CPIC’s dose recommendations are usually based on consensus of experts. They use published literature for dose. So if it is not clear what dose should be used and there are alternative therapies, they will most likely recommend another drug. On the other hand, the DPWG calculates adjustments of doses per genotype or predicted phenotype, based on pharmacokinetic data such as area under the concentration time curves (AUC), steady state concentrations found in published studies (Swen et al., 2008; Deneer and van Schaik, 2013). This explains differences between the recommended starting doses of tricyclic antidepressants. Furthermore, they only recommend on adjusting the dose if genotypes have a clinically relevant effect on for example blood concentrations, considering the therapeutic range of the drug or in case an association between the genotype and response of the drug in terms of efficacy or adverse drug events, has been observed. This explains for example the differences between the recommended starting doses of paroxetine and fluvoxamine in patients with a predicted PM phenotype for CYP2D6. According to the CPIC, the starting dose should be reduced. The DPWG does not consider this necessary since the drugs have a large therapeutic window.

The extent to which therapeutic drug monitoring (TDM), meaning measuring concentrations of a drug in blood or plasma to optimize treatment, is applied in daily clinical practice, differs among countries. For example, the DPWG indicates that endoxifen plasma concentrations should be measured to select an appropriate dose of tamoxifen in CYP2D6 IM and PM patients in contrast to the CPIC and the CPNDS. Clinical practices are also reflected in the DPYD-fluoropyrimidines. The UH2/U metabolic ratio test seems the preferred test to assess DPD enzyme activity, in France. Currently, the DPD enzyme activity measurement in peripheral blood mononuclear cells is the best developed and implemented test in routine patient care in The Netherlands. The CPIC mentions TDM in their guideline (Amstutz et al. 2018; Swen et al., 2011b; Picard et al., 2017; CPIC, 2020; The Dutch Pharmacogenomic Working Group, 2020a).

The DPWG, CPNDS, and RNPGx advise on genotyping. In general, they recommend to order genotyping tests in routine patient care if the clinical benefit for patients is considered relevant for example by lowering the risk of developing side effects or the risk of inefficacy of drug treatment. The committees assess the available data from published studies and also include the SmPC or drug label approved by EMA and FDA respectively. The number of drugs with pharmacogenetic information in these documents has increased over the years (TanKoi et al., 2018). The information is either included based on studies available before or after marketing authorization being granted or as a result of safety issues post approval. For example, in the SmPC of abacavir in the section regarding therapeutic indications it is stated that before starting the treatment every patient should be tested for carrying the HLA-*5701 allele (European Medicines Agency, 2020b). Several years ago, the FDA issued a black box warning on clopidogrel and the warnings section of the SmPC was updated with respect to reduced effectiveness of clopidogrel in patients with a CYP2C19 predicted phenotype of PM (TanKoi et al., 2018). Recently, EMA published a direct health care professional communication (DHPC) about the fact that patients with partial or complete DPD deficiency have an increased risk of developing toxicity when receiving fluoropyrimidines. It has been added that genotyping or phenotyping is recommended before the start of treatment with fluoropyrimidines such as capecitabine (European Medicines Agency, 2020a). Siponimod is a CYP2C9 substrate with plasma concentrations being influenced by the patient’s CYP2C9 genotype. The posology of the SmPC indicates that CYP2C19 genotyping must be performed before starting the treatment. The CYP2C9 *3/*3 genotype is a contraindication and the maintenance dose for patients with the *2/*3 or *1/*3 genotype is lower as compared to all other genotypes (European Medicines Agency, 2020c).

Although it is evident that genotyping contributes to the appropriate and safe use of drugs, cost-effectiveness is often discussed in case of reimbursement issues. Several randomized controlled trials investigating a genotype guided strategy are available for some drugs in specific patient populations (Church, 2008; Claassens et al., 2019). It is not feasible to perform cost-effectiveness studies based on the results of randomized clinical trials or cost-effectiveness simulations for all drugs with a clinically relevant gene-drug interaction for all indications. However, the clinical implication score of the DPWG includes criteria that would determine the outcome of a cost-effectiveness study such as the clinical impact meaning the severity of the drug’s side effect or the clinical impact of diminished efficacy, the number needed to genotype. It is worthwhile mentioning that currently the PREPARE study is investigating the (cost)-effectiveness and clinical utility of applying the DPWG guideline after a panel of genes being tested (van der Wouden et al., 2017; Van Der Wouden et al., 2020).

In conclusion, evidence based recommendations for many gene-drug pairs are available for supporting clinical decision making by healthcare professionals, patients and other stakeholders. Although there are many similarities in the methodologies the committees use, their guidelines have unique profiles and strengths. Therefore, considering the totality of guidelines are of added value.

## Author Contributions

HA-K and VD discussed and decided upon the method for literature search. HA-K performed the Pubmed search and the search results were discussed by both of them. AK made a detailed overview of all the guidelines and recommendations. HA-K, VD, AK, and MN all had a major contribution in making the comparison between the guidelines and recommendations. HA-K and VD drafted the manuscript and all authors reviewed the manuscript.

## Funding

VD is chair of the DPWG, initiated by the Royal Dutch Pharmacists Association (KNMP). The DPWG receives funding from the KNMP. MN is employed by the KNMP. The KNMP, VD and HA-K receive funding from the European Community’s Horizon 2020 Programme under grant agreement No. 668353 (U-PGx).

## Conflict of Interest

The authors declare that the research was conducted in the absence of any commercial or financial relationships that could be construed as a potential conflict of interest.

The handling editor declared a past co-authorship with one of the authors VD.
