# Supplementary material for: Pharmacogenetics Guidelines: Overview and Comparison of the DPWG, CPIC, CPNDS, and RNPGx Guidelines
Source: Front Pharmacol. 2021 Jan 25;11:595219. doi: 10.3389/fphar.2020.595219 (PMC7868558; doi:10.3389/fphar.2020.595219)
Supplement: Supplementary file 1 [file table1.docx]

Table 1: An overview of the therapeutic recommendations, classification of evidence per gene-drug pair, and advice on genotyping according to the DPWG, CPIC, CPNDS, and RNPGx. ^#^

| **Gene** | **Drug** | **Gene-drug interaction** | **Phenotype** | **Action required?** | **Therapeutic recommendations, classification of evidence, and genotyping recommendation** | | **Ref.** |
| --- | --- | --- | --- | --- | --- | --- | --- |
| HLA-B | Abacavir | DPWG: Yes | HLA-B*5701 positive | Yes | Abacavir is contra-indicated for HLA-B*5701-positive patients. - Avoid abacavir. | 4E  Essential | (1) |
| VKORC1 | Acenocoumarol | DPWG: Yes | [rs9923231](https://www.pharmgkb.org/variant/PA166155091) AA | Yes | Monitoring by the ANTICOAGULATION CLINIC (National INR Monitoring Service):  - Use 50% of the standard initial dose. OTHERWISE: use 50% of the standard initial dose and monitor more frequent the INR .The initial dose and the maintenance dose can be calculated using an algorithm. However, for patients with two or more VKORC1 and/or CYP2C9 variations, the algorithm used in EU-PACT did not result in a significant reduction in the incidence of INRs above the target range when compared to an algorithm without genetic information. We are therefore unable to recommend the use of this algorithm at this time.  - A (non-validated) algorithm has been prescribed for children that should result in a better prediction of the maintenance dose for AA than the current guideline used by the Anticoagulation Clinic. | 4F  Beneficial | (1) |
|  |  |  | [rs9923231](https://www.pharmgkb.org/variant/PA166155091) AG | No | NO action is needed for this gene-drug interaction | 4C  Beneficial | (1) |
| HLA-B | Allopurinol | DPWG: Yes | HLA-B*5801 | Yes | Choose an alternative or to precede treatment with allopurinol tolerance induction. Because febuxostat is not associated with an increase of adverse events in HLA-B*5801 carriers, febuxostat is mentioned as a possible alternative. | 4F* | (2) |
| CYP2C19 | Amitriptyline | DPWG: Yes | UM  IM  PM | No | NO action is required for this gene-drug interaction. | 4A  4A  3A | (1) |
| CYP2D6 | Amitriptyline | DPWG: Yes | UM | Yes | 1. increase the dose to 1.4 times the standard dose, monitor the effect and side effects or the plasma concentrations and be alert to increased plasma concentrations of the cardiotoxic Z-10-hydroxy metabolites. Plasma concentrations of Z-hydroxy nortriptyine or Z-hydroxy amitriptyline higher than 40 ng/mL are considered toxic. 2. if a dose increase is not desirable due to the cardiotoxic hydroxy metabolite: avoid amitriptyline. Anti-depressants that are not metabolised by CYP2D6, or to a lesser extent, include citalopram and sertraline. | 3C  Potentially beneficial | (1) |
|  |  |  | IM | Yes | Use 75% of the standard dose and monitor the efficacy and side effects or the plasma concentrations of amitriptyline and nortriptyline to adjust the maintenance dose. | 3C  Potentially beneficial | (1) |
|  |  |  | PM | Yes | Use 70% of the standard dose and monitor the effect and side effects or the plasma concentrations of amitriptyline and nortriptyline to adjust the mainenance dose | 3A  Potentially beneficial | (1) |
| CYP2D6 | Aripiprazole | DPWG: Yes | UM | No | No action is needed for this gene-drug interaction. The genetic variation decreases the plasma concentration of the sum of aripiprazole and the active metabolite dehydroaripiprazole to a limited degree. There is no evidence that this increases the risk of reduced effectiveness. | 3AA  Potentially beneficial | (1,3) |
|  |  |  | IM | No | No action is needed for this gene-drug interaction. The genetic variation decreases the plasma concentration of the sum of aripiprazole and the active metabolite dehydroaripiprazole to a limited degree. There is no evidence that this increases the risk of reduced effectiveness. | 4C  Potentially beneficial | (1,3) |
|  |  |  | PM | Yes | Administer no more than 10 mg/day or 300 mg/month (67-75% of the standard maximum dose of aripiprazole). | 4B  Potentially beneficial | (1,3) |
| CYP2D6 | Atomoxetine | DPWG: Yes | UM | Yes | 1. Be extra alert to reduced efficacy of the treatment. 2. Advise the patient to contact their doctor in the event of inadequate effect. 3. An alternative can be selected as a precaution. Clonidine is not metabolised by CYP2D6. | 3AA* | (1,3) |
|  |  |  | IM | Yes | 1. In the event of side effects occurring and/or a response later than 9weeks: reduce the dose and check whether the effect is conserved. The plasma concentration of atomoxetine is a factor of 2-3 times higher for IM than for NM at the same dose. | 4B* | (1,3) |
|  |  |  | PM | Yes | 1. Start with the normal initial dose, bearing in mind that an increase in this dose probably will not be required. 2. Advise the patient to seek contact if side effects occur (such as decreased appetite, vomiting, abdominal pain, constipation, insomnia, early waking, drowsiness, irritability, pupil dilation and itching). 3. If the medicine is effective, but side effects occur: reduce the dose and check whether the effect is conserved. The plasma concentration of atomoxetine is a factor of 8-11 times higher for PM than for NM at the same dose. | 4B* | (1,3) |
| SLCO1B1 | Atorvastatin | DPWG: Yes | 512 CC | Yes | - Patient has ADDITIONAL SIGNIFICANT RISK FACTORS for statin-induced myopathy: 1. Choose an alternative. Rosuvastatin and pravastatin are influenced to a similar extent by SLCO1B1 polymorphisms but are not influenced by CYP3A4 inhibitors such as amiodarone, verapamil and diltiazem. Fluvastatin is not influenced by SLCO1B1 polymorphisms or CYP3A4 inhibitors. 2. If an alternative is not an option: advise the patient to contact their doctor in the event of muscle symptoms. - Patient has NO additional significant risk factors for statin-induced myopathy: 1. Advise the patient to contact their doctor in the event of muscle symptoms. | 4C* | (1) |
|  |  |  | 512 TC | Yes | - Patient has ADDITIONAL SIGNIFICANT RISK FACTORS for statin-induced myopathy: 1. Choose an alternative. Rosuvastatin and pravastatin are influenced to a similar extent by SLCO1B1 polymorphisms but are not influenced by CYP3A4 inhibitors such as amiodarone, verapamil and diltiazem. Fluvastatin is not influenced by SLCO1B1 polymorphisms or CYP3A4 inhibitors. 2. If an alternative is not an option: advise the patient to contact their doctor in the event of muscle symptoms. - Patient has NO additional significant risk factors for statin-induced myopathy: 1. Advise the patient to contact their doctor in the event of muscle symptoms. | 4C* | (1) |
| TPMT | Azathioprine and Mercaptopurine | DPWG: Yes | IM | Yes | -IMMUNOSUPPRESSION  . Start with 50% of the standard dose. Adjustment of the initial dose should be guided by toxicity (monitoring of blood counts) and effectiveness. Dose adjustment is not required for doses lower than 1.5 mg/kg per day for azathioprine or 0.75 mg/kg per day for mercaptopurine.  Note: more stringent dose reductions are necessary if the patient is also NUDT15 IM or NUDT15 PM.  -LEUKAEMIA: start with 50% of the standard mercaptopurine dose, or start with the standard dose and reduce to 50% if side effects necessitate a dose reduction It is not known whether dose reduction in advance results in the same efficacy as dose reduction based on toxicity. The initial dose should be adjusted based on toxicity (monitoring of the blood counts) and efficacy. Note: more stringent dose reductions are necessary if the patient is also NUDT15 IM or NUDT15 PM. | 4E* | (1) |
|  |  |  | PM | Yes | Choose an alternative or start with 10% of the standard dose. Any adjustment of the initial dose should be guided by toxicity (monitoring of blood counts) and effectiveness. If the dose is decreased: advise patients to seek medical attention when symptoms of myelosuppression (such as severe sore throat in combination with fever, regular nosebleeds and tendency to bruising) occur. | 4F* | (1) |
| NUDT15 | Azathioprine and Mercaptopurine | DPWG: Yes | IM | Yes | - IMMUNOSUPPRESSION:start with 50% of the standard dose. Adjustment of the initial dose should be performed based on toxicity (monitoring of the blood counts) and efficacy. Note: The percentage of 50% is based on the analogy with TPMT, for which the gene variants have a comparable effect on toxicity to those of NUDT15. A percentage of < 70% was calculated for NUDT15, but there were insufficient data available to calculate the exact percentage. Note: Dose adjustment based on the total of 6-TGN metabolites is not possible for these patients, as they develop toxicity within the therapeutic range that applies for patients without gene variants. - LEUKAEMIA: start at 50% of the standard mercaptopurine dose, or start with the standard dose and reduce to 50% if side effects necessitate a dose reduction. It is not known whether dose reduction in advance results in the same efficacy as dose reduction based on toxicity. Adjustment of the initial dose should be performed based on toxicity (monitoring of the blood counts) and efficacy. Monitoring should be performed at an increased frequency. Note: The percentage of 50% is based on the analogy with TPMT, for which the gene variants have a comparable effect on toxicity to those of NUDT15. A percentage of < 70% was calculated for NUDT15, but there were insufficient data available to calculate the exact percentage. Note: Dose adjustment based on the total of 6-TGN metabolites is not possible for these patients, as they develop toxicity within the therapeutic range that applies for patients without gene variants. Note: more stringent dose reductions are necessary if the patient is also TPMT IM or TPMT PM. | 4E  Essential | (1) |
|  |  |  | PM | Yes | - Avoid azathioprine and mercaptopurine. If it is not possible to avoid azathioprine and mercaptopurine: use 10% of the standard dose and advise patients to seek medical attention when symptoms of myelosuppression (such as severe sore throat in combination with fever, regular nosebleeds and tendency to bruising) occur. Any adjustment of the initial dose should be guided by toxicity (monitoring of blood counts) and efficacy. Note: The percentage of 10% is based on the analogy with TPMT, for which the gene variants have a comparable effect on toxicity to those of NUDT15. A percentage of < 20% was calculated for NUDT15 PM, but there were insufficient data available to calculate the exact percentage. Note: Dose adjustment based on the total of 6-TGN metabolites is not possible for these patients, as they develop toxicity within the therapeutic range that applies for patients without gene variants. | 4E  Essential | (1) |
| CYP2D6 | Brexpiprazole | DPWG: Yes | UM | No | NO action is required for this gene-drug interaction. The gene variation results in a reduction of the exposure to brexpiprazole, but there are no indications supporting a decrease in efficacy. | 0A  Potentially beneficial | (1) |
|  |  |  | IM | No | NO action is required for this gene-drug interaction. There are indications supporting an increase in the exposure to brexpiprazole, but no indications supporting an increase in side effects in patients with this gene variation. | 4AA  Potentially beneficial | (1) |
|  |  |  | PM | Yes | Use half of the standard dose. | 0A  Potentially beneficial | (1) |
| HLA | Carbamazepine | DPWG: Yes | HLA-B*1502  HLA-A*3101  HLA-B*1511 | Yes | Choose an alternative. | 4E*  4E*  4E* | (4) |
| DPYD | Capecitabine and Fluorouracil | DPWG: Yes | AS 0 | Yes | Capecitabine and Fluorouracil, systemic  1. Avoid fluorouracil (systemic) and capecitabine. Tegafur is not an alternative, as this is also metabolised by DPD. 2. If is not possible to avoid fluorouracil and capecitabine: determine the residual DPD activity in mononuclear cells from peripheral blood and adjust the initial dose accordingly. A patient with 0.5% of the normal DPD activity tolerated 0.8% of the standard dose (150 mg capecitabine every 5 days). A patient with undetectable DPD activity tolerated 0.43% of the standard dose (150 mg capecitabine every 5 days with every third dose skipped).  Fluorouracil, cutaneous  Avoid fluorouracil  NOTE: If a patient has two different genetic variations that lead to a non-functional DPD enzyme (e.g. *2A and *13), this recommendation only applies if the variations are on a different allele. If both variations are  on the same allele, this patient actually has a gene activity score 1, for which no increased risk of severe, potentially fatal toxicity has been found with cutaneous use. These two situations can only be distinguished by  determining the enzyme activity (phenotyping). This recommendation only applies if the patient has virtually no enzyme activity. | 4F  Essential (Capecitabine and Fluorouracil, systemic)  Potentially beneficial (Fluorouracil, cutaneous) | (1) |
|  |  |  | AS 1.0 | Yes | Start with 50% of the standard dose or avoid fluorouracil and capecitabine. Adjustment of the subsequent dose should be guided by toxicity and effectiveness. However, in one study involving 17 patients with gene activity 1, the average dose after titration was 57% of the standard dose. Tegafur is not an alternative, as this is also metabolised by DPD. | 4F  Essential  (Capecitabine and Fluorouracil, systemic)  Potentially beneficial (Fluorouracil, cutaneous) | (1) |
|  |  |  | AS 1.5 | Yes | Start with 50% of the standard dose or avoid fluorouracil and capecitabine. After starting treatment, the dose should be adjusted based on toxicity and effectiveness. In a study involving 17 patients with genotype *1/2846T*, the average dose after titration was 64% of the standard dose. For 51 patients with genotype *1/1236A*, the average dose after titration was 74% of the standard dose. Tegafur is not an alternative, as this is also metabolised by DPD. | 4F  Essential  (Capecitabine and Fluorouracil, systemic)  Potentially beneficial (Fluorouracil, cutaneous) | (1) |
|  |  |  | FENO | Yes | It is not possible to recommend a dose adjustment based on the genotype only.  - Determine the residual DPD activity in mononuclear cells from peripheral blood and adjust the initial dose based on phenotype and genotype, or avoid fluorouracil (systemic) and capecitabine. Tegafur is not an alternative, as this is also metabolized by DPD. | 4F  Essential  (Capecitabine and Fluorouracil, systemic)  Potentially beneficial (Fluorouracil, cutaneous) | (1) |
| CYP2C19 | Citalopram | DPWG: Yes | UM | No | NO action is needed for this gene-drug interaction. The gene variation increases conversion of citalopram to a weakly active metabolite. However, there is no significant effect on the plasma concentration of citalopram, the tolerance or the response. | 3AA  Potentially beneficial | (1) |
|  |  |  | IM | Yes | Do not exceed the following daily doses: 1. Adults up to 65 years: 30mg as tablets or 22mg as drops, 2. Adults 65 years or older: 15mg as tablets or 10mg as drops | 4A  Potentially beneficial | (1) |
|  |  |  | PM | Yes | Do not exceed the following daily doses (50% of the standard maximum dose): 1. adults up to 65 years: 20mg as tablets or 16mg as drops, 2. Adults 65 years or older: 10mg as tablets or 8mg as drops | 4A  Potentially beneficial | (1) |
| CYP2C19 | Clomipramine | DPWG: Yes | UM | Yes | Indication OBSESSIVE COMPULSIVE DISORDER or ANXIETY DISORDERS:  - Avoid clomipramine. Antidepressants that are not metabolized by CYP2C19 - or to a lesser extent - include, for example, fluoxetine, fluvoxamine and paroxetine. If it is not possible to avoid clomipramine: monitor the effect and side effects or the plasma concentrations of clomipramine and desmethylclomipramine. For obsessive compulsive disorder, the therapeutic plasma concentration of clomipramine is greater than 200 ng/mL in combination with a plasma concentration of desmethylclomipramine that is as low as possible.  For anxiety disorders, the therapeutic plasma concentration of clomipramine is approximately 100 ng/mL in combination with a plasma concentration of desmethylclomipramine lower than 200 ng/mL. A sum of the plasma concentrations of clomipramine and desmethylclomipramine exceeding 600 ng/mL is considered toxic. Add a low dose of fluvoxamine if necessary, to inhibit CYP2C19 and CYP1A2 and thereby inhibit the conversion of clomipramine to desmethylclomipramine. Indication DEPRESSION: no action required | 3A  Potentially beneficial (obsessive-compulsive or anxiety disorder) | (1) |
|  |  |  | IM | No | NO action is required for this gene-drug interaction. The gene variation does increase clomipramine plasma concentrations, but not clomipramine+desmethylclomipramine plasma concentrations, which determines side effects and efficacy in depression. The increase in the plasma concentration of clomipramine is favourable for the efficacy in anxiety and obsessive compulsive disorder. | 4A  Potentially beneficial (obsessive-compulsive or anxiety disorder) | (1) |
|  |  |  | PM | No | NO action is required for this gene-drug interaction. The gene variation increases the plasma concentration of clomipramine. However, there is insufficient evidence to substantiate an increase of the plasma concentration of clomipramine+desmethylclomipramine to such an extent that it increases the risk of side effects. The increase in the plasma concentration of clomipramine is favourable for the efficacy in anxiety and obsessive compulsive disorder. | 4A  Potentially beneficial (obsessive-compulsive or anxiety disorder) | (1) |
| CYP2D6 | Clomipramine | DPWG: Yes | UM | Yes | Use 1.5 times the standard dose and monitor the effect and side effects of the plasma concentrations of clomipramine and desmethylclomipramine to set the maintenance dose. For depression, the therapeutic range is 200-400 ng/mL for the sum of the plasma concentrations of clomipramine and desmethylclomipramine. For anxiety disorders, the therapeutic plasma concentration of clomipramine is approximately 100 ng/mL, in combination with a plasma concentration of desmethylclomipramine lower than 200 ng/mL. For obsessive compulsive disorder, the therapeutic plasma concentration of clomipramine is higher than 200 ng/mL, in combination with a plasma concentration of desmethylclomipramine that is as low as possible. If a dose increase is not wanted due to potential cardiotoxic hydroxy metabolites: avoid clomipramine. Antidepressants that are not metabolised by CYP2D6, or to a lesser extent, include citalopram and sertraline. | 3C  Potentially beneficial | (1) |
|  |  |  | IM | Yes | Use 70% of the standard dose and monitor the effect and side effects or the plasma concentrations of clomipramine and desmethylclomipramine. For depression, the therapeutic range is 200-400 ng/mL for the sum of the plasma concentrations of clomipramine and desmethylclomipramine. For anxiety disorders, the therapeutic plasma concentration of clomipramine is approximately 100 ng/mL, in combination with a plasma concentration of desmethylclomipramine lower than 200 ng/mLFor obsessive compulsive disorder, the therapeutic plasma concentration of clomipramine is higher than 200 ng/mL, in combination with a plasma concentration of desmethylclomipramine that is as low as possible. A sum of the plasma concentrations of clomipramine and desmethylclomipramine higher than 600 ng/mL is considered toxic. | 4C  Potentially beneficial | (1) |
|  |  |  | PM | Yes | Indication DEPRESSION:  - Use 50% of the standard dose and monitor the effect and side effects or the plasma concentrations of clomipramine and desmethylclomipramine in order to set the maintenance dose. The therapeutic range is 200-400 ng/mL for the sum of the plasma concentrations of clomipramine and desmethylclomipramine. Values higher than 600 ng/mL are considered toxic. Indication ANXIETY DISORDERS or OBSESSIVE COMPULSIVE DISORDER: if side effects occur: use 50% of the standard dose and monitor the effect and side effects or the plasma concentrations of clomipramine and desmethylclomipramine in order to set the maintenance dose. It is not known whether it is possible to reduce the dose to such an extent that the side effects disappear while the effectiveness is retained. Clomipramine and desmethylclomipramine both contribute to the side effects. Only clomipramine contributes to the effectiveness. For anxiety disorders, the therapeutic plasma concentration of clomipramine is approximately 100 ng/mL, in combination with a plasma concentration of desmethylclomipramine lower than 200 ng/mL. For obsessive compulsive disorder, the therapeutic plasma concentration of clomipramine is higher than 200 ng/mL, in combination with a plasma concentration of desmethylclomipramine that is as low as possible. A sum of the plasma concentrations of clomipramine and desmethylclomipramine higher than 600 ng/mL is considered toxic, whilst the therapeutic upper limit for depression is 400 ng/mL. If dose reduction does not have the desired effect: avoid clomipramine. Antidepressants that are not metabolised by CYP2D6, or to a lesser extent, include citalopram and sertraline. | 4C  Potentially beneficial | (1) |
| CYP2C19 | Clopidogrel | DPWG: Yes | UM | No | No action is required for this gene-drug interaction. The genetic variation results in increased conversion of clopidogrel to the active metabolite. However, this can result in both positive effects (reduction in the risk of serious cardiovascular and cerebrovascular events) and negative effects (increase in the risk of bleeding). | 4A  Essential | (1) |
|  |  |  | IM | Yes | PERCUTANEOUS CORONARY INTERVENTION, STROKE or TIA:  - Choose an alternative or double the dose to 150 mg/day (600 mg loading dose). Prasugrel, ticagrelor and acetylsalicylic acid/dipyridamole are not metabolised by CYP2C19 (or to a lesser extent). OTHER INDICATIONS: no action required | 4F  Essential | (1) |
|  |  |  | PM | Yes | PERCUTANEOUS CORONARY INTERVENTION, STROKE or TIA:  - Avoid clopidogrel. Prasugrel, ticagrelor and acetylsalicylic acid/dipyridamole are not metabolised by CYP2C19 (or to a lesser extent). OTHER INDICATIONS: determine the level of inhibition of platelet aggregation by clopidogrel. Consider an alternative in poor responders. Prasugrel and ticagrelor are not metabolised by CYP2C19 (or to a lesser extent). | 4F  Essential | (1) |
| CYP2D6 | Codeine | DPWG: Yes | UM | Yes | DOSES HIGHER THAN 20 mg every 6 hours for adults and 10 mg every 6 hours for children aged 12 years or older AND/OR ADDITIONAL RISK FACTORS, such as co-medication with CYP3A4 inhibitors and/or reduced kidney function: - Codeine is contra-indicated. if possible, select an alternative - For PAIN: do not select tramadol, as this is also metabolised by CYP2D6. Morphine is not metabolised by CYP2D6. Oxycodone is metabolised by CYP2D6 to a limited extent, but this does not result in differences in side effects in patients. - For COUGH: noscapine is not metabolised by CYP2D6.   DOSES LOWER THAN OR EQUAL TO 20 mg every 6 hours for adults and 10 mg every 6 hours for children aged 12 years or older AND NO ADDITIONAL RISK FACTORS, such as co-medication with CYP3A4 inhibitors and/or reduced kidney function: No action required. | 3F  Essential | (1,3) |
|  |  |  | IM | Yes | For COUGH: 1. No action required. For PAIN: It is not possible to offer adequately substantiated advice for dose adjustment based on the limited available literature for this phenotype. 1. Be alert to a reduced effectiveness. 2. In the case of inadequate effectiveness: 1. Try a dose increase., 2. If this does not work: choose an alternative. Do not select tramadol, as this is also metabolised by CYP2D6. Morphine is not metabolised by CYP2D6. Oxycodone is metabolised by CYP2D6 to a limited extent, but this does not result in differences in analgesia in patients. 3. If no alternative is selected: advise the patient to report inadequate analgesia. | 3A  Essential | (1,3) |
|  |  |  | PM | Yes | For COUGH: 1. No action required.   For PAIN: It is not possible to offer adequately substantiated advice for dose adjustment based on the limited available literature for this phenotype. 1. Choose an alternative. Do not select tramadol, as this is also metabolised by CYP2D6. Morphine is not metabolised by CYP2D6. Oxycodone is metabolised by CYP2D6 to a limited extent, but this does not result in differences in analgesia in patients. 2. If an alternative is not an option: advise the patient to report inadequate analgesia. | 4B  Essential | (1,3) |
| CYP2C19 | Doxepine | DPWG: Yes | PM  IM  UM | No | No action required. | 3A  3A  - | (1) |
| CYP2D6 | Doxepine | DPWG: Yes | UM | Yes | - Double the standard dose and monitor the effect and side effects or the plasma concentrations of doxepine and nordoxepine in order to set the maintenance dose. The therapeutic range is 100-250 ng/mL for the sum of doxepine and nordoxepine plasma concentrations. Values higher than 400 ng/mL are considered toxic. If a dose increase is not wanted due to the potentially cardiotoxic hydroxy metabolites: avoid doxepine. Antidepressants that are not metabolised by CYP2D6, or to a lesser extent, include citalopram and sertraline. | 3A  Potentially beneficial | (1) |
|  |  |  | IM | Yes | Use 80% of the standard dose and monitor the effect and side effects or the plasma concentrations of doxepine and nordoxepine in order to set the maintenance dose. The therapeutic range is 100-250 ng/mL for the sum of doxepine and nordoxepine plasma concentrations. Values higher than 400 ng/mL are considered toxic. | 3A  Potentially beneficial | (1) |
|  |  |  | PM | Yes | Use 40% of the standard dose and monitor the effect and side effects or the plasma concentrations of doxepine and nordoxepine in order to set the maintenance dose. The therapeutic range is 100-250 ng/mL for the sum of doxepine and nordoxepine plasma concentrations. Values higher than 400 ng/mL are considered toxic. | 3F  Potentially beneficial | (1) |
| CYP2B6 | Efavirenz | DPWG: Yes | IM | Yes | 1. Determine the efavirenz plasma concentration if side effects occur and reduce the dose if needed. In 14 IM adults, a dose reduction to 400 mg/day (2/3rd of the standard dose) was sufficient to achieve therapeutic plasma concentrations and to reduce or resolve side effects. The therapeutic range established for efavirenz is 1000-4000 ng/ml. | 4E* | (1) |
|  |  |  | PM | Yes | * Efavirenz in MONOpreparation, adults and children FROM 40 KG:  - Body mass index LESS THAN or EQUAL to 25:  1. The recommended initial dose is 400 mg/day and this dose should be titrated to plasma concentration if needed (further reduction to 200 mg/day or in rare cases an increase to 600 mg/day). The therapeutic range established for efavirenz is 1000-4000 ng/ml.  - Body mass index GREATER than 25: 1. The recommended initial dose is 600 mg/day and this dose should be titrated to plasma concentration if needed (reduction to 400 or 200 mg/day). The therapeutic range established for efavirenz is 1000-4000 ng/ml.  *Efavirenz in MONOpreparation, children LIGHTER THAN 40 KG:  1. Start with the standard dose and titrate this dose to plasma concentration if needed. In adults, therapeutic plasma concentrations were achieved at either 2/3rd of the standard dose (1/3rd of the patients) or 1/3rd of the standard dose (2/3rd of the patients).  In children younger than 3 years, therapeutic plasma concentrations were achieved at doses of approximately 10 mg/kg per day (as capsules) (100 mg/day for 7-14 kg and 150 mg/day for 14-17 kg; 50-75% of the standard dose). The therapeutic range established for efavirenz is 1000-4000 ng/ml.  *Efavirenz in COMBINATION preparation: 1. Initiate the combination preparation and titrate the efavirenz dose to plasma concentration if needed (reduction to 400 or 200 mg/day) The therapeutic range established for efavirenz is 1000-4000 ng/ml. | 4E* | (1) |
|  |  |  | *1/*5 | No | No action is needed. | 3AA | (1) |
|  |  |  | *5/*5 | No | No action is needed. | 3AA | (1) |
|  |  |  | *5/*6 or *5/*18 | Yes | There are no clinical or kinetic data on *5/*6 and *5/*18. However, because a difference between *1 and *5 has not been observed, the effect of these genotypes should be comparable to that of *1/*6 and *1/*18 (IM, genotype otherwise). For this reason, the same therapeutic recommendation is given for *5/*6 or *5/*18 as for IM (dose reduction guided by plasma concentration in case of adverse events). | - | (1) |
| CYP2D6 | Eliglustat | DPWG: Yes | UM | Yes | Eliglustat is contra-indicated. Choose an alternative if possible. | 0A* | (1) |
|  |  |  | IM | Yes | - Co-medication with BOTH a MODERATE to STRONG CYP2D6 INHIBITOR AND a MODERATE to STRONG CYP3A INHIBITOR: Eliglustat is contra-indicated. 1. Choose an alternative if possible. Strong CYP2D6 inhibitor: for example paroxetine, fluoxetine, quinidine, bupropione. Moderate CYP2D6 inhibitor: for example duloxetine, terbinafine, moclobemide, mirabegron, cinacalcet, dronedarone. Strong CYP3A inhibitor: for example ketoconazole, clarithromycin, itraconazole, cobicistat, indinavir, lopinavir, ritonavir, saquinavir, telaprevir, tipranavir, posaconazole, voriconazole, telithromycin, conivaptan, boceprevir. Moderate CYP3A inhibitor: for example erythromycin, ciprofloxacin, fluconazole, diltiazem, verapamil, aprepitant, atazanavir, darunavir, fosamprenavir, imatinib, cimetidine. - Co-medication with a STRONG CYP2D6 INHIBITOR (e.g. paroxetine, fluoxetine, quinidine, bupropione): 1. Use a dose of 84mg eliglustat 1x daily. - Co-medication with a MODERATE CYP2D6 INHIBITOR (for example duloxetine, terbinafine, moclobemide, mirabegron, cinacalcet, dronedarone): 1. Consider a dose of 84mg eliglustat 1x daily. 2. Be alert to side effects. - Co-medication with a STRONG CYP3A INHIBITOR (for example ketoconazole, clarithromycin, itraconazole, cobicistat, indinavir, lopinavir, ritonavir, saquinavir, telaprevir, tipranavir, posaconazole, voriconazole, telithromycin, conivaptan, boceprevir): 1. Choose an alternative if possible. 2. If an alternative is not an option: consider a dose of 84 mg eliglustat 1x daily and be alert to side effects. - Co-medication with a MODERATE CYP3A INHIBITOR (for example erythromycin, ciprofloxacin, fluconazole, diltiazem, verapamil, aprepitant, atazanavir, darunavir, fosamprenavir, imatinib, cimetidine): 1. Choose an alternative. 2. If an alternative is not an option: consider a dose of 84mg eliglustat 1x daily and be alert to side effects. - Co-medication with a STRONG CYP3A INDUCER (for example rifampicin, carbamazepine, phenobarbital, phenytoin, rifabutine, hypericum): Eliglustat is not recommended. The plasma concentration may decrease so sharply that a therapeutic effect cannot be achieved. 1. Choose an alternative if possible. - NO co-medication with a moderate or strong CYP2D6 or CYP3A inhibitor or strong CYP3A inducer: 1. Use the standard dose of 84mg 2x daily. | 0AA* | (1) |
|  |  |  | PM | Yes | - Co-medication with a STRONG CYP3A INHIBITOR (for example ketoconazole, clarithromycin, itraconazole, cobicistat, indinavir, lopinavir, ritonavir, saquinavir, telaprevir, tipranavir, posaconazole, voriconazole, telithromycin, conivaptan, boceprevir): Eliglustat is contra-indicated. 1. Choose an alternative if possible. - Co-medication with a MODERATE CYP3A INHIBITOR (for example erythromycin, ciprofloxacin, fluconazole, diltiazem, verapamil, aprepitant, atazanavir, darunavir, fosamprenavir, imatinib, cimetidine): Eliglustat is not recommended. 1. Choose an alternative if possible. - Co-medication with a WEAK CYP3A INHIBITOR (for example amlopidine, cilostazole, fluvoxamine, goldenseal, isoniazide, ranitidine, ranolazine): 1. Choose an alternative for the weak CYP3A inhibitor if possible. 2. If an alternative is not an option: Use a dose of 84mg eliglustat 1x daily and be alert to side effects. - Co-medication with a STRONG CYP3A INDUCER (for example rifampicin, carbamazepine, phenobarbital, phenytoin, rifabutine, hypericum): Eliglustat is not recommended. The plasma concentration may decrease so sharply that a therapeutic effect cannot be achieved. 1. Choose an alternative if possible. - NO co-medication with a CYP3A inhibitor or strong CYP3A inducer: 1. Use a dose of 84mg 1x daily. | 0AA* | (1) |
| CYP2C19 | Escitalopram | DPWG: Yes | UM | Yes | Avoid escitalopram. Antidepressants that are not metabolized or that are metabolised to a lesser extent by CYP2C19 are, for example, paroxetine or fluvoxamine. | 4C  Potentially beneficial | (1) |
|  |  |  | IM | Yes | Do not exceed the following doses (75% of the standard maximum dose): adults < 65 years 15 mg/day, =65 years 7.5 mg/day | 4A  Potentially beneficial | (1) |
|  |  |  | PM | Yes | Do not exceed the following doses (50% of the standard maximum dose): adults < 65 years 10 mg/day, =65 years 5 mg/day | 4C  Potentially beneficial | (1) |
| CYP2D6 | Flecainide | DPWG: Yes | IM | Yes | 1. Indications other than diagnosis of Brugada syndrome: reduce the dose to 75% of the standard dose and record an ECG and monitor the plasma concentration.2.Provocation test for diagnosis of Brugada syndrome:No action required.At a dose of 2.0 mg/kg body weight to a maximum of 150 mg, the response is better for patients with alleles that result in reduced activity.All 5 patients with these alleles and 20% of the patients with two fully active alleles exhibited a response within 30 minutes. | 3A* | (1) |
|  |  |  | UM | No | There are no data about the pharmacokinetics and/or the effects of flecainide in UM. Monitor the plasma concentration as a precaution and record an ECG or select an alternative. Examples of anti-arrhythmic drugs that are not metabolized via CYP2D6 (or to a lesser extent) include sotalol, disopyramide, quinidine and amiodarone. | -- | (1) |
|  |  |  | PM | Yes | 1. reduce the dose to 50% of the standard dose and record an ECG and monitor the plasma concentration. | 4F* | (1) |
| HLA-B | Flucloxacillin | DPWG: Yes | HLA-B*5701 | Yes | 1. Regularly monitor the patient’s liver function 2. Choose an alternative if liver enzymes and/or bilirubin levels are elevated | 4Fv | (1) |
| CYP2D6 | Fluvoxamine | DPWG: No | PM  IM  UM | No | No action required. | 3AA  -  - | (1) |
| CYP2D6 | Haloperidol | DPWG: Yes | IM | No | NO action is required for this gene-drug interaction | 4A | (1) |
|  |  |  | PM | Yes | 1. Advise the prescriber to: 1. decrease the initial dose to 50% of the standard initial dose and adjust the dose according to the effect, 2. or prescribe an alternative. Anti-psychotics that are not metabolised via CYP2D6 - or to a much lesser extent - include, for example, flupentixol, fluphenazine, quetiapine, olanzapine or clozapine. | 4C* | (1) |
|  |  |  | UM | Yes | It is not possible to offer substantiated advice for dose adjustment due to the limited amount of available literature. 1. Advise the prescriber to: 1. be alert to possible reduced plasma concentrations of haloperidol and reduced haloperidol and increase the dose based on results of therapeutic drug monitoring, 2. or prescribe an alternative according to the current guidelines. Anti-psychotics that are not metabolised via CYP2D6 - or to a much lesser extent - include, for example, flupentixol, fluphenazine, quetiapine, olanzapine or clozapine. | 4C* | (1) |
| F5 | Estrogen containing contraceptives | DPWG: Yes | Factor V Leiden heterozygous | Yes | - If the patient has a FAMILY HISTORY WITH A LOT OF THROMBOSIS, or has had a PREVIOUS THROMBOSIS: 1. Advise the prescriber to avoid the use of contraceptives that contain estrogens and prescribe an on-hormone contraceptive-such as a copper IUD - as an alternative. One could also opt for a progestogen-only contraceptive method, such as the depot injection, an IUD with levonorgestrel or an implant with etonogestrel. - OTHER CASES: 1. Advise the patient to avoid additional risk factors for thrombosis (obesity, smoking, etc.). | 4D* | (1) |
|  |  |  | Factor V Leiden homozygous | Yes | - If the patient has a FAMILY HISTORY WITH A LOT OF THROMBOSIS, or has had a PREVIOUS THROMBOSIS: 1. Advise the prescriber to avoid the use of contraceptives that contain estrogens and prescribe a non-hormone contraceptive-such as a copper IUD - as an alternative. One could also opt for a progestogen-only contraceptive method, such as the depot injection, an IUD with levonorgestrel or an implant with etonogestrel. - OTHER CASES: 1. Advise the patient to avoid additional risk factors for thrombosis (obesity, smoking, etc.) | 3D* | (1) |
| CYP2C19 | Imipramine | DPWG: Yes | PM | Yes | Use 70% of the standard dose and monitor the effect and side effects or the imipramine and desipramine plasma concentrations to determine the maintenance dose, or, avoid imipramine. Antidepressants that are not or to a lesser extent metabolised by CYP2C19 include, for example, nortriptyline, fluvoxamine and mirtazapine | 4A  Potentially beneficial | (1) |
|  |  |  | UM | No | The genetic variation increases imipramine plasma concentrations, but not imipramine+desipramine plasma concentrations, which govern effectiveness and side effects. | 4A  Potentially beneficial | (1) |
|  |  |  | IM | No | The genetic variation increases imipramine plasma concentrations, but not imipramine+desipramine plasma concentrations, which govern effectiveness and side effects. | 4A  Potentially beneficial | (1) |
| CYP2D6 | Imipramine | DPWG: Yes | PM | Yes | Use 30% of the standard dose and monitor the effect and side effects or the plasma concentrations of imipramine and desipramine in order to set the maintenance dose. The therapeutic range is 150-300 ng/mL for the sum of the imipramine and desipramine plasma concentrations. Values exceeding 500 ng/mL are considered toxic. | 4C  Potentially beneficial | (1) |
|  |  |  | IM | Yes | Use 70% of the standard dose and monitor the effect and side effects or the plasma concentrations of imipramine and desipramine in order to set the maintenance dose. The therapeutic range is 150-300 ng/mL for the sum of the imipramine and desipramine plasma concentrations. Values exceeding 500 ng/mL are considered toxic | 4A  Potentially beneficial | (1) |
|  |  |  | UM | Yes | Use 1.7 times the standard dose and monitor the effect and side effects or the plasma concentrations of imipramine and desipramine in order to set the maintenance dose. If a dose increase is not wanted due to the potentially cardiotoxic hydroxy metabolites: avoid imipramine. Antidepressants that are not metabolised by CYP2D6 - or to a lesser extent - include, for example, citalopram and sertraline. | 4A  Potentially beneficial | (1) |
| UGT1A1 | Irinotecan | DPWG: Yes | IM | No | NO action is needed for this gene-drug interaction. | 4E  Essential | (1) |
|  |  |  | PM | Yes | Start with 70% of the standard dose If the patient tolerates this initial dose, the dose can be increased, guided by the neutrophil count. | 4E  Essential | (1) |
| HLA-B | Lamotrigine | DPWG: Yes | HLA-B*1502 | Yes | Lamotrigine can induce the life-threatening cutaneous adverse events SJS/TEN and DRESS.  HLA-B*1502 has not been detected in a sample of 1350 Dutch persons. The HLA-B*1502 frequency is high in Asians, except for Japanese and Koreans. The DPWG considers genotyping of patients of Asian descent other than Japanese or Korean descent before starting lamotrigine to be beneficial for drug safety.   - It is advised to genotype these patients before (or directly after) drug therapy has been initiated to guide drug selection. - Because life-threatening adverse events lamotrigine should be avoided if possible, even if both the incidence and the risk increase are low. | 4E  Beneficial (patients of Asian, not-Japanese and not-Korean, descent) | (5) |
| CYP2C19 | Lansoprazole | DPWG: Yes | PM | No | The higher plasma concentration of lansoprazole results in an increase in the therapeutic effectiveness, without an increase in the incidence of side effects. | 4C  Potentially beneficial | (1) |
|  |  |  | IM | No | The higher plasma concentration of lansoprazole results in an increase in the therapeutic effectiveness, without an increase in the incidence of side effects. | 4C  Potentially beneficial | (1) |
|  |  |  | UM | Yes | For Helicobacter pylori ERADICATION THERAPY: 1. Use a 4-fold higher dose. 2. Advise the patient to contact their doctor if symptoms of dyspepsia persist. OTHER INDICATIONS: 1. Be alert to reduced effectiveness. 2. If necessary, use a 4-fold higher dose. 3. Advise the patient to report persisting symptoms of dyspepsia. | -  Potentially beneficial | (1) |
| TPMT | Mercaptopurine | DPWG: Yes | IM | Yes | -IMMUNOSUPPRESSION Start with 50% of the standard dose. Adjustment of the initial dose should be guided by toxicity (monitoring of blood counts) and effectiveness. Dose adjustment is not required for doses lower than 1.5 mg/kg per day for azathioprine or 0.75 mg/kg per day for mercaptopurine. -LEUKAEMIA: start with 50% of the standard mercaptopurine dose, or start with the standard dose and reduce to 50% if side effects necessitate a dose reduction It is not known whether dose reduction in advance results in the same efficacy as dose reduction based on toxicity. The initial dose should be adjusted based on toxicity (monitoring of the blood counts) and efficacy. Note: more stringent dose reductions are necessary if the patient is also NUDT15 IM or NUDT15 PM. | 4E* | (1) |
|  |  |  | PM | Yes | Choose an alternative or start with 10% of the standard dose. Any adjustment of the initial dose should be guided by toxicity (monitoring of blood counts) and effectiveness. If the dose is decreased: advise patients to seek medical attention when symptoms of myelosuppression (such as severe sore throat in combination with fever, regular nosebleeds and tendency to bruising) occur. | 4F* | (1) |
| NUDT15 | Mercaptopurine | DPWG: Yes | IM | Yes | - IMMUNOSUPPRESSION:start with 50% of the standard dose. Adjustment of the initial dose should be performed based on toxicity (monitoring of the blood counts) and efficacy. Note: The percentage of 50% is based on the analogy with TPMT, for which the gene variants have a comparable effect on toxicity to those of NUDT15. A percentage of < 70% was calculated for NUDT15, but there were insufficient data available to calculate the exact percentage. Note: Dose adjustment based on the total of 6-TGN metabolites is not possible for these patients, as they develop toxicity within the therapeutic range that applies for patients without gene variants. - LEUKAEMIA: start at 50% of the standard mercaptopurine dose, or start with the standard dose and reduce to 50% if side effects necessitate a dose reduction. It is not known whether dose reduction in advance results in the same efficacy as dose reduction based on toxicity. Adjustment of the initial dose should be performed based on toxicity (monitoring of the blood counts) and efficacy. Monitoring should be performed at an increased frequency. Note: The percentage of 50% is based on the analogy with TPMT, for which the gene variants have a comparable effect on toxicity to those of NUDT15. A percentage of < 70% was calculated for NUDT15, but there were insufficient data available to calculate the exact percentage. Note: Dose adjustment based on the total of 6-TGN metabolites is not possible for these patients, as they develop toxicity within the therapeutic range that applies for patients without gene variants. Note: more stringent dose reductions are necessary if the patient is also TPMT IM or TPMT PM. | 4E* | (1) |
|  |  |  | PM | Yes | - avoid azathioprine and mercaptopurine. If it is not possible to avoid azathioprine and mercaptopurine: use 10% of the standard dose and advise patients to seek medical attention when symptoms of myelosuppression (such as severe sore throat in combination with fever, regular nosebleeds and tendency to bruising) occur. Any adjustment of the initial dose should be guided by toxicity (monitoring of blood counts) and efficacy. Note: The percentage of 10% is based on the analogy with TPMT, for which the gene variants have a comparable effect on toxicity to those of NUDT15. A percentage of < 20% was calculated for NUDT15 PM, but there were insufficient data available to calculate the exact percentage. Note: Dose adjustment based on the total of 6-TGN metabolites is not possible for these patients, as they develop toxicity within the therapeutic range that applies for patients without gene variants. | 4E* | (1) |
| CYP2D6 | Metoprolol | DPWG: Yes | IM | Yes | If a GRADUAL REDUCTION in HEART RATE is desired, or in the event of SYMPTOMATIC BRADYCARDIA: 1. increase the dose in smaller steps and/or prescribe no more than 50% of the standard dose. OTHER CASES: 1. no action required | 4A* | (1) |
|  |  |  | PM | Yes | If a GRADUAL REDUCTION in HEART RATE is desired, or in the event of SYMPTOMATIC BRADYCARDIA: 1. increase the dose in smaller steps and/or prescribe no more than 25% of the standard dose. OTHER CASES: 1. no action required | 4C* | (1) |
|  |  |  | UM | Yes | 1. use the maximum dose for the relevant indication as a target dose. 2. if the effectiveness is still insufficient: increase the dose based on effectiveness and side effects to 2.5 times the standard dose or select an alternative. Possible alternatives include: HEART FAILURE: bisoprolol or carvedilol. Bisoprolol: advantage: not metabolised by CYP2D6; disadvantage: elimination depends on the kidney function. Carvedilol: advantage: elimination does not depend on the kidney function; disadvantage: is metabolised (to a lesser extent than metoprolol) by CYP2D6. OTHER INDICATIONS: atenolol or bisoprolol. Neither is metabolised by CYP2D6. | 4D* | (1) |
| CYP2D6 | Nortriptyline | DPWG: Yes | UM | Yes | Use 1.7 times the standard dose and monitor the effect and side effects or the plasma concentration of nortriptyline and be alert to an increase in the plasma concentration of the cardiotoxic metabolite Z-10-hydroxynortriptyline. Plasma concentrations of Z-hydroxynortriptyline exceeding 40 ng/mL are considered toxic. If a dose increase is not wanted due to the cardiotoxic hydroxy metabolite: avoid nortriptyline. Antidepressants that are not metabolised via CYP2D6 - or to a lesser extent - include, for example, citalopram and sertraline. | 3C  Potentially beneficial | (1,3) |
|  |  |  | IM | Yes | Use 60% of the standard dose and monitor the effect and side effects or the plasma concentration of nortriptyline in order to set the maintenance dose. The therapeutic range of nortriptyline is 50-150 ng/mL. Values exceeding 250 ng/mL are considered toxic. | 4C  Potentially beneficial | (1,3) |
|  |  |  | PM | Yes | Use 40% of the standard dose and monitor the effect and side effects or the plasma concentration of nortriptyline in order to set the maintenance dose. The therapeutic range of nortriptyline is 50-150 ng/mL. Values exceeding 250 ng/mL are considered toxic. | 3C  Potentially beneficial | (1,3) |
| CYP2C19 | Omeprazole | DPWG: Yes | PM | No | The higher plasma concentration of omeprazole results in an increase in the therapeutic effectiveness, without an increase in the side effects. | 4AA  Potentially beneficial | (1) |
|  |  |  | IM | No | The higher plasma concentration of omeprazole results in an increase in the therapeutic effectiveness, without an increase in the side effects. | 4AA  Potentially beneficial | (1) |
|  |  |  | UM | Yes | For Helicobacter pylori ERADICATION THERAPY: 1. use a 3-fold higher dose. 2. advise the patient to contact their doctor if symptoms of dyspepsia persist. OTHER INDICATIONS: 1. be alert to reduced effectiveness. 2. if necessary, use a 3-fold higher dose. 3. advise the patient to report persisting symptoms of dyspepsia. | 3E  Potentially beneficial | (1) |
| HLA-B | Oxcarbazepine | DPWG: Yes | HLA-B*1502 | Yes | Oxcarbazepine can induce the severe and possibly life-threatening cutaneous adverse events SJS/TEN and DRESS. HLA-B*1502 has not been detected in a sample of 1350 Dutch persons. However, the HLA-B*1502 frequency is high in Asians, except for Japanese and Koreans. The DPWG considers genotyping of patients of Asian descent other than Japanese or Korean descent  before starting oxcarbazepine to be beneficial for drug safety. It is advised to genotype these patients before (or directly after) drug therapy has been initiated to guide drug selection.   - If an alternative is possible, choosing an alternative is recommended. If an alternative is not possible, it is recommended to advise the patient to report any rash immediately. - Carbamazepine is excluded as a possible alternative, because the incidence of carbamazepine-induced SJS/TEN in these patients is 10 times the incidence of oxcarbazepine-induced SJS/TEN | 4D  Beneficial (patients of Asian, not-Japanese and not-Korean, descent) | (6) |
| CYP2C19 | Pantoprazole | DPWG: Yes | PM | No | The higher plasma concentration of pantoprazole results in an increase in the therapeutic effectiveness, without an increase in the side effects. | 4AA  Potentially beneficial | (1) |
|  |  |  | IM | No | The higher plasma concentration of pantoprazole results in an increase in the therapeutic effectiveness, without an increase in the side effects | 4AA  Potentially beneficial | (1) |
|  |  |  | UM | Yes | For Helicobacter pylori ERADICATION THERAPY: 1. use a 5-fold higher dose. 2. advise the patient to contact their doctor if symptoms of dyspepsia persist. OTHER INDICATIONS: 1. be alert to reduced effectiveness. 2. if necessary, use a 5-fold higher dose. 3. advise the patient to report persisting symptoms of dyspepsia. | 4A  Potentially beneficial | (1) |
| CYP2D6 | Paroxetine | DPWG: Yes | UM | Yes | It is not possible to offer substantiated advice for dose adjustment based on the literature. Avoid paroxetine. Antidepressants that are not metabolized by CYP2D6, or to a lesser extent, include for example citalopram or sertraline. | 4C  Potentially beneficial | (1,3) |
|  |  |  | IM | No | NO action is needed for this gene-drug interaction. | 4A  Potentially beneficial | (1,3) |
|  |  |  | PM | No | NO action is needed for this gene-drug interaction. | 4A  Potentially beneficial | (1,3) |
| VKORC1 | Phenprocoumon | DPWG: Yes | 1639AA | Yes | 1. Monitoring by a ANTICOAGULATION CLINIC: recommend to use 50% of the standard initial dose. 2.NO monitoring by a anticoagulation clinic: recommend to use 50% of the standard initial dose, recommend more frequent monitoring of the INR. For patients younger than 75 years, the initial dose and the maintenance dose can be calculated using an algorithm as found in EUPACT: see  [Algorithms coumarins](https://www.knmp.nl/producten/gebruiksrecht-g-standaard/medicatiebewaking-g-standaard/background-information-pharmacogenetics)  for a calculation tool in the form of an Excel file. However, for patients aged 75 years and older, this algorithm increases the risk of an INR above the therapeutic range compared to an algorithm without gene variations. Therefore, use of this algorithm is not recommended for these patients. | 4D  Beneficial | (1) |
|  |  |  | 1639AG | No | NO action is needed for this gene-drug interaction | 4D | (1) |
| HLA-B | Phenytoin | DPWG: Yes | HLA-B*1502 | Yes | Phenytoin can induce the life-threatening cutaneous adverse events SJS/TEN and DRESS. hypersensitivity reactions generally develop between 2 weeks and 3 months  after the start of phenytoin. Because specific HLA proteins are involved in specific cellular immune reactions that cause specific hypersensitivity  reactions, HLA proteins can affect the risk of hypersensitivity reactions. HLA-B*1502 is present at a frequency of more than 1% only in persons of Southeast Asian ancestry (Han Chinese, Thai, Malaysians, Indians).   - If an alternative is possible, choose an alternative. If an alternative is not possible, it is recommended to advise the patient to report any rash immediately. - Carbamazepine is excluded as a possible alternative, because it increases the risk of severe cutaneous adverse events in these patients to a much higher extent than phenytoin.   Risk groups: CYP2C9 IM and PM. | 4E  Beneficial (patients of Asian, not-Japanese and not-Korean, descent) | (7) |
| CYP2C9 | Phenytoin | DPWG: Yes | CYP2C9*1/*2 | Yes | 1. The loading dose does not need to be adjusted. 2. For the other doses, use 75% of the standard dose and assess the dose based on effect and serum concentration after 7-10 days. 3. Advise the patient to get in touch if side effects (such as ataxia, nystagmus, slurred speech, sedation or rash) occur. | 4A* | (7) |
|  |  |  | CYP2C9*1/*3 | Yes | 1. The loading dose does not need to be adjusted. 2. For the other doses, use 75% of the standard dose and assess the dose based on effect and serum concentration after 7-10 days. 3. Advise the patient to get in touch if side effects (such as ataxia, nystagmus, slurred speech, sedation or, especially in Asian patients, rash) occur. | 4D* | (7) |
|  |  |  | CYP2C9*2/*2 | Yes | 1. The loading dose does not need to be adjusted. 2. For the other doses, use 50% of the standard dose and assess the dose based on effect and serum concentration after 7-10 days. 3. Advise the patient to get in touch if side effects (such as ataxia, nystagmus, slurred speech, sedation or rash) occur. | 4D* | (7) |
|  |  |  | CYP2C9*2/*3 | Yes | 1. The loading dose does not need to be adjusted. 2. For the other doses, use 50% of the standard dose and assess the dose based on effect and serum concentration after 7-10 days. 3. Advise the patient to get in touch if side effects (such as ataxia, nystagmus, slurred speech, sedation or rash) occur. | 4D* | (7) |
|  |  |  | CYP2C9*3/*3 | Yes | 1. The loading dose does not need to be adjusted.  2. For the other doses, use 40% of the standard dose and assess the dose based on effect and serum concentration after 7-10 days.  3. Advise the patient to get in touch if side effects (such as ataxia, nystagmus, slurred speech, sedation or, especially in Asian patients, rash) occur. | 4D* | (7) |
| CYP2D6 | Pimozide | DPWG: Yes | UM | No | NO action is required for this gene-drug interaction. | 3AA  Potentially beneficial | (1) |
|  |  |  | IM | Yes | Use no more than the following doses (80% of the standard maximum dose):  adults 16 mg/day  children 0.08 mg/kg per day to a maximum of 3 mg/day | 3A  Potentially beneficial | (1) |
|  |  |  | PM | Yes | Use no more than the following doses (50% of the standard maximum dose):  adults 10 mg/day  children 0.05 mg/kg per day to a maximum of 2 mg/day | 3A  Potentially beneficial | (1) |
| CYP2D6 | Propafenone | DPWG: Yes | UM | Yes | It is not possible to offer adequately substantiated recommendations for dose adjustment based on the literature.  1. Either monitor plasma concentrations, perform an ECG and be alert to reduced efficacy of the therapy.  2. Or choose an alternative. Antiarrhythmic drugs that are hardly if at all metabolized by CYP2D6 include, for example, sotalol, disopyramide, quinidine and amiodarone. | 3D* | (1) |
|  |  |  | IM | Yes | It is not possible to offer adequately substantiated recommendations for dose adjustment based on the literature.  1. Either guide the dose by therapeutic drug monitoring, perform an ECG and be alert to side effects  2. Or choose an alternative. Antiarrhythmic drugs that are hardly if at all metabolized by CYP2D6 include, for example, sotalol, disopyramide, quinidine and amiodarone. | 3A* | (1) |
|  |  |  | PM | Yes | Reduce the dose to 30% of the standard dose, perform an ECG and monitor plasma concentrations. | 4C* | (1) |
| CYP2D6 | Risperidone | DPWG: Yes | UM | Yes | Choose an alternative or titrate the dose according to the maximum dose for the active metabolite (paliperidone) (oral 12 mg/day for adults and children from 15 years of age weighing at least 51 kg and 6 mg/day for children from 15 years of age weighing less than 51 kg; intramuscular 75 mg per 2 weeks). | 4C* | (1,3) |
|  |  |  | IM | No | NO action is needed for this gene-drug interaction. | 4C | (1,3) |
|  |  |  | PM | Yes | Use 67% of the standard dose. If problematic side effects originating in the central nervous system occur despite this reduced dose, then reduce the dose further to 50% of the standard dose. | 4D* | (1) |
| CYP2C19 | Sertraline | DPWG: Yes | PM | Yes | Do not give doses exceeding 75 mg/day. Guide the dose by response and side effects and/or sertraline plasma concentration. | 4C  Potentially beneficial | (1) |
|  |  |  | IM | No | No action is needed for this gene-drug interaction. The gene variation has a minor effect on the sertraline plasma concentration. No effect on side effects was found. | 4A  Potentially beneficial | (1) |
|  |  |  | UM | No | No action is needed for this gene-drug interaction. The gene variation has a negligible effect on the plasma concentration of sertraline. Moreover, no significant effect on response and side effects has been found. | 4AA  Potentially beneficial | (1) |
| SLCO1B1 | Simvastatin | DPWG: Yes | 521 CC | Yes | 1. Choose an alternative. Consider any additional risk factors for statin-induced myopathy. Rosuvastatin and pravastatin are influenced to a lesser extent by SLCO1B1 polymorphisms. They are also not influenced by CYP3A4 inhibitors such as amiodarone, verapamil and diltiazem. Fluvastatin is not influenced by SLCO1B1 polymorphisms or CYP3A4 inhibitors. | 4D* | (1) |
|  |  |  | 521 TC | Yes | 1. Choose an alternative. Consider any additional risk factors for statin-induced myopathy. Rosuvastatin and pravastatin are influenced to a lesser extent by SLCO1B1 polymorphisms. They are also not influenced by CYP3A4 inhibitors such as amiodarone, verapamil and diltiazem. Fluvastatin is not influenced by SLCO1B1 polymorphisms or CYP3A4 inhibitors. 2. If an alternative is not an option: 1. Avoid simvastatin doses exceeding 40 mg/day 2. Advise the patient to contact their doctor in the event of muscle symptoms. | 4D* | (1) |
| CYP2C9 | Siponimod | DPWG: Yes | CYP2C9*1/*2 | No | NO action is required for this gene-drug interaction. | 3AA | (1) |
|  |  |  | CYP2C9*1/*3 | Yes | Use 50% of the normal maintenance dose. Reconsider the choice and the potential benefit of siponimod if the patient is also using a moderate CYP3A4 inducer, such as modafinil. For this genetic variation, a moderate CYP3A4 inducer results in a reduction in the exposure of siponimod by 49%, according to a pharmacokinetic model. | 3AA* | (1) |
|  |  |  | CYP2C9*2/*2 | No | NO action is required for this gene-drug interaction. | -- | (1) |
|  |  |  | CYP2C9*2/*3 | Yes | Use 50% of the normal maintenance dose. Reconsider the choice and the potential benefit of siponimod if the patient is also using a moderate CYP3A4 inducer, such as modafinil. For this genetic variation, a moderate CYP3A4 inducer results in a reduction in the exposure of siponimod by 49%, according to a pharmacokinetic model. | 3AA* | (1) |
|  |  |  | CYP2C9 *3/*3 | Yes | Avoid siponimod. | 3AA* | (1) |
| CYP3A5 | Tacrolimus | DPWG: Yes | CYP3A5 heterozygote expressor | Yes | In addition to the patient’s genotype, the metabolism of tacrolimus is also determined by the genotype of the transplanted liver.  LIVER is also of the genotype HETEROZYGOUS EXPRESSOR: Use 1.5 times the normal initial dose. Adjustment of the dose should then be based on therapeutic drug monitoring.  If LIVER has a DIFFERENT genotype: There is insufficient evidence in the literature to support a dose recommendation. | 4E* | (1) |
|  |  |  | CYP3A5 homozygous expressor | Yes | In addition to the patient’s genotype, the metabolism of tacrolimus is also determined by the genotype of the transplanted liver.  LIVER is also of the genotype HOMOZYGOUS EXPRESSOR: Use 2.5 times the normal initial dose. Adjustment of the dose should then be based on therapeutic drug monitoring.  LIVER has a DIFFERENT genotype: There is insufficient evidence in the literature to support a dose recommendation. | 4E* | (1) |
| CYP2D6 | Tamoxifen | DPWG: Yes | UM | No | NO action is needed for this gene-drug interaction. | 4A* | (1,3) |
|  |  |  | IM | Yes | 1. Select an alternative or measure the endoxifen concentration and increase the dose if necessary by a factor of 1.5-2. Aromatase inhibitors are a possible alternative for post-menopausal women.  2. If TAMOXIFEN is selected: avoid co-medication with CYP2D6 inhibitors such as paroxetine and fluoxetine | 4E* | (1,3) |
|  |  |  | PM | Yes | Select an alternative or increase the dose to 40 mg/day and monitor the endoxifen concentration. Studies have demonstrated that PM can achieve an adequate endoxifen concentration when the dose is increased to 40-60 mg/day. Aromatase inhibitors are a possible alternative for post-menopausal women. | 4F* | (1,3) |
| DPYD | Tegafur | DPWG: Yes | AS 0 | Yes | - avoid tegafur  Fluorouracil and capecitabine are not suitable alternatives, as these are also metabolised by DPD.  - If it is not possible to avoid tegafur: start with a very low dose and adjust the initial dose 0Ebased on toxicity and efficacy. A substantiated recommendation for dose reduction cannot be made based on the literature. | 0E  Essential | (1) |
|  |  |  | FENO | Yes | Avoid tegafur or start with a low dose and adjust the initial dose based on toxicity and efficacy. Fluorouracil and capecitabine are not alternatives, as these are also metabolised by DPD. It is not possible to offer substantiated advice for dose reduction based on the literature. | 0E  Essential | (1) |
|  |  |  | AS 1.0 | Yes | Avoid tegafur or start with a low dose and adjust the initial dose based on toxicity and efficacy. Fluorouracil and capecitabine are not alternatives, as these are also metabolised by DPD. It is not possible to offer substantiated advice for dose reduction based on the literature. | 2E  Essential | (1) |
|  |  |  | AS 1.5 | Yes | Avoid tegafur or start with a low dose and adjust the initial dose based on toxicity and efficacy. Fluorouracil and capecitabine are not alternatives, as these are also metabolised by DPD. It is not possible to offer substantiated advice for dose reduction based on the literature. | 2E  Essential | (1) |
| TPMT | Thioguanine | DPWG: Yes | IM | Yes | - IMMUNOSUPPRESSION: Start with 75% of the standard dose. Adjustment of the initial dose should be guided by toxicity (monitoring of blood counts) and efficacy. The frequency of monitoring should be increased.  - LEUKAEMIA: Start with 75% of the standard thioguanine dose, or start with the standard dose and reduce to 75% if side effects necessitate a dose reduction. It is not known whether dose reduction in advance results in the same efficacy as dose reduction based on toxicity. The initial dose should be adjusted based on toxicity (monitoring of the blood counts) and efficacy. Monitoring should be performed at an increased frequency.  Note: more stringent dose reductions are necessary if the patient is also NUDT15 IM. | 3E* | (1) |
|  |  |  | PM | Yes | 1. Choose an alternative or start with 6-7% of the standard dose. Any adjustment of the initial dose should be guided by toxicity (monitoring of blood counts) and effectiveness. The frequency of monitoring should be increased.  2. If the dose is decreased: advise patients to seek medical attention when symptoms of myelosuppression (such as severe sore throat in combination with fever, regular nosebleeds and tendency to bruising) develop. | 2F* | (1) |
| NUDT15 | Thioguanine | DPWG: Yes | IM | Yes | - IMMUNOSUPPRESSION: start with 75% of the standard dose. Adjustment of the initial dose should be performed based on toxicity (monitoring of the blood counts) and efficacy. Monitoring should be performed at an increased frequency.  NOTE: The percentage of 75% is based on the analogy with TPMT, for which the gene variants have a comparable effect on toxicity to those of NUDT15.  NOTE: Dose adjustment based on the total of 6-TGN metabolites is not possible for these patients, as they develop toxicity within the therapeutic range that applies for patients without gene variants.  - LEUKAEMIA: start with 75% of the standard thioguanine dose or start with the standard dose and reduce to 75% if side effects necessitate a dose reduction. It is not known whether dose reduction in advance results in the same efficacy as dose reduction based on toxicity. Adjustment of the initial dose should be performed based on toxicity (monitoring of the blood counts) and efficacy. Monitoring should be performed at an increased frequency.  NOTE: The percentage of 75% is based on the analogy with TPMT, for which the gene variants have a comparable effect on toxicity to those of NUDT15.  NOTE: Dose adjustment based on the total of 6-TGN metabolites is not possible for these patients, as they develop toxicity within the therapeutic range that applies for patients without gene variants.  Note: more stringent dose reductions are necessary if the patient is also TPMT IM. | 0E  Essential | (1) |
|  |  |  | PM | Yes | - avoid thioguanine  - if it is not possible to avoid thioguanine: use 10% of the standard dose and advise patients to seek medical attention when symptoms of myelosuppression (such as severe sore throat in combination with fever, regular nosebleeds and tendency to bruising) occur Any adjustment of the initial dose should be guided by toxicity (monitoring of blood counts) and efficacy. Monitoring should be performed at an increased frequency.  NOTE: The percentage of 10% is based on the analogy with azathioprine and mercaptopurine and the analogy with TPMT, for which the gene variants have a comparable effect on toxicity to those of NUDT15. For NUDT15 PM, a percentage of < 20% was calculated for azathioprine and mercaptopurine, but there were insufficient data available to calculate the exact percentage.  NOTE: Dose adjustment based on the total of 6-TGN metabolites is not possible for these patients, as they develop toxicity within the therapeutic range that applies for patients without gene variants. | 0E  Essential | (1) |
| CYP2D6 | Tramadol | DPWG: Yes | UM | Yes | As the total analgesic effect changes when the ratio between the mother compound and the active metabolite changes, the effect of a dose reduction cannot be predicted with certainty.  1. Select an alternative. Do not choose codeine, as it is contra-indicated for CYP2D6 UM. Morphine is not metabolised by CYP2D6. Oxycodone is metabolised by CYP2D6 to a limited extent, but this does not result in differences in side effects in patients.  2. If an alternative is not possible, use 40% of the standard dose. Advise the patient to report side effects (such as drowsiness, confusion, constipation, nausea and vomiting, respiratory depression or urine retention). | 3E  Potentially beneficial | (1,3) |
|  |  |  | IM | Yes | It is not possible to provide a recommendation for dose adjustment, because the total analgesic effect changes when the ratio between the mother compound and the active metabolite changes.  1. be alert to a reduced effectiveness  2. in the case of inadequate effectiveness:  a. try a dose increase  b. if this does not work: choose an alternative. Do not select codeine, as this is also metabolised by CYP2D6. Morphine is not metabolised by CYP2D6. Oxycodone is metabolised by CYP2D6 to a limited extent, but this does not result in differences in analgesia in patients.  3. if no alternative is selected: advise the patient to report inadequate analgesia | 4B  Potentially beneficial | (1,3) |
|  |  |  | PM | Yes | It is not possible to provide a recommendation for dose adjustment, because the total analgesic effect changes when the ratio between the mother compound and the active metabolite changes.  1. be alert to a reduced effectiveness  2. in the case of inadequate effectiveness:  a. try a dose increase.  b. if this does not work: choose an alternative. Do not select codeine, as this is also metabolised by CYP2D6. Morphine is not metabolised by CYP2D6. Oxycodone is metabolised by CYP2D6 to a limited extent, but this does not result in differences in analgesia in patients.  3. if no alternative is selected: advise the patient to report inadequate analgesia. | 4B  Potentially beneficial | (1,3) |
| CYP2D6 | Venlafaxine | DPWG: Yes | UM | Yes | 1. be alert to a possible decrease in the sum of the plasma concentrations of venlafaxine and the active metabolite O- desmethylvenlafaxine  2. if necessary, increase the dose to 150% of the standard dose  3. if dose adjustment does not result in efficacy without unacceptable side effects or if dose adjustment based on therapeutic drug monitoring is not possible, then venlafaxine should be avoided  Antidepressants that are not metabolised by CYP2D6 - or to a lesser extent - include, for example, duloxetine, mirtazapine, citalopram and sertraline. | 4A  Potentially beneficial | (1,3) |
|  |  |  | IM | Yes | It is not possible to offer adequately substantiated advice for dose reduction based on the literature.  - avoid venlafaxine. Antidepressants that are not metabolised by CYP2D6 - or to a lesser extent - include, for example, duloxetine, mirtazapine, citalopram and sertraline.  - if it is not possible to avoid venlafaxine and side effects occur:  1. reduce the dose  2. monitor the effect and side effects or check the plasma concentrations of venlafaxine and O-desmethylvenlafaxine.  It is not known whether it is possible to reduce the dose to such an extent that the side effects disappear, while the effectiveness is maintained. In general, it is assumed that the effectiveness is determined by the sum of the plasma concentrations of venlafaxine and O-desmethylvenlafaxine. However, the side effects do not appear to be related to this sum. | 4C  Potentially beneficial | (1,3) |
|  |  |  | PM | Yes | It is not possible to offer adequately substantiated advice for dose reduction based on the literature.  - avoid venlafaxine. Antidepressants that are not metabolised by CYP2D6 - or to a lesser extent - include, for example, duloxetine, mirtazapine, citalopram and sertraline.  - If it is not possible to avoid venlafaxine and side effects occur:  1. reduce the dose  2. monitor the effect and side effects or check the plasma concentrations of venlafaxine and O-desmethylvenlafaxine  It is not known whether it is possible to reduce the dose to such an extent that the side effects disappear, while the effectiveness is maintained. In general, it is assumed that the effectiveness is determined by the sum of the plasma concentrations of venlafaxine and O-desmethylvenlafaxine. However, the side effects do not appear to be related to this sum. Furthermore, a reduced effectiveness of venlafaxine has been observed in depression patients with this gene variation. | 4C  Potentially beneficial | (1,3) |
| CYP2C19 | Voriconazole | DPWG: Yes | PM | Yes | Use 50% of the standard dose and monitor the plasma concentration. | 4A* | (1) |
|  |  |  | IM | Yes | Monitor the plasma concentration. | 4A* | (1) |
|  |  |  | UM | Yes | Use an initial dose that is 1.5x higher and monitor the plasma concentration. | 4A* | (1) |
| CYP2C9 | Warfarin | DPWG: Yes | CYP2C9*1/*2 | No | NO action is required for this gene-drug interaction. | 4A | (8) |
|  |  |  | CYP2C9*1/*3 | Yes | 1. use 65% of the standard initial dose. The genotype-specific initial dose and maintenance dose can be calculated using an algorithm, as used in EU-PACT: see Algorithms coumarins. From day 6 on the standard algorithm without genotype information can be used to calculate the dose. | 4D* | (8) |
|  |  |  | CYP2C9*2/*2 | Yes | 1. use 65% of the standard initial dose. The genotype-specific initial dose and maintenance dose can be calculated using an algorithm, as used in EU-PACT: see Algorithms coumarins. From day 6 on the standard algorithm without genotype information can be used to calculate the dose. | 4A* | (8) |
|  |  |  | CYP2C9*2/*3 | Yes | 1. use 45% of the standard initial dose. The genotype-specific initial dose and maintenance dose can be calculated using an algorithm, as used in EU-PACT: see Algorithms coumarins. From day 6 on the standard algorithm without genotype information can be used to calculate the dose. | 4A* | (8) |
|  |  |  | CYP2C9*3/*3 | Yes | 1. use 20% of the standard initial dose. The genotype-specific initial dose and maintenance dose can be calculated using an algorithm, as used in EU-PACT: see Algorithms coumarins. From day 6 on the standard algorithm without genotype information can be used to calculate the dose. | 4C* |  |
| VKORC1 | Warfarin | DPWG: Yes | 1639 AA | Yes | 1. use 60% of the standard initial dose. The genotype-specific initial dose and maintenance dose can be calculated using an algorithm, as used in EU-PACT: see Algorithms coumarins. From day 6 on the standard algorithm without genotype information can be used to calculate the dose. | 4A* | (8) |
|  |  |  | 1639 AG | No | NO action is needed for this gene-drug interaction | 4A* | (8) |
| CYP2D6 | Zuclopenthixol | DPWG: Yes | UM | Yes | No data have been published from studies into the pharmacokinetics and effects of zuclopentixol for this phenotype.  As a precaution, the prescriber should advised to be alert to a decreased zuclopentixol plasma concentration and - if necessary - the dose should be increased on the basis of the clinical effect, or an alternative should be prescribed according to the current guidelines. Antipsychotics that are not metabolized via CYP2D6 - or to a lesser extent - include, for example, flupentixol, quetiapine, olanzapine and clozapine. | -- | (1,3) |
|  |  |  | IM | Yes | Advise the prescriber to start with 75% of the standard dose or to choose an alternative according to the current guidelines. Antipsychotics that are not metabolized via CYP2D6 - or to a lesser extent - include, for example, flupentixol, quetiapine, olanzapine and clozapine. | 4A* | (1,3) |
|  |  |  | PM | Yes | Advise the prescriber to start with 50% of the standard dose or to choose an alternative according to the current guidelines. Antipsychotics that are not metabolized via CYP2D6 - or to a lesser extent - include, for example, flupentixol, quetiapine, olanzapine and clozapine. | 4A* | (1,3) |
|  |  |  |  |  |  |  |  |
| CFTR | Ivacaftor | CPIC: Yes | G551D/ F508del, G551D/ G551D | No | Use ivacaftor according to the product label | Strong | (9) |
|  |  |  | F508del/F508del | Yes | Ivacaftor is not recommended. | Moderate | (9) |
|  |  |  | F508del/S549N | No | Use ivacaftor according to the product label | Moderate | (9) |
| CYP2B6 | Efavirenz | CPIC: Yes | UM | No | Initiate efavirenz with standard dosing (600 mg/day). | Strong | (10) |
|  |  |  | NM | No | Initiate efavirenz with standard dosing (600 mg/day). | Strong | (10) |
|  |  |  | IM | Yes | Consider initiating efavirenz with decreased dose of 400 mg/day. | Moderate | (10) |
|  |  |  | PM | Yes | Consider initiating efavirenz with decreased dose of 400 or 200 mg/ day. | Moderate | (10) |
| CYP2C19 | Clopidogrel | CPIC: Yes | UM | No | Label-recommended dosage and administration. | Strong | (11) |
|  |  |  | NM | No | Label-recommended dosage and administration. | Strong | (11) |
|  |  |  | IM | Yes | Alternative antiplatelet therapy (if no contraindication), e.g., prasugrel, ticagrelor. | Moderate | (11) |
|  |  |  | PM | Yes | Alternative antiplatelet therapy (if no contraindication), e.g., prasugrel, ticagrelor. | Strong | (11) |
| CYP2C19 | Voriconazole  (adults) | CPIC: Yes | UM | Yes | Choose an alternative agent that is not dependent on CYP2C19 metabolism as primary therapy in lieu of voriconazole. Such agents include isavuconazole, liposomal amphotericin B, and posaconazole. | Moderate | (13) |
|  |  |  | RM | Yes | Choose an alternative agent that is not dependent on CYP2C19 metabolism as primary therapy in lieu of voriconazole.Such agents include isavuconazole, liposomal amphotericin B, and posaconazole. | Moderate | (13) |
|  |  |  | NM | No | Initiate therapy with recommended standard of care dosing. | Strong | (13) |
|  |  |  | IM | No | Initiate therapy with recommended standard of care dosing. | Moderate | (13) |
|  |  |  | PM | Yes | Choose an alternative agent that is not dependent on CYP2C19 metabolism as primary therapy in lieu of voriconazole. Such agents include isavuconazole, liposomal amphotericin B, and posaconazole. In the event that voriconazole is considered to be the most appropriate agent, based on clinical advice, for a patient with poor metabolizer genotype, voriconazole should be administered at a preferably lower than standard dosage with careful therapeutic drug monitoring. | Moderate | (13) |
| CYP2C19 | Voriconazole  (pediatrics) | CPIC: Yes | URM | Yes | Choose an alternative agent that is not dependent on CYP2C19 metabolism as primary therapy in lieu of voriconazole. Such agents include liposomal amphotericin B, and posaconazole | Moderate | (13) |
|  |  |  | RM | Yes | Initiate therapy with recommended standard of care dosing. Use therapeutic drug monitoring to titrate dose to therapeutic trough concentrations | Moderate | (13) |
|  |  |  | NM | No | Initiate therapy with recommended standard of care dosing | Strong | (13) |
|  |  |  | IM | No | Initiate therapy with recommended standard of care dosing | Moderate | (13) |
|  |  |  | PM | Yes | Choose an alternative agent that is not dependent on CYP2C19 metabolism as primary therapy in lieu of voriconazole. Such agents include liposomal amphotericin B and posaconazole. In the event that voriconazole is considered to be the most appropriate agent, based on clinical advice, for a patient with poor metabolizer genotype, voriconazole should be administered at a preferably lower than standard dosage with careful therapeutic drug monitoring. | Moderate | (13) |
| CYP2C9 | Celecoxib, Flurbiprofen, Lornoxicam, and Ibuprofen | CPIC: Yes | NM | No | Initiate therapy with recommended starting dose. In accordance with the prescribing information, use the lowest effective dosage for shortest duration consistent with individual patient treatment goals. | Strong | (14) |
|  |  |  | IM (AS 1.5) | No | Initiate therapy with recommended starting dose. In accordance with the prescribing information, use the lowest effective dosage for shortest duration consistent with individual patient treatment goals. | Moderate | (14) |
|  |  |  | IM (AS 1.0) |  | Initiate therapy with lowest recommended starting dose. Titrate dose upward to clinical effect or maximum recommended dose with caution. In accordance with the prescribing information, use the lowest effective dosage for shortest duration consistent with individual patient treatment goals. Carefully monitor adverse events, such as blood pressure and kidney function during course of therapy. | Moderate | (14) |
|  |  |  | PM | Yes | Initiate therapy with 25–50% of the lowest recommended starting dose. Titrate dose upward to clinical effect or 25–50% of the maximum recommended dose with caution. In accordance with the prescribing information, use the lowest effective dosage for shortest duration consistent with individual patient treatment goals. Upward dose titration should not occur until after steady-state is reached (at least 8 days for celecoxib and 5 days for ibuprofen, flurbiprofen, and lornoxicam after first dose in PMs). Carefully monitor adverse events such as blood pressure and kidney function during course of therapy. Alternatively, consider an alternate therapy not metabolized by CYP2C9 or not significantly impacted by CYP2C9 genetic variants in vivo | Moderate | (14) |
| CYP2C9 | Meloxicam | CPIC: Yes | NM | No | Initiate therapy with recommended starting dose. In accordance with the prescribing information, use the lowest effective dosage for shortest duration consistent with individual patient treatment goals. | Strong | (14) |
|  |  |  | IM (AS 1.5) | No | Initiate therapy with recommended starting dose. In accordance with the meloxicam prescribing information, use the lowest effective dosage for shortest duration consistent with individual patient treatment goals. | Moderate | (14) |
|  |  |  | IM (AS 1.0) | Yes | Initiate therapy with 50% of the lowest recommended starting dose. Titrate dose upward to clinical effect or 50% of the maximum recommended dose with caution. In accordance with the meloxicam prescribing information, use the lowest effective dosage for shortest duration consistent with individual patient treatment goals. Upward dose titration should not occur until after steady-state is reached (at least 7 days). Carefully monitor adverse events, such as blood pressure and kidney function during course of therapy. Alternatively, consider alternative therapy. Choose an alternative therapy not metabolized by CYP2C9 or not significantly impacted by CYP2C9 genetic variants in vivo or choose an NSAID metabolized by CYP2C9 but with a shorter half-life | Moderate | (14) |
|  |  |  | PM | Yes | Choose an alternative therapy not metabolized by CYP2C9 or not significantly impacted by CYP2C9 genetic variants in vivo or choose an NSAID metabolized by CYP2C9 but with a shorter half-life | Moderate | (14) |
| CYP2C9 | Piroxicam and Tenoxicam | CPIC: Yes | NM | No | Initiate therapy with recommended starting dose. In accordance with the prescribing information, use the lowest effective dosage for shortest duration consistent with individual patient treatment goals. | Strong | (14) |
|  |  |  | IM (AS 1.5) | No | Initiate therapy with recommended starting dose. In accordance with the prescribing information, use the lowest effective dosage for shortest duration consistent with individual patient treatment goals. | Moderate | (14) |
|  |  |  | IM (AS 1.0) | Yes | Choose an alternative therapy not metabolized by CYP2C9 or not significantly impacted by CYP2C9 genetic variants in vivo or choose an NSAID metabolized by CYP2C9 but with a shorter half-life | Optional | (14) |
|  |  |  | PM | Yes | Choose an alternative therapy not metabolized by CYP2C9 or not significantly impacted by CYP2C9 genetic variants in vivo or choose an NSAID metabolized by CYP2C9 but with a shorter half-life | Optional | (14) |
| CYP2C9 /  HLA-B | Phenytoin | CPIC: Yes | CYP2C9 NM | Yes | HLA-B*1502 carrier: if patient is phenytoin naive, do not use phenytoin/ fosphenytoinc  HLA-B*1502 noncarrier; Initiate therapy with recommended maintenance dosed | Strong | (15) |
|  |  |  | CYP2C9 IM | Yes | HLA-B*1502 carrier: if patient is phenytoin naive, do not use phenytoin/ fosphenytoinc  HLA-B*1502 noncarrier: Consider 25% reduction of recommended starting maintenance dose. Subsequent maintenance doses should be adjusted according to therapeutic drug monitoring and response. | Moderate | (15) |
|  |  |  | CYP2C9 PM | Yes | HLA-B*1502 carrier: if patient is phenytoin naive, do not use phenytoin/ fosphenytoinc  HLA-B*1502 noncarrier: Consider 50% reduction of recommended starting maintenance dose. Subsequent maintenance doses should be adjusted according to therapeutic drug monitoring and response. | Strong | (15) |
| [*CYP2C9, VKORC1, CYP4F2*](https://cpicpgx.org/guidelines/guideline-for-warfarin-and-cyp2c9-and-vkorc1/) | Warfarin | CPIC: Yes | Non-African  VKORC1-1639G>A and CYP2C9*2 and *3 | Yes | Calculate dose based on validated pharmacogenetic algorithms.  (see [www.PharmGKB.org](http://www.PharmGKB.org)) | Strong | (16) |
|  |  |  | African  VKORC1-1639G>A and CYP2C9*2 and *3 | Yes | Calculate dose based on pharmacogenetic algorithms (see [www.PharmGKB.org](http://www.PharmGKB.org)) | Moderate | (16) |
|  |  |  | African carriers of CYP2C9*5. *6. *8 or *11 | Yes | Decrease calculated dose by 15-30%. (see [www.PharmGKB.org](http://www.PharmGKB.org)) | Moderate | (16) |
|  |  |  | CYP4F2*3 (rs2108622 T allele) | Yes | Increase the dose by 5-10%. (see [www.PharmGKB.org](http://www.PharmGKB.org)) | Optional | (16) |
| CYP2D6 | Atomoxetine  (Pediatrics) | CPIC: Yes | UM | No | Initiate with a dose of 0.5 mg/kg/day and increase to 1.2 mg/kg/day after 3 days. If no clinical response and in the absence of adverse events after 2 weeks, consider obtaining a peak plasma concentration (1–2 hours after dose administered). If < 200 ng/mL, consider a proportional increase in dose to approach 400 ng/mL | Moderate | (17) |
|  |  |  | NM (1.5-2) | No | Initiate with a dose of 0.5 mg/kg and increase to 1.2 mg/kg/day after 3 days. If no clinical response and in the absence of adverse events after 2 weeks, consider obtaining a peak plasma concentration (1–2 hours after dose administered). If < 200 ng/mL, consider a proportional increase in dose to approach 400 ng/mL. | Moderate | (17) |
|  |  |  | IM (AS 1.0) | Yes | Initiate with a dose of 0.5 mg/kg/day and if no clinical response and in the absence of adverse events after 2 weeks, consider obtaining a plasma concentration 2–4 hours after dosing. If response is inadequate and concentration is < 200 ng/mL, consider a proportional dose increase to achieve a concentration to approach 400 ng/mL. If unacceptable side effects are present at any time, consider a reduction in dose. | Moderate | (17) |
|  |  |  | IM (AS 0.5) | Yes | Initiate with a dose of 0.5 mg/kg/day and if no clinical response and in the absence of adverse events after 2 weeks, consider obtaining a plasma concentration 2–4 hours after dosing. If response is inadequate and concentration is < 200 ng/mL, consider a proportional dose increase to achieve a concentration to approach 400 ng/mL. If unacceptable side effects are present at any time, consider a reduction in dose. | Moderate | (17) |
|  |  |  | PM | Yes | Initiate with a dose of 0.5 mg/kg/day and if no clinical response and in the absence of adverse events after 2 weeks, consider obtaining a plasma concentration 4 hours after dosing. If response is inadequate and concentration is < 200 ng/mL, consider a proportional dose increase to achieve a concentration to approach 400 ng/mL. If unacceptable side effects are present at any time, consider a reduction in dose | Strong | (17) |
|  | Atomoxetine  (Adults) | CPIC: Yes | UM | No | Initiate with a dose of 40 mg/day and increase to 80 mg/ day after 3 days. If no clinical response and in the absence of adverse events after 2 weeks, consider increasing dose to 100 mg/day. If no clinical response observed after 2 weeks, consider obtaining a peak plasma concentration (1–2 hours after dose administered). If < 200 ng/mL, consider a proportional increase in dose to approach 400 ng/mL.Dosages > 100 mg/day may be needed to achieve target concentrations. | Moderate | (17) |
|  |  |  | NM | No | Initiate with a dose of 40 mg/day and increase to 80 mg/ day after 3 days. If no clinical response and in the absence of adverse events after 2 weeks, consider increasing dose to 100 mg/day. If no clinical response observed after 2 weeks, consider obtaining a peak plasma concentration (1–2 hours after dose administered). If < 200 ng/mL, consider a proportional increase in dose to approach 400 ng/mL. Dosages > 100 mg/day may be needed to achieve target concentrations | Moderate | (17) |
|  |  |  | IM (AS 1.0) | Yes | Initiate with a dose of 40 mg/day and if no clinical response and in the absence of adverse events after 2 weeks increase dose to 80 mg/day. If response is inadequate after 2 weeks consider obtaining a plasma concentration 2–4 hours after dosing. If concentration is < 200 ng/mL, consider a proportional dose increase to achieve a concentration to approach 400 ng/mL. If unacceptable side effects are present at any time, consider a reduction in dose. | Moderate | (17) |
|  |  |  | IM (AS 0.5) | Yes | Initiate with a dose of 40 mg/day and if no clinical response and in the absence of adverse events after 2 weeks increase dose to 80 mg/day. If response is inadequate after 2 weeks consider obtaining a plasma concentration 2–4 hours after dosing. If concentration is < 200 ng/mL, consider a proportional dose increase to achieve a concentration to approach 400 ng/mL If unacceptable side effects are present at any time, consider a reduction in dose. | Moderate | (17) |
|  |  |  | PM | Yes | Initiate with a dose of 40 mg/day and if no clinical response and in the absence of adverse events after 2 weeks increase dose to 80 mg/day. If response is inadequate after 2 weeks, consider obtaining a plasma concentration 2–4 hours after dosing. If concentration is < 200 ng/mL, consider a proportional dose increase to achieve a concentration to approach 400 ng/mL. If unacceptable side effects are present at any time, consider a reduction in dose. | Moderate | (17) |
| CYP2D6 | Codeine | CPIC: Yes | UM | Yes | Avoid codeine use due to potential for toxicity. | Strong | (18) |
|  |  |  | NM | No | Use label-recommended age or weight-specific dosing. | Strong | (18) |
|  |  |  | IM | No | Use label-recommended age or weight-specific dosing. If no response, consider alternative analgesics such as morphine or a nonopioid. | Strong | (18) |
|  |  |  | PM | Yes | Avoid codeine use due to lack of efficacy. | Strong | (18) |
| CYP2D6 | Ondansetron and  Tropisetron | CPIC: Yes | UM | Yes | Select alternative drug not predominantly metabolized by CYP2D6 (i.e., granisetron) | Strong | (19) |
|  |  |  | NM | No | Initiate therapy with recommended starting dose. | Strong | (19) |
|  |  |  | IM | No | Insufficient evidence demonstrating clinical impact based on CYP2D6 genotype. Initiate therapy with recommended starting dose. | No recommendation | (19) |
|  |  |  | PM | No | Insufficient evidence demonstrating clinical impact based on CYP2D6 genotype. Initiate therapy with recommended starting dose. | No recommendation | (19) |
| CYP2D6 | Tamoxifen | CPIC: Yes | UM | No | Avoid moderate and strong CYP2D6 inhibitors. Initiate therapy with recommended standard of care dosing (tamoxifen 20 mg/day). | Strong | (20) |
|  |  |  | NM | No | Avoid moderate and strong CYP2D6 inhibitors. Initiate therapy with recommended standard of care dosing (tamoxifen 20 mg/day). | Strong | (20) |
|  |  |  | IM (AS 1.0) | Yes | Consider hormonal therapy such as an aromatase inhibitor for postmenopausal women or aromatase inhibitor along with ovarian function suppression in premenopausal women, given that these approaches are superior to tamoxifen regardless of CYP2D6 genotype. If aromatase inhibitor use is contraindicated, consideration should be given to use a higher but FDA approved tamoxifen dose (40 mg/day). Avoid CYP2D6 strong to weak inhibitors. | Strong | (20) |
|  |  |  | IM (AS 0.5) | Yes | Consider hormonal therapy such as an aromatase inhibitor for postmenopausal women or aromatase inhibitor along with ovarian function suppression in premenopausal women, given that these approaches are superior to tamoxifen regardless of CYP2D6 genotype. If aromatase inhibitor use is contraindicated, consideration should be given to use a higher but FDA approved tamoxifen dose (40 mg/day). Avoid CYP2D6 strong to weak inhibitors. | Strong | (20) |
|  |  |  | PM | Yes | Recommend alternative hormonal therapy such as an aromatase inhibitor for postmenopausal women or aromatase inhibitor along with ovarian function suppression in premenopausal women given that these approaches are superior to tamoxifen regardless of CYP2D6 genotype and based on knowledge that CYP2D6 poor metabolizers switched from tamoxifen to anastrozole do not have an increased risk of recurrence. Note, higher dose tamoxifen (40 mg/day) increases but does not normalize endoxifen concentrations and can be considered if there are contraindications to aromatase inhibitor therapy. | Strong | (20) |
| CYP2D6 | Paroxetine | CPIC: Yes | UM | Yes | Select alternative drug not predominantly metabolized by CYP2D6 | Strong | (21) |
|  |  |  | NM | No | Initiate therapy with recommended starting dose. | Strong | (21) |
|  |  |  | IM | No | Initiate therapy with recommended starting dose. | Moderate | (21) |
|  |  |  | PM | Yes | Select alternative drug not predominantly metabolized by CYP2D6 or if paroxetine use warranted, consider a 50% reduction of recommended starting dose and titrate to response | Optional | (21) |
| CYP2D6 | Fluvoxamine | CPIC: Yes | UM | No | No recommendation due to lack of evidence | Optional | (21) |
|  |  |  | NM | No | Initiate therapy with recommended starting dose. | Strong | (21) |
|  |  |  | IM | No | Initiate therapy with recommended starting dose. | Strong | (21) |
|  |  |  | PM | Yes | Consider a 25–50% reduction of recommended starting dose and titrate to response or use an alternative drug not metabolized by CYP2D6. | Optional | (21) |
| CYP2C19 | Citalopram and Escitalopram | CPIC: Yes | UM | Yes | Consider an alternative drug not predominantly metabolized by CYP2C19 | Strong | (21) |
|  |  |  | NM | No | Initiate therapy with recommended starting dose. | Strong | (21) |
|  |  |  | IM | No | Initiate therapy with recommended starting dose. | Strong | (21) |
|  |  |  | PM | Yes | Consider a 50% reduction of recommended starting dose and titrate to response or select alternative drug not predominantly metabolized by CYP2C19. | Strong | (21) |
| CYP2C19 | Sertraline | CPIC: Yes | UM | No | Initiate therapy with recommended starting dose. If patient does not respond to recommended maintenance dosing, consider alternative drug not predominantly metabolized by CYP2C19. | Optional | (21) |
|  |  |  | NM | No | Initiate therapy with recommended starting dose. | Strong | (21) |
|  |  |  | IM | No | Initiate therapy with recommended starting dose. | Strong | (21) |
|  |  |  | PM | Yes | Consider a 50% reduction of recommended starting dose and titrate to response or select alternative drug not predominantly metabolized by CYP2C19. | Optional | (21) |
| CYP2D6 | Tricyclic antidepressants | CPIC: Yes | UM | Yes | Avoid tricyclic use due to potential lack of efficacy. Consider alternative drug not metabolized by CYP2D6. If a TCA is warranted, consider titrating to a higher target dose (compared to normal metabolizers). Utilize therapeutic drug monitoring to guide dose adjustments. | Strong (amitriptyline + nortriptyline)  Optional: other TCAs | (22) |
|  |  |  | NM | No | Initiate therapy with recommended starting dos | Strong | (22) |
|  |  |  | IM | Yes | Consider a 25% reduction of recommended starting dose. Utilize therapeutic drug monitoring to guide dose adjustments. | Moderate (amitriptyline + nortriptyline)  Optional: other TCAs | (22) |
|  |  |  | PM | Yes | Avoid tricyclic use due to potential for side effects. Consider alternative drug not metabolized by CYP2D6. If a TCA is warranted, consider a 50% reduction of recommended starting dose. Utilize therapeutic drug monitoring to guide dose adjustments. | Strong (amitriptyline + nortriptyline)  Optional: other TCAs | (22) |
| CYP2C19 | Amitriptyline, Clomipramine, Doxepine, Imipramine, and Trimipramine | CPIC: Yes | UM | Yes | Avoid tertiary amine use due to potential for sub-optimal response. Consider alternative drug not metabolized by CYP2C19. TCAs without major CYP2C19 metabolism include the secondary amines nortriptyline and desipramine. If a tertiary amine is warranted, utilize therapeutic drug monitoring to guide dose adjustments. | Optional | (22) |
|  |  |  | NM | No | Initiate therapy with recommended starting dose. | Strong | (22) |
|  |  |  | IM | No | Initiate therapy with recommended starting dose. | Strong (amitriptyline)  Optional: other TCAs | (22) |
|  |  |  | PM | Yes | Avoid tertiary amine use due to potential for sub-optimal response. Consider alternative drug not metabolized by CYP2C19. TCAs without major CYP2C19 metabolism include the secondary amines nortriptyline and desipramine. For tertiary amines, consider a 50% reduction of the recommended starting dose. Utilize therapeutic drug monitoring to guide dose adjustments. | Moderate (amitriptyline)  Optional: other TCAs | (22) |
| CYP2D6 and CYP2C19 | Amitriptyline | CPIC: Yes | CYP2C19 UM | Yes | CYP2D6 UM: Avoid amitriptyline  CYP2D6 NM: Consider alternative drug not metabolized by CYP2C19  CYP2D6 IM: Consider alternative drug not metabolized by CYP2C19  CYP2D6 PM: Avoid amitriptyline use | See TCAs | (22) |
|  |  |  | CYP2C19 NM | Yes | CYP2D6 UM: Avoid amitriptyline use. If amitriptyline is warranted, consider titrating to a higher target dose (compared to normal metabolizers  CYP2D6 NM: Initiate therapy with recommended starting dose  CYP2D6 IM: Consider a 25% reduction of recommended starting dose  CYP2D6 PM: Avoid amitriptyline use. If amitriptyline is warranted, consider a 50% reduction of recommended starting dose | See TCAs | (22) |
|  |  |  | CYP2C19 IM | Yes | CYP2D6 UM: Avoid amitriptyline  CYP2D6 NM: Initiate therapy with recommended starting dose  CYP2D6 IM: Consider a 25% reduction of recommended starting dose  CYP2D6 PM: Avoid amitriptyline use. If amitriptyline is warranted, consider a 50% reduction of recommended starting dose | See TCAs | (22) |
|  |  |  | CYP2C19 PM | Yes | CYP2D6 UM: Avoid amitriptyline  CYP2D6 NM: Avoid amitriptyline use. If amitriptyline is warranted, consider a 50% reduction of recommended starting dose  CYP2D6 IM: Avoid amitriptyline  CYP2D6 PM: Avoid amitriptyline | See TCAs | (22) |
| CYP3A5 | Tacrolimus | CPIC: Yes | NM (CYP3A5 expressor) | Yes | Increase starting dose 1.5–2 times recommended starting dose. Total starting dose should not exceed 0.3 mg/kg/day. Use therapeutic drug monitoring to guide dose adjustments | Strong | (23) |
|  |  |  | IM (CYP3A5 expressor) | Yes | Increase starting dose 1.5–2 times recommended starting dose. Total starting dose should not exceed 0.3 mg/kg/day. Use therapeutic drug monitoring to guide dose adjustments. | Strong | (23) |
|  |  |  | PM (CYP3A5 non expressor) | No | Initiate therapy with standard recommended dose. Use therapeutic drug monitoring to guide dose adjustments. | Strong | (23) |
| DPYD | Fluoro-pyrimidines | CPIC: Yes | NM | No | Based on genotype, there is no indication to change dose or therapy. Use labelrecommended dosage and administration. | Strong | (24) |
|  |  |  | IM | Yes | Reduce starting dose based on activity score followed by titration of dose based on toxicity or therapeutic drug monitoring (if available).  AS 1: Reduce dose by 50% Activity score  AS 1.5: Reduce dose by 50% | Strong  Moderate | (24) |
|  |  |  | PM | Yes | Activity score 0.5: Avoid use of 5-fluorouracil or 5-fluorouracil prodrug-based regimens. In the event, based on clinical advice, alternative agents are not considered a suitable therapeutic option, 5-fluorouracil should be administered at a strongly reduced dosed with early therapeutic drug monitoring. Activity score 0: Avoid use of 5-fluorouracil or 5-fluorouracil prodrug-based regimens. | Strong | (24) |
| G6PD | Rasburicase | CPIC: Yes | Normal | No | No reason to withhold rasburicase based on G6PD status | Strong | (25) |
|  |  |  | Deficient or deficient with CNSHA | Yes | Rasburicase is contraindicated; alternatives include allopurinol | Strong | (25) |
|  |  |  | Variable | Yes | To ascertain that G6PD status is normal, enzyme activity must be measured; alternatives include allopurinol | Moderate | (25) |
| HLA-A HLA-B | Carbamazepine | CPIC: Yes | HLA-B*15:02 negative and HLA-A*31:01 negative | No | Use carbamazepine per standard dosing guidelines | Strong | (26) |
|  |  |  | HLA-B*15:02 negative and HLA-A*31:01 positive | Yes | If patient is carbamazepine-naïve and alternative agents are available, do not use carbamazepine. | Strong | (26) |
|  |  |  | HLA-B*15:02 positive and any HLA-A*31:01 genotype (or HLA-A*31:01 genotype unknown) | Yes | If patient is carbamazepine-naïve, do not use carbamazepine. | Strong | (26) |
| HLA-B | Oxcarbazepine | CPIC: Yes | HLA-B*15:02 negative | No | Use oxcarbazepine per standard dosing guidelines | Strong | (26) |
|  |  |  | HLA-B*15:02 positive | Yes | If patient is oxcarbazepine naïve, do not use oxcarbazepine. | Strong | (26) |
| HLA-B | Abacavir | CPIC: Yes | Noncarrier of HLA-B*57:01 | No | Use abacavir per standard dosing guidelines | Strong | (27) |
|  |  |  | Carrier of HLA-B*57:01 | Yes | Abacavir is not recommended. | Strong | (27) |
| HLA-B | Allopurinol | CPIC: Yes | Noncarrier of HLA-B*5801 | No | Use allopurinol per standard dosing guidelines | Strong | (28) |
|  |  |  | Carrier of HLA-B*5801 | Yes | Allopurinol is contraindicated. | Strong | (28) |
| IFNL3 | PEG-IFN-α–containing regimens | CPIC: Yes | Favorable response genotype | No | Approximately 90% chance for SVR after 24–48 weeks of treatment. Approximately 80–90% of patients are eligible for shortened therapy (24–28 weeks vs. 48 weeks). Weighs in favor of using PEG-IFN-α- and RBV- containing regimens. | Strong | (29) |
|  |  |  | Unfavorable response genotype | Yes | Approximately 60% chance of SVRc after 24–48 weeks of treatment. Approximately 50% of patients are eligible for shortened therapy regimens (24–28 weeks). Consider implications before initiating PEG-IFN-α- and RBV-containing regimens. | Strong | (29) |
| RYR1/CACNA1S | Potent volatile anesthetic agents,  Succinylcholine | CPIC: Yes | MHS (malignant hyperthermia susceptibility) | Yes | Halogenated volatile anesthetics or depolarizing muscle relaxants succinylcholine are relatively contraindicated in persons with MHS. They should not be used, except in extraordinary circumstances in which the benefits outweigh the risks. In general, alternative anesthetics are widely available and effective in patients with MHS | Strong | (30) |
|  |  |  | Uncertain susceptibility | Yes | Clinical findings, family history, further genetic testing, and other laboratory data should guide use of halogenated volatile anesthetics or depolarizing muscle relaxants | Strong | (30) |
| SLC01B1 | Atorvastatin | CPIC: No | SLCO1B1*5  rs4149056 C  rs4149056 TT | No | No action is recommended. | - | (31) |
| SLC01B1 | Simvastatin | CPIC: Yes | Normal function | No | Prescribe desired starting dose and adjust doses of simvastatin based on disease-specific guidelines | Strong | (31) |
|  |  |  | Intermediate function | Yes | Prescribe a lower dose or consider an alternative statin (e.g., pravastatin or rosuvastatin); consider routine CK surveillance | Strong | (31) |
|  |  |  | Low function | Yes | Prescribe a lower dose or consider an alternative statin (e.g., pravastatin or rosuvastatin); consider routine CK surveillance | Strong | (31) |
| TPMT | Mercaptopurine | CPIC: Yes | NM | No | Start with normal starting dose (e.g., 75 mg/m2/day or 1.5 mg/kg/day) and adjust doses of mercaptopurine (and of any other myelosuppressive therapy) without any special emphasis on mercaptopurine compared with other agents. Allow at least 2 weeks to reach steady-state after each dose adjustment. | Strong | (32) |
|  |  |  | IM | Yes | Start with reduced starting doses (30–80% of normal dose) if normal starting dose is ≥ 75 mg/m2/day or ≥ 1.5 mg/kg/day (e.g., start at 22.5–60 mg/m2 / day or 0.45–1.2 mg/kg/ day) and adjust doses of mercaptopurine based on degree of myelosuppression and disease-specific guidelines. Allow 2–4 weeks to reach steady-state after each dose adjustment. If myelosuppression occurs, and depending on other therapy, emphasis should be on reducing mercaptopurine over other agents. If normal starting dose is already < 75 mg/m2/day or < 1.5 mg/kg/day, dose reduction may not be recommended | Strong | (32) |
|  |  |  | PM | Yes | For malignancy, start with drastically reduced doses (reduce daily dosea by 10-fold and reduce frequency to thrice weekly instead of daily (e.g., 10 mg/m2/ day given just 3 days/ week) and adjust doses of mercaptopurine based on degree of myelosuppression and disease-specific guidelines. Allow 4–6 weeks to reach steady-state after each dose adjustment. If myelosuppression occurs, emphasis should be on reducing mercaptopurine over other agents. For nonmalignant conditions, consider alternative nonthiopurine immunosuppressant therapy. | Strong | (32) |
| TPMT | Azathioprine | CPIC: Yes | NM | No | Start with normal starting dose (e.g., 2–3 mg/kg/day) and adjust doses of azathioprine based on disease-specific guidelines. Allow 2 weeks to reach steady-state after each dose adjustment. | Strong | (32) |
|  |  |  | IM | Yes | Start with reduced starting doses (30–80% of normal dose) if normal starting dose is 2–3 mg/kg/ day (e.g., 0.6–2.4 mg/ kg/day), and adjust doses of azathioprine based on degree of myelosuppression and disease-specific guidelines. Allow 2–4 weeks to reach steady-state after each dose adjustment. | Strong | (32) |
|  |  |  | PM | Yes | For nonmalignant conditions, consider alternative nonthiopurine immunosuppressant therapy. For malignancy, start with drastically reduced doses (reduce daily dose by 10-fold and dose thrice weekly instead of daily) and adjust doses of azathioprine based on degree of myelosuppression and disease-specific guidelines. Allow 4–6 weeks to reach steady-state after each dose adjustment | Strong | (32) |
| TPMT | Thioguanine | CPIC: Yes | NM | No | Start with normal starting dose (e.g., 40–60 mg/m2/day) and adjust doses of thioguanine and of other myelosuppressive therapy without any special emphasis on thioguanine. Allow 2 weeks to reach steady-state after each dose adjustment. | Strong | (32) |
|  |  |  | IM | Yes | Start with reduced doses (50–80% of normal dose) if normal starting dose is ≥ 40–60 mg/m2/day (e.g., 20–48 mg/ m2/day) and adjust doses of thioguanine based on degree of myelosuppression and disease-specific guidelines. Allow 2–4 weeks to reach steady-state after each dose adjustment. If myelosuppression occurs, and depending on other therapy, emphasis should be on reducing thioguanine over other agents. | Moderate | (32) |
|  |  |  | PM | Yes | Start with drastically reduced doses16 (reduce daily dose by 10-fold and dose thrice weekly instead of daily) and adjust doses of thioguanine based on degree of myelosuppression and disease-specific guidelines. Allow 4–6 weeks to reach steady-state after each dose adjustment. If myelosuppression occurs, emphasis should be on reducing thioguanine over other agents. For nonmalignant conditions, consider alternative nonthiopurine immunosuppressant therapy. | Strong | (32) |
| NUDT15 | Mercaptopurine | CPIC: Yes | NM | No | Start with normal starting dose (e.g., 75 mg/m2/day or 1.5 mg/kg/day) and adjust doses of mercaptopurine (and of any other myelosuppressive therapy) without any special emphasis on mercaptopurine compared with other agents. Allow at least 2 weeks to reach steady-state after each dose adjustment. | Strong | (32) |
|  |  |  | IM | Yes | Start with reduced starting doses (30–80% of normal dose) if normal starting dose is ≥ 75 mg/m2/day or ≥ 1.5 mg/kg/day (e.g., start at 22.5–60 mg/m2 / day or 0.45–1.2 mg/kg/ day) and adjust doses of mercaptopurine based on degree of myelosuppression and disease-specific guidelines. Allow 2–4 weeks to reach steady-state after each dose adjustment. If myelosuppression occurs, and depending on other therapy, emphasis should be on reducing mercaptopurine over other agents. If normal starting dose is already < 75 mg/m2/day or < 1.5 mg/kg/day, dose reduction may not be recommended. | Strong | (32) |
|  |  |  | PM | Yes | For malignancy, initiate dose at 10 mg/m2/day and adjust dose based on myelosuppression and disease-specific guidelines. Allow 4–6 weeks to reach steady state after each dose adjustment. If myelosuppression occurs, emphasis should be on reducing mercaptopurine over other agents. For nonmalignant conditions, consider alternative nonthiopurine immunosuppressant therapy. | Strong | (32) |
| NUDT15 | Azathioprine | CPIC: Yes | NM | No | Start with normal starting dose (e.g., 2–3 mg/kg/day) and adjust doses of azathioprine based on disease-specific guidelines. Allow 2 weeks to reach steady-state after each dose adjustment. | Strong | (32) |
|  |  |  | IM | Yes | Start with reduced starting doses (30–80% of normal dose) if normal starting dose is 2–3 mg/kg/day (e.g., 0.6–2.4 mg/kg/day), and adjust doses of azathioprine based on degree of myelosuppression and disease-specific guidelines. Allow 2–4 weeks to reach steady-state after each dose adjustment. | Strong | (32) |
|  |  |  | PM | Yes | For nonmalignant conditions, consider alternative nonthiopurine immunosuppressant therapy. For malignant conditions, start with drastically reduced normal daily doses (reduce daily dose by 10-fold) and adjust doses of azathioprine based on degree of myelosuppression and disease-specific guidelines. Allow 4–6 weeks to reach steady-state after each dose adjustment. | Strong | (32) |
| NUDT15 | Thioguanine | CPIC: Yes | NM | No | Start with normal starting dose (40– 60 mg/m2/day). Adjust doses of thioguanine and of other myelosuppressive therapy without any special emphasis on thioguanine. Allow 2 weeks to reach steady-state after each dose adjustment. | Strong | (32) |
|  |  |  | IM | Yes | Start with reduced doses (50–80% of normal dose) if normal starting dose is ≥ 40–60 mg/m2/day (e.g., 20–48 mg/m2 / day) and adjust doses of thioguanine based on degree of myelosuppression and disease-specific guidelines. Allow 2–4 weeks to reach steady-state after each dose adjustment. If myelosuppression occurs, and depending on other therapy, emphasis should be on reducing thioguanine over other agents. | Moderate | (32) |
|  |  |  | PM | Yes | Reduce doses to 25% of normal dose and adjust doses of thioguanine based on degree of myelosuppression and disease-specific guidelines. Allow 4–6 weeks to reach steady-state after each dose adjustment. In setting of myelosuppression, emphasis should be on reducing thioguanine over other agents. For nonmalignant conditions, consider alternative nonthiopurine immunosuppressant therapy. | Strong | (32) |
| UGT1A1 | Atazanivir | CPIC: Yes | NM | No | There is no need to avoid prescribing of atazanavir based on UGT1A1 genetic test result. Inform the patient that some patients stop atazanavir because of jaundice (yellow eyes and skin), but that this patient’s genotype makes this unlikely (less than about a 1 in 20 chance of stopping atazanavir because of jaundice). | Strong | (33) |
|  |  |  | IM | No | There is no need to avoid prescribing of atazanavir based on UGT1A1 genetic test result. Inform the patient that some patients stop atazanavir because of jaundice (yellow eyes and skin), but that this patient’s genotype makes this unlikely (less than about a 1 in 20 chance of stopping atazanavir because of jaundice). | Strong | (33) |
|  |  |  | PM | Yes | Consider an alternative agent particularly where jaundice would be of concern to the patient. If atazanavir is to be prescribed, there is a high likelihood of developing jaundice that will result in atazanavir discontinuation (at least 20% and as high as 60%). | Strong | (33) |
|  |  |  |  |  |  |  |  |
| CYP2C9 | Warfarin | CPNDS: Yes | *2 | Yes | Calculate dose based on validated published pharmacogenetic algorithm. Accessible via www.warfarindosing.org | ++++  B - Moderate | (34) |
|  |  |  | *3 | Yes | Calculate dose based on validated published pharmacogenetic algorithm. Accessible via www.warfarindosing.org | ++++  B - Moderate | (34) |
| VKORC1 | Warfarin | CPNDS: Yes | -1639AA | Yes | Calculate dose based on validated published pharmacogenetic algorithm. Accessible via www.warfarindosing.org | ++++  B - Moderate | (34) |
|  |  |  | -1639GA | Yes | Calculate dose based on validated published pharmacogenetic algorithm. Accessible via www.warfarindosing.org | ++++  B - Moderate | (34) |
| CYP2D6 | Tamoxifen | CPNDS: Yes | PM | Yes | Tamoxifen 40mg/day | +++  B - Moderate | (35) |
|  |  |  | IM | Yes | Tamoxifen 40mg/day | +++  B - Moderate | (35) |
| RARG | Doxorubicin | CPNDS: Yes | Rs2229774A | Yes | Patients with this phenotype are at high risk of anthracycline associated cardiotoxicity:  Increase frequency of monitoring with serial yearly echocardiographic monitoring a follow-up as recommended by COG guidelines. Aggressive screening and management of cardiovascular risk factors including obesity, diabetes, hypertension, coronary artery idea, lipid disorders and peripheral vascular disease. Prescribe dexrazoxane. Use liposomal encapsulated anthracycline preparations. Use of continuous inclusion or slower inclusion rates. Use of less cardiotoxic types of anthracyclines. Use of other cardioprotective agents. Prescribe alternative chemotherapy regiments for certain tumor types where alternative regiments have been shown to be equally effective. | +++  B - Moderate | (36) |
| SLC28A3 | Doxorubucin | CPNDS: Yes | rs7853758A | No | Patients with this phenotype are at low risk of anthracycline associated cardiotoxicity: a normal follow-up is recommended. | +++  B - Moderate | (36) |
| UGT1A6 | Doxorubicin | CPNDS: Yes | rs17863783T | Yes | Patients with this phenotype are at high risk of anthracycline associated cardiotoxicity:  Increase frequency of monitoring with serial yearly echocardiographic monitoring a follow-up as recommended by COG guidelines. Aggressive screening and management of cardiovascular risk factors including obesity, diabetes, hypertension, coronary artery idea, lipid disorders and peripheral vascular disease. Prescribe dexrazoxane. Use liposomal encapsulated anthracycline preparations. Use of continuous inclusion or slower inclusion rates. Use of less cardiotoxic types of anthracyclines. Use of other cardioprotective agents. Prescribe alternative chemotherapy regiments for certain tumor types where alternative regiments have been shown to be equally effective. | +++  B - Moderate | (36) |
| RARG | Daunorubicin | CPNDS: Yes | rs2229774A | Yes | Patients with this phenotype are at high risk of anthracycline associated cardiotoxicity:  Increase frequency of monitoring with serial yearly echocardiographic monitoring a follow-up as recommended by COG guidelines. Aggressive screening and management of cardiovascular risk factors including obesity, diabetes, hypertension, coronary artery idea, lipid disorders and peripheral vascular disease. Prescribe dexrazoxane. Use liposomal encapsulated anthracycline preparations. Use of continuous inclusion or slower inclusion rates. Use of less cardiotoxic types of anthracyclines. Use of other cardioprotective agents. Prescribe alternative chemotherapy regiments for certain tumor types where alternative regiments have been shown to be equally effective. | +++  B - Moderate | (36) |
| SLC28A3 | Daunorubicin | CPNDS: Yes | rs7853758A | No | Patients with this phenotype are at low risk of anthracycline associated cardiotoxicity: a normal follow-up is recommended. | +++  B - Moderate | (36) |
| UGT1A6 | Daunorubicin | CPNDS: Yes | rs2229774A | Yes | Patients with this phenotype are at high risk of anthracycline associated cardiotoxicity:  Increase frequency of monitoring with serial yearly echocardiographic monitoring a follow-up as recommended by COG guidelines. Aggressive screening and management of cardiovascular risk factors including obesity, diabetes, hypertension, coronary artery idea, lipid disorders and peripheral vascular disease. Prescribe dexrazoxane. Use liposomal encapsulated anthracycline preparations. Use of continuous inclusion or slower inclusion rates. Use of less cardiotoxic types of anthracyclines. Use of other cardioprotective agents. Prescribe alternative chemotherapy regiments for certain tumor types where alternative regiments have been shown to be equally effective. | +++  B - Moderate | (36) |
| CYP2D6 | Codeine | CPNDS: Yes | IM/NM | No | Codeine can be used as per standard of care. Existing evidence suggests that caution is still warranted in CYP2D6 NMs receiving codeine if they are receiving maximal therapeutic doses of codeine and have additional risk factors for toxicity. | ++++  B - Moderate | (37) |
|  |  |  | PM | Yes | Poor metabolizers of CYP2D6 should not receive codeine for pain relief | ++++  A - Strong | (37) |
|  |  |  | UM | Yes | Ultra rapid metabolizers of CYP2D6 should avoid codeine for pain relief and receive alternative analgesics that do not have potent CYP2D6 metabolites | ++++  B - Moderate | (37) |
| TPMT | Cisplatin | CPNDS: Yes | *2 | Yes | Physicians are encouraged to consider the use of otoprotectants (i.e. amifostine, sodium thiosulfate) if the patient’s tumour type is one for which otoprotectants may be effective to prevent cisplatin-induced ototoxicity without adversely affecting antitumour activity.  Alternative treatments may be prescribed when they have demonstrated equal efficacy, manageable and acceptable toxicity, less ototoxicity, and are considered options within the current standards of care.  Where appropriate, physicians are encouraged to increase monitoring in high-risk patients.  High risk patients should be encouraged to receive more frequent follow-up audiometric hearing tests after treatment has ended.  Pediatric patietnts should be tested for TPMT *2 variant. | +++  A - Strong | (38) |
|  |  |  | *3A | Yes | Physicians are encouraged to consider the use of otoprotectants (i.e. amifostine, sodium thiosulfate) if the patient’s tumour type is one for which otoprotectants may be effective to prevent cisplatin-induced ototoxicity without adversely affecting antitumour activity.  Alternative treatments may be prescribed when they have demonstrated equal efficacy, manageable and acceptable toxicity, less ototoxicity, and are considered options within the current standards of care.  Where appropriate, physicians are encouraged to increase monitoring in high-risk patients.  High risk patients should be encouraged to receive more frequent follow-up audiometric hearing tests after treatment has ended. | +++  A - Strong | (38) |
|  |  |  | *3B | Yes | Physicians are encouraged to consider the use of otoprotectants (i.e. amifostine, sodium thiosulfate) if the patient’s tumour type is one for which otoprotectants may be effective to prevent cisplatin-induced ototoxicity without adversely affecting antitumour activity.  Alternative treatments may be prescribed when they have demonstrated equal efficacy, manageable and acceptable toxicity, less ototoxicity, and are considered options within the current standards of care.  Where appropriate, physicians are encouraged to increase monitoring in high-risk patients.  High risk patients should be encouraged to receive more frequent follow-up audiometric hearing tests after treatment has ended. | +++  A - Strong | (38) |
|  |  |  | *3C | Yes | Physicians are encouraged to consider the use of otoprotectants (i.e. amifostine, sodium thiosulfate) if the patient’s tumour type is one for which otoprotectants may be effective to prevent cisplatin-induced ototoxicity without adversely affecting antitumour activity.  Alternative treatments may be prescribed when they have demonstrated equal efficacy, manageable and acceptable toxicity, less ototoxicity, and are considered options within the current standards of care.  Where appropriate, physicians are encouraged to increase monitoring in high-risk patients.  High risk patients should be encouraged to receive more frequent follow-up audiometric hearing tests after treatment has ended. | +++  A - Strong | (38) |
| HLA-A | Carbamazepine | CPNDS: Yes | *13:01 | Yes | In patients who are positive for HLA-A*31:01, alternative medication should be used as first-line therapy | +++  B - Moderate | (39) |
| HLA-B | Carbamazepine | CPNDS: Yes | *15:02 | Yes | In patients who are positive for HLA-A*15:02, alternative medication should be used as first-line therapy | +++  B - Moderate | (39) |
|  |  |  |  |  |  |  |  |
| UGT1A1 | Irinotecan | RNPGx: Yes | *28 | No | At low doses, (<180 mg/m^2^/week), the presence of the *UGT1A1*28* allele is not a major risk factor (little difference in risk of hematological or digestive toxicity irrespective of the genotype)  180—230 mg/m^2^ spaced by 2—3-week intervals  Patients who are homozygous for the *UGT1A1*28* allele have a higher risk of hematological and/or digestive toxicity than patients who are heterozygous or non-carriers. For these **28/*28* patients, a 25-30% dose reduction is recommended, especially if the patient presents other risk factors for toxicity. Dose can be adjusted for subsequent cycles depending on the tolerance  240 mg/m^2^ or higher spaced by 2—3-week intervals  Homozygous *UGT1A1*28* patients have a greatly increased risk of hematological toxicity (neutropenia) compared with other genotypes, contraindicating administration at this higher dose and leading to discussion of a standard dose depending on the associated risk factors. Administration of an intensive dose (240 mg/m^2^) is recommended only for **1/*1* patients, or for **1/*28* patients who have no other risk factors and who benefit from intensive surveillance. | Advisable  Essential | (40) |
| CYP2C19 | Clopidogrel | RNPGx: Yes | UM | No | Use as per standard of care, 75mg/day | Essential | (41) |
|  |  |  | NM | No | Use as per standard of care, 75mg/day | Essential | (41) |
|  |  |  | IM | Yes | Prescribe an alternative drug, not metabolized by CYP2C19 | Essential | (41) |
|  |  |  | PM | Yes | Prescribe an alternative drug, not metabolized by CYP2C19 | Essential | (41) |
| CYP2C9/VKORC1 | Warfarin | RNPGx: Yes | *1/*1 | Yes | VKORC1 genotype GG 5-7 mg warfarin/day  VKORC1 genotype GA 5-7 mg warfarin/day  VKORC1 genotype AA 3-4 mg warfarin/day | Advisable | (41) |
|  |  |  | *1/*2 | Yes | VKORC1 genotype GG 5-7 mg warfarin/day  VKORC1 genotype GA 3-4 mg warfarin/day  VKORC1 genotype AA 3-4 mg warfarin/day | Advisable | (41) |
|  |  |  | *1/*3 | Yes | VKORC1 genotype GG 3-4 mg warfarin/day  VKORC1 genotype GA 3-4 mg warfarin/day  VKORC1 genotype AA 0.5-2 mg warfarin/day | Advisable | (41) |
|  |  |  | *2/*2 | Yes | VKORC1 genotype GG 3-4 mg warfarin/day  VKORC1 genotype GA 3-4 mg warfarin/day  VKORC1 genotype AA 0.5-2 mg warfarin/day | Advisable | (41) |
|  |  |  | *2/*3 | Yes | VKORC1 genotype GG 3-4 mg warfarin/day  VKORC1 genotype GA 0.5-2 mg warfarin/day  VKORC1 genotype AA 0.5-2 mg warfarin/day | Advisable | (41) |
|  |  |  | *3/*3 | Yes | VKORC1 genotype GG 0.5-2 mg warfarin/day  VKORC1 genotype GA 0.5-2 mg warfarin/day  VKORC1 genotype AA 0.5-2 mg warfarin/day | Advisable | (41) |
| CYP3A5 | Tacrolimus | RNPGx: Yes | *1/*1 | Yes | Based on TDM 1.5 to 2 times the dose recommended to non-expressors; maximum dose 0.30mg/kg/day | Advisable | (42) |
|  |  |  | *1/*3 | Yes | Based on TDM 1.5 to 2 times the dose recommended to non-expressors; maximum dose 0.30mg/kg/day | Advisable | (42) |
|  |  |  | *3/*3 | Yes | Based on TDM 0.15 mg/kg/day | Advisable | (42) |
|  |  |  | *22 | Yes | Adapt the initial tacrolimus dose. | Possibly helpful | (43) |
| SLCO1B1 | HMG coareductase inhibitors | RNPGx: Yes | *5/*5 | Yes | High dose statins should be avoided, as well as OATP1B1 and/or CYP3A4 inhibitors; lower simvastatin dose to 20 mg per day plus CPK assay or select another statin. | Possibly helpful | (41) |
|  |  |  | *1/*5 | Yes | High dose statins should be avoided, as well as OATP1B1 and/or CYP3A4 inhibitors | Possibly helpful | (41) |
| DPYD | Capecitabine and Fluorouracil | RNPGx: Yes | Heterozygous  *2A / *13  2846A>T | Yes | In the first cycle, reduce the dose by 50% | Essential | (40) |
|  |  |  | Homozygous  *2A / *13  2846A>T | Yes | Capecitabine and fluorouracil are contraindicated | Essential | (40) |
| CYP2C19 | Tricyclic  antidepressants | RNPGx: Yes | UM | Yes | Preferred prescription of antidepressants not metabolized by CYP2D6 If tricyclic antidepressants are continued, propose TDM to adapt dose | Advisable | (44) |
|  |  |  | NM | No | Initiate treatment at recommended standard dose | Advisable | (44) |
|  |  |  | IM | No | Initiate treatment at recommended standard dose | Advisable | (44) |
|  |  |  | PM | Yes | Propose: 50% lower dose at treatment onset; TDM to adapt dose | Advisable | (44) |
| CYP2D6 | Tricyclic antidepressants | RNPGx: Yes | UM | Yes | Avoid using tricyclic antidepressants due to the risk of inefficacy Preferred prescription of antidepressants not metabolized by CYP2D6 If tricyclic antidepressants are continued, it is proposed to: increase dose at treatment onset; adapt dose according to TDM findings | Advisable | (44) |
|  |  |  | NM | No | Initiate treatment at recommended standard dose | Advisable | (44) |
|  |  |  | IM | Yes | Propose: 50% lower dose at treatment onset; TDM to adapt dose | Advisable | (44) |
|  |  |  | PM | Yes | Avoid using tricyclic antidepressants due to the risk of poor tolerance Preferred prescription of antidepressants not metabolized by CYP2D6 If tricyclic antidepressants are continued, it is proposed to: increase dose at treatment onset; adapt dose according to TDM findings | Advisable | (44) |

# Recommendations included in the table are as of July 1, 2020. Guidelines are being updated and new guidelines are being developed regularly.

DPWG has five (0-4) levels of evidence, classifies the clinical relevance on an eight point scale (AA^#^ to F), and has three genotyping recommendations (essential, beneficial, and potentially beneficial). (45,46) * classification of genotyping by the clinical implication score is expected to be performed in the next recommendation’s update.

CPIC has three levels for pharmacotherapeutic recommendations for genotype/phenotype-drug pairs: S= Strong, M=moderate, and O=optional. (47)

CPNDS has four levels of evidence (+ to ++++), and three levels for genotyping recommendations: S= Strong=A, M=moderate=B, and O=optional=C. (39)

RNPGx has three levels of for genotyping recommendations: essential test, advisable test, and possibly helpful test. (48)

PEG-IFN- α: PEG Interferon-Alpha. TDM: Therapeutic Drug Monitoring. UM: ultrarapid metabolizer. RM: rapid metabolizer. NM: normal metabolizer. IM: intermediate metabolizer. PM: poor metabolizer.

Extensive metabolizer has been renamed as NM. AS: activity score.

DRESS = drug reaction with eosinophilia and systemic symptoms, also known as hypersensitivity syndrome (HSS).

SJS = Stevens-Johnson syndrome, TEN = toxic epidermal necrolysis.

1. The Dutch Pharmacogenomic Working Group (DPWG). Phamacogenomic recommendations, farmacogenetica-update [Internet]. 2020 [cited 2020 Jul 12]. Available from: www.knmp.nl/

2. The Dutch Pharmacogenomic Working Group (DPWG). HLA: allopurinol [Internet]. 2017. p. 1–18. Available from: https://www.g-standaard.nl/risicoanalyse/B0006391.PDF

3. Swen J, Nijenhuis M, De Boer A, Grandia L, Maitland-van der Zee A, Mulder H, et al. Pharmacogenetics: From Bench to Byte— An Update of Guidelines. Clin Pharmacol Ther |. 2011;89(5):662–73.

4. The Dutch Pharmacogenomic Working Group (DPWG). HLA: carbamazepine [Internet]. 2018. p. 1–16. Available from: https://www.g-standaard.nl/risicoanalyse/B0006237.PDF

5. The Dutch Pharmacogenomic Working Group (DPWG). HLA: lamotrigine [Internet]. 2018 [cited 2020 Jul 21]. p. 1–13. Available from: https://www.g-standaard.nl/risicoanalyse/B0006932.PDF

6. The Dutch Pharmacogenomic Working Group (DPWG). HLA: oxcarbazepine [Internet]. 2018 [cited 2020 Jul 21]. p. 1–10. Available from: https://www.g-standaard.nl/risicoanalyse/B0006931.PDF

7. Pharmacogenetics T, Group W, Asian S, Chinese H, Sjs M, Sjs HC, et al. HLA : phenytoin. 2017;

8. The Dutch Pharmacogenomic Working Group (DPWG). CYP2C9: warfarin [Internet]. 2016 [cited 2020 Jul 11]. p. 1–16. Available from: https://www.g-standaard.nl/risicoanalyse/B0006234.PDF

9. Clancy JP, Johnson SG, Yee SW, McDonagh EM, Caudle KE, Klein TE, et al. Clinical Pharmacogenetics Implementation Consortium (CPIC) Guidelines for Ivacaftor Therapy in the Context of CFTR Genotype. 2014 [cited 2020 Jul 10]; Available from: http://www.genet.sickkids.on.ca/

10. Desta Z, Gammal RS, Gong L, Whirl-Carrillo M, Gaur AH, Sukasem C, et al. Clinical Pharmacogenetics Implementation Consortium (CPIC) Guideline for CYP2B6 and Efavirenz-Containing Antiretroviral Therapy. 2019 [cited 2020 Jul 10];106. Available from: www.cpt-journal.com

11. Scott S, Sangkuhl K, Stein C, Hulot J, Mega J, Roden D, et al. Clinical Pharmacogenetics Implementation Consortium Guidelines for CYP2C19 Genotype and Clopidogrel Therapy: 2013 Update. 2013 [cited 2020 Jul 10]; Available from: www.pharmgkb.

12. Lima JJ, Thomas CD, Barbarino J, Desta Z, Van Driest SL, El Rouby N, et al. Clinical Pharmacogenetics Implementation Consortium (CPIC) Guideline for CYP2C19 and Proton Pump Inhibitor Dosing. Clin Pharmacol Ther [Internet]. 2020 Aug 8; Available from: http://www.ncbi.nlm.nih.gov/pubmed/32770672

13. Moriyama B, Obeng AO, Barbarino J, Penzak SR, Henning SA, Scott SA, et al. Clinical Pharmacogenetics Implementation Consortium (CPIC) Guidelines for CYP2C19 and Voriconazole Therapy. [cited 2020 Jul 10]; Available from: http://www.pharmgkb.organdhttps//cpicpgx.org/guidelines/.

14. Theken KN, Lee CR, Gong L, Caudle KE, Formea CM, Gaedigk A, et al. Clinical Pharmacogenetics Implementation Consortium Guideline (CPIC) for CYP2C9 and Nonsteroidal Anti-Inflammatory Drugs. Clin Pharmacol Ther | [Internet]. [cited 2020 Jul 10]; Available from: https://www.pharm

15. Karnes JH, Rettie AE, Somogyi AA, Huddart R, Fohner AE, Formea CM, et al. Clinical Pharmacogenetics Implementation Consortium (CPIC) Guideline for CYP2C9 and HLA-B Genotypes and Phenytoin Dosing: 2020 Update. Clin Pharmacol Ther [Internet]. 2020 Aug 11; Available from: http://www.ncbi.nlm.nih.gov/pubmed/32779747

16. Johnson JA, Caudle KE, Gong L, Whirl-Carrillo M, Stein CM, Scott SA, et al. Clinical Pharmacogenetics Implementation Consortium (CPIC) Guideline for Pharmacogenetics-Guided Warfarin Dosing: 2017 Update. [cited 2020 Jul 10]; Available from: www.cpt-journal.com

17. Brown JT, Bishop JR, Sangkuhl K, Nurmi EL, Mueller DJ, Dinh JC, et al. Clinical Pharmacogenetics Implementation Consortium Guideline for Cytochrome P450 (CYP)2D6 Genotype and Atomoxetine Therapy. 2019 [cited 2020 Jul 10];1. Available from: https://cpicpgx.org/guidelines

18. Crews K, Gaedigk A, Dunnenberger H, Leeder J, Klein T, Caudle K, et al. Clinical Pharmacogenetics Implementation Consortium Guidelines for Cytochrome P450 2D6 Genotype and Codeine Therapy: 2014 Update. 2014 [cited 2020 Jul 10]; Available from: www.cypalleles.ki.se/cyp2d6.htm,

19. Bell GC, Caudle KE, Whirl-Carrillo M, Gordon RJ, Hikino K, Gaedigk A, et al. Clinical Pharmacogenetics Implementation Consortium (CPIC) Guideline for CYP2D6 Genotype and Use of Ondansetron and Tropisetron. [cited 2020 Jul 10]; Available from: www.cpt-journal.com

20. Goetz MP, Sangkuhl K, Guchelaar H-J, Schwab M, Province M, Whirl-Carrillo M, et al. Clinical Pharmacogenetics Implementation Consortium (CPIC) Guideline for CYP2D6 and Tamoxifen Therapy. [cited 2020 Jul 10]; Available from: www.cpicpgx.org/guidelines/.

21. Hicks J, Bishop J, Sangkuhl K, Ji Y, Leckband S, Leeder J, et al. Clinical Pharmacogenetics Implementation Consortium (CPIC) Guideline for CYP2D6 and CYP2C19 Genotypes and Dosing of Selective Serotonin Reuptake Inhibitors. [cited 2020 Jul 10]; Available from: www.pharmgkb.org,

22. Hicks JK, Sangkuhl K, Swen JJ, Ellingrod VL, M€ Uller DJ, Shimoda K, et al. Clinical Pharmacogenetics Implementation Consortium Guideline (CPIC) for CYP2D6 and CYP2C19 Genotypes and Dosing of Tricyclic Antidepressants: 2016 Update. [cited 2020 Jul 10]; Available from: www.cpt-journal.com

23. Birdwell KA, Decker B, Barbarino JM, Peterson JF, Stein CM, Sadee W, et al. Clinical Pharmacogenetics Implementation Consortium (CPIC) Guidelines for CYP3A5 Genotype and Tacrolimus Dosing. [cited 2020 Jul 10]; Available from: www.pharmgkb.org

24. Amstutz U, Henricks LM, Offer SM, Barbarino J, Schellens JHM, Swen JJ, et al. Clinical Pharmacogenetics Implementation Consortium (CPIC) Guideline for Dihydropyrimidine Dehydrogenase Genotype and Fluoropyrimidine Dosing: 2017 Update. [cited 2020 Jul 10]; Available from: www.cpt-journal.com

25. Relling M, McDonagh E, Chang T, Caudle K, McLeod H, Haidar C, et al. Clinical Pharmacogenetics Implementation Consortium (CPIC) Guidelines for Rasburicase Therapy in the Context of G6PD Deficiency Genotype. 2014 [cited 2020 Jul 10]; Available from: https://www.pharmgkb.org/pathway/

26. Phillips EJ, Sukasem C, Whirl-Carrillo M, M DJ, Dunnenberger HM, Chantratita W, et al. Clinical Pharmacogenetics Implementation Consortium Guideline for HLA Genotype and Use of Carbamazepine and Oxcarbazepine: 2017 Update. [cited 2020 Jul 10]; Available from: https://cpicpgx.org/guidelines

27. Martin MA, Klein TE, Dong BJ, Pirmohamed M, Haas DW, Kroetz DL. Clinical Pharmacogenetics Implementation Consortium Guidelines for HLA-B Genotype and Abacavir Dosing. 2012 [cited 2020 Jul 10]; Available from: http://aidsinfo.nih.gov.

28. Hershfield M, Callaghan J, Tassaneeyakul W, Mushiroda T, Thorn C, Klein T, et al. Clinical Pharmacogenetics Implementation Consortium Guidelines for Human Leukocyte Antigen-B Genotype and Allopurinol Dosing. 2012 [cited 2020 Jul 10]; Available from: www.pharmgkb.org

29. Muir A, Gong L, Johnson S, Lee M, Williams M, Klein T, et al. Clinical Pharmacogenetics Implementation Consortium (CPIC) Guidelines for IFNL3 (IL28B) Genotype and PEG Interferon-α–Based Re. 2013 [cited 2020 Jul 10]; Available from: http://www.who.

30. Gonsalves SG, Dirksen RT, Sangkuhl K, Pulk R, Alvarellos M, Vo T, et al. Clinical Pharmacogenetics Implementation Consortium (CPIC) Guideline for the Use of Potent Volatile Anesthetic Agents and Succinylcholine in the Context of RYR1 or CACNA1S Genotypes. 2019 [cited 2020 Jul 10];1. Available from: https://cpicpgx.org/guidelines

31. Ramsey L, Johnson S, Caudle K, Haidar C, Voora D, Wilke R, et al. The Clinical Pharmacogenetics Implementation Consortium Guideline for SLCO1B1 and Simvastatin-Induced Myopathy: 2014 Update. 2014 [cited 2020 Jul 10]; Available from: http://www.pharmgkb.org.

32. Relling1 M V., , Matthias Schwab2, 3, 4 MW-C, Suarez-Kurtz6 G, Pui7 C-H, Stein8 CM, , Ann M. Moyer9 WEE, et al. Clinical Pharmacogenetics Implementation Consortium Guideline for Thiopurine Dosing Based on TPMT and NUDT15 Genotypes: 2018 Update. Clin Pharmacol Ther. 2019;105(5):1095–105.

33. Gammal1 R, Court2 M, Haidar1 C, , OF Iwuchukwu3, 4 AG, Alvarellos6 M, Guillemette7 C, et al. Clinical Pharmacogenetics Implementation Consortium (CPIC) Guideline for UGT1A1 and Atazanavir Prescribing. Clin Pharmacol Ther. 2015;00(00):1–7.

34. Shaw K, Amstutz U, Kim RB, Lesko LJ, Turgeon J, Michaud V, et al. Clinical Practice Recommendations on Genetic Testing of CYP2C9 and VKORC1 Variants in Warfarin Therapy [Internet]. Vol. 37, Therapeutic Drug Monitoring. Lippincott Williams and Wilkins; 2015 [cited 2020 Jul 6]. p. 428–36. Available from: http://journals.lww.com/00007691-201508000-00002

35. Drögemöller BI, Wright GEB, Shih J, Monzon JG, Gelmon KA, Ross CJD, et al. CYP2D6 as a treatment decision aid for ER-positive non-metastatic breast cancer patients: a systematic review with accompanying clinical practice guidelines [Internet]. Vol. 173, Breast Cancer Research and Treatment. Springer New York LLC; 2019 [cited 2020 Jul 6]. p. 521–32. Available from: http://link.springer.com/10.1007/s10549-018-5027-0

36. Aminkeng F, Ross CJD, Rassekh SR, Hwang S, Rieder MJ, Bhavsar AP, et al. Recommendations for genetic testing to reduce the incidence of anthracycline-induced cardiotoxicity [Internet]. Vol. 82, British Journal of Clinical Pharmacology. Blackwell Publishing Ltd; 2016 [cited 2020 Jul 6]. p. 683–95. Available from: https://onlinelibrary.wiley.com/doi/abs/10.1111/bcp.13008

37. Parvaz Madadi UAMRSIVFSHJTVMGKBCCCCRG. Clinical Practice Guideline: CYP2D6 Genotyping for Safe and Efficacious Codeine Therapy - PubMed. J Popul Ther Clin Pharmacol [Internet]. 2013 Nov 6 [cited 2020 Jul 6];20(3):369–96. Available from: https://pubmed.ncbi.nlm.nih.gov/24214521/

38. Lee JW, Pussegoda K, Rassekh SR, Monzon JG, Liu G, Hwang S, et al. Clinical Practice Recommendations for the Management and Prevention of Cisplatin-Induced Hearing Loss Using Pharmacogenetic Markers [Internet]. Vol. 38, Therapeutic Drug Monitoring. Lippincott Williams and Wilkins; 2016 [cited 2020 Jul 6]. p. 423–31. Available from: http://journals.lww.com/00007691-201608000-00001

39. Amstutz U, Shear NH, Rieder MJ, Hwang S, Fung V, Nakamura H, et al. Recommendations for HLA-B15:02 and HLA-A31:01 genetic testing to reduce the risk of carbamazepine-induced hypersensitivity reactions [Internet]. Vol. 55, Epilepsia. Blackwell Publishing Inc.; 2014 [cited 2020 Jul 6]. p. 496–506. Available from: http://doi.wiley.com/10.1111/epi.12564

40. Quaranta S, Thomas F. Pharmacogenetics of anti-cancer drugs: State of the art and implementation – recommendations of the French National Network of Pharmacogenetics. Therapie [Internet]. 2017 Apr 1 [cited 2020 Jul 9];72(2):205–15. Available from: https://linkinghub.elsevier.com/retrieve/pii/S0040595717300082

41. Lamoureux F, Duflot T. Pharmacogenetics in cardiovascular diseases: State of the art and implementation-recommendations of the French National Network of Pharmacogenetics (RNPGx). Therapie [Internet]. 2017 Apr 1 [cited 2020 Jul 9];72(2):257–67. Available from: https://linkinghub.elsevier.com/retrieve/pii/S0040595717300100

42. Woillard JB, Chouchana L, Picard N, Loriot MA. Pharmacogenetics of immunosuppressants: State of the art and clinical implementation – recommendations from the French National Network of Pharmacogenetics (RNPGx). Therapie [Internet]. 2017 Apr 1 [cited 2020 Jul 9];72(2):285–99. Available from: https://linkinghub.elsevier.com/retrieve/pii/S0040595717300094

43. PharmGKB. PharmGKB website [Internet]. Prescribing info. 2020 [cited 2020 Jul 15]. Available from: https://www.pharmgkb.org/

44. Quaranta S, Dupouey J, Colle R, Verstuyft C. Pharmacogenetics of antidepressant drugs: State of the art and clinical implementation – recommendations from the French National Network of Pharmacogenetics. Therapies [Internet]. 2017 Apr [cited 2020 Jul 9];72(2):311–8. Available from: https://linkinghub.elsevier.com/retrieve/pii/S0040595717300112

45. Swen JJ, Nijenhuis M, De Boer A, Grandia L, Maitland-Van Der Zee AH, Mulder H, et al. Pharmacogenetics: From bench to byte an update of guidelines. Clin Pharmacol Ther [Internet]. 2011;89(5):662–73. Available from: http://dx.doi.org/10.1038/clpt.2011.34/nature06264

46. Swen JJ, Wilting I, Goede A De, Grandia L, Mulder H, Touw DJ, et al. Pharmacogenetics: From bench to byte. Clin Pharmacol Ther. 2008;83(5):781–7.

47. Caudle KE, Dunnenberger HM, Freimuth RR, Peterson JF, Burlison JD, Whirl-Carrillo M, et al. Standardizing terms for clinical pharmacogenetic test results: Consensus terms from the Clinical Pharmacogenetics Implementation Consortium (CPIC). Genet Med. 2017;19(2):215–23.

48. Picard N, Boyer JC, Etienne-Grimaldi MC, Barin-Le Guellec C, Thomas F, Loriot MA. Pharmacogenetics-based personalized therapy: Levels of evidence and recommendations from the French Network of Pharmacogenetics (RNPGx). Therapie [Internet]. 2017;72(2):185–92. Available from: http://dx.doi.org/10.1016/j.therap.2016.09.014
