# Supplementary material for: Pharmacogenetics Guidelines: Overview and Comparison of the DPWG, CPIC, CPNDS, and RNPGx Guidelines
Source: Front Pharmacol. 2021 Jan 25;11:595219. doi: 10.3389/fphar.2020.595219 (PMC7868558; doi:10.3389/fphar.2020.595219)
Supplement: Supplementary file 2 [file table2.docx]

*Table 2:  aspects of methodology for guideline development.*

|  | DPWG  The Dutch Pharmacogenetics Working Group | CPIC  The Clinical Pharmacogenetics Implementation Consortium | CPNDS  The Canadian Pharmacogenomics Network for Drug Safety | RNPGx  the French National Network (Réseau) of Pharmacogenetics |
| --- | --- | --- | --- | --- |
| Country | The Netherlands | USA | Canada | Across France as well as other French-speaking nations (i.e., Belgium and more recently Switzerland and Canada) |
| Level of evidence | 0–4  The highest grade - 4: Published controlled studies of good quality relating to phenotyped and/or genotyped patients or healthy volunteers, and having relevant pharmacokinetic or clinical endpoints.  3: Published controlled studies of moderate qualityb relating to phenotyped and/or genotyped patients or healthy volunteers, and having relevant pharmacokinetic or clinical endpoints.  2: Published case reports, well documented, and having relevant pharmacokinetic or clinical endpoints. Well documented case series.  1: Published incomplete case reports Product information.  The lowest grade - 0: Data on file. | High (level 1A, 1B): evidence includes consistent results from well-designed, well-conducted studies.  Moderate (level 2A, 2B): Evidence is sufficient to determine effects, but the strength of the evidence is limited by the number, quality or consistency of the individual studies, generalizability to routine practice, or indirect nature of the evidence.  Weak (level 3): Evidence is insufficient to assess the effects on health outcomes because of limited number or power of studies, important flaws in their design or conduct, gaps in the chain of evidence, or lack of information.  Preliminary (level 4): Annotation based on a case report, non-significant study or in vitro, molecular or functional assay evidence only. (1) | the Appraisal of Guidelines Research and Evaluation Enterprise (AGREE):  + to ++++  The lowest grade +: meaning “Inconsistent or insufficient quantity/quality, discouraging” (further described as follows: “No conclusions can be drawn or conclusions are likely to change based on future studies, and current evidence is discouraging”).  The grade ++: meaning “Inconsistent or insufficient quantity/quality” (further described as follows: “No general conclusions can be drawn or conclusions are likely to change based on further research, but current evidence is encouraging”).  The grade +++: meaning “Consistent, but limited quantity or quality of studies, limiting generalizability” (further described as follows: Evidence allows general conclusions, but with reduced confidence; further research is likely to have an important impact on confidence in conclusions”).  The highest grade ++++: meaning “Consistent, generalizable” (further described as follows: “Strong general conclusions can be drawn that are unlikely to change based on further research”) (2). | Functionality of genetic variant  Demonstrated functionality: Direct functional impact on the expression or activity of a ‘‘pharmacogene’’ product demonstrated in vitro, with ex vivo data in humans corroborating this functional impact Indirect functional impact on the expression or activity of a ‘‘pharmacogene’’ product (existence of a linkage disequilibrium within a haplotype containing the deleterious genetic variation), with ex vivo data in humans corroborating this functional impact.  Probable functionality: direct (demonstrated in vitro) or indirect (by linkage disequilibrium) functional impact on the expression or activity of a ‘‘pharmacogene’’ produce, without ex vivo data in humans, or a functional impact that has not been the object of an in vitro demonstration.  Potential functionality: in silico predicted functional impact (change in protein sequence, localization in a functional domain, modeling) (3). |
| Clinical relevance or impact/recommendation categories | AA#–F  AA#: positive clinical effect.  AA: Clinical effect (NS). Kinetic effect (NS)  A: Minor clinical effect (S): QTc prolongation (<450 ms ♀, <470 ms ♂), INR increase <4.5 Kinetic effect (S).  B: Clinical effect (S): short-lived discomfort (<48 h) without permanent injury, for example, reduced decrease in resting heart rate, reduction in exercise tachycardia, diminished pain relief from oxycodone and ADE resulting from increased bioavailability of atomoxetine (decreased appetite, insomnia, sleep disturbance, etc.).  C: Clinical effect (S): long-standing discomfort (48–168 h) without permanent injury, for example, increase risk of failure of therapy with tricyclic antidepressants or atypical antipsychotic drugs: extrapyramidal side effects, parkinsonism: ADE resulting from increased bioavailability of tricyclic antidepressants, metoprolol, propafenone (central effects, e.g., dizziness).  D: Clinical effect (S): long-standing effect (>168 h), permanent symptom or invalidating injury, for example, failure of prophylaxis of atrial fibrillation; deep vein thrombosis.  E: Clinical effect (S): Increased risk of failure of lifesaving therapy; expected bone marrow depression.  F: Clinical effect (S): death; arrhythmia; unexpected bone marrow depression. (4–6) | Strong: “The evidence is high quality and the desirable effects clearly outweigh the undesirable effects”.  Moderate:“There is a close or uncertain balance as to whether the evidence is high quality and the desirable clearly outweigh the undesirable effects”.  Optional: (CPIC, 2020). | A – strong: “Based on strong scientific evidence; benefits clearly outweigh risks”.  B – moderate: “Based on reduced confidence scientific evidence and expert opinion; benefits likely to outweigh risks”.  C – optional: “Based mainly on expert opinion, for use with evidence development in a research context” (2). |  |
| Overall | The overall score genotype-drug combination is combination of the highest level of evidence and the highest level of relevance assigned to any of the articles included in the assessment. | Recommendations of the categories strong and moderate are being developed into guidelines and published. |  | Demonstrated or probable functionality are considered for a recommendation of testing. |
| Levels of recommendation for pharmacogenetic testing by genotyping | The clinical implementation score:  Potentially beneficial: PGx testing for this gene-drug pair is potentially beneficial. Genotyping can be considered on an individual patient basis. If, however, the genotype is available, the DPWG recommends adhering to the gene-drug guideline.  Beneficial: PGx testing for this gene-drug pair is beneficial. It is advised to genotype the patient before (or directly after) drug therapy has been initiated to guide drug and dose selection.  Essential: PGx testing for this gene-drug pair is essential for drug safety or efficacy. Genotyping must be performed before drug therapy has been initiated to guide drug and dose selection.  See also Table 2 (6).  The criteria for this score is based on:  (i) clinical effect,  (ii) level of evidence,  (iii) number needed to genotype, and  (iv) pharmacogenetics information in SmPCs. See Table 2 (6). |  | A – strong: “Based on strong scientific evidence; benefits clearly outweigh risks”. Level A is expected to be chosen by majority of healthcare proffessionals and patients.  B – moderate: “Based on reduced confidence scientific evidence and expert opinion; benefits likely to outweigh risks”. Level B is needs individualized informed decision making.  C – optional: “Based mainly on expert opinion, for use with evidence development in a research context”  (2,7,8). | Essential test: demonstrated or probable functionality Demonstrated impact on a major clinical phenotype [response (efficacy, resistance)/toxicity] for therapeutic management; difficult or impossible to predict with a non-genetic approach; having led to expert agreement in favor of systematic testing.  Advisable test: demonstrated functionality Demonstrated impact on an intermediary non-clinical phenotype but that is important to predict drug exposure (e.g. pharmacokinetics) for therapeutic management, having led to expert agreement in favor of testing Demonstrated functionality Demonstrated impact on a major clinical phenotype in therapeutic management but predictable by a non-genetic approach (phenotyping), having led to expert agreement in favor of testing as a complement to phenotyping or when phenotyping is not possible as a first-intention approach.  Possibly helpful test: demonstrated or probable functionality Probable impact that remains to be demonstrated on a clinical phenotype or on an intermediary (non-clinical) phenotype having led to expert consensus in favor of testing, case-by-case, depending on the clinical context (unusual response to a drug, specific disease). |

ADE, adverse drug event; INR, international normalized ratio; NS, not statistically significant difference; S, statistically significant difference.

**References**

1. CPIC. Clinical Pharmacogenetics Implemetation Consortium (CPIC) [Internet]. Guidelines. 2020 [cited 2020 Jul 11]. Available from: https://cpicpgx.org/guidelines

2. Amstutz U, Shear NH, Rieder MJ, Hwang S, Fung V, Nakamura H, et al. Recommendations for HLA-B15:02 and HLA-A31:01 genetic testing to reduce the risk of carbamazepine-induced hypersensitivity reactions [Internet]. Vol. 55, Epilepsia. Blackwell Publishing Inc.; 2014 [cited 2020 Jul 6]. p. 496–506. Available from: http://doi.wiley.com/10.1111/epi.12564

3. Picard N, Boyer JC, Etienne-Grimaldi MC, Barin-Le Guellec C, Thomas F, Loriot MA. Pharmacogenetics-based personalized therapy: Levels of evidence and recommendations from the French Network of Pharmacogenetics (RNPGx). Therapie [Internet]. 2017;72(2):185–92. Available from: http://dx.doi.org/10.1016/j.therap.2016.09.014

4. Swen J, Nijenhuis M, De Boer A, Grandia L, Maitland-van der Zee A, Mulder H, et al. Pharmacogenetics: From Bench to Byte— An Update of Guidelines. Clin Pharmacol Ther |. 2011;89(5):662–73.

5. Swen JJ, Wilting I, Goede A De, Grandia L, Mulder H, Touw DJ, et al. Pharmacogenetics: From bench to byte. Clin Pharmacol Ther. 2008;83(5):781–7.

6. Swen JJ, Nijenhuis M, van Rhenen M, de Boer-Veger NJ, Buunk AM, Houwink EJF, et al. Pharmacogenetic Information in Clinical Guidelines: The European Perspective. Clin Pharmacol Ther. 2018;103(5):795–801.

7. Tanoshima R, Khan A, Biala AK, Trueman JN, Drögemöller BI, Wright GEB, et al. Analyses of Adverse Drug Reactions–Nationwide Active Surveillance Network: Canadian Pharmacogenomics Network for Drug Safety Database. J Clin Pharmacol. 2019;59(3):356–63.

8. Ross CJD, Visscher H, Sistonen J, Brunham LR, Pussegoda K, Loo TT, et al. The Canadian Pharmacogenomics Network for Drug Safety: a model for safety pharmacology. Thyroid. 2010;20(7):681–7.
