# Supplementary material for: Pharmacogenetics Guidelines: Overview and Comparison of the DPWG, CPIC, CPNDS, and RNPGx Guidelines
Source: Front Pharmacol. 2021 Jan 25;11:595219. doi: 10.3389/fphar.2020.595219 (PMC7868558; doi:10.3389/fphar.2020.595219)
Supplement: Supplementary file 3 [file table3.docx]

Table 3A:  The criteria of the DPWG Clinical Implication Score (Swen et al., 2008, 2018; Deneer V.H.M., 2013).

| **Clinical Implication Score Criteria** | **Possible Score** | **Given**  **Score** |
| --- | --- | --- |
| **Clinical effect associated with gene-drug interaction (drug- or diminished efficacy-induced)**  •       CTCAE Grade 3 or 4 (clinical effect score D or E)  •  CTCAE Grade 5 (clinical effect score F) | +  ++ |  |
| **Level of evidence supporting the associated clinical effect grade ≥ 3**  •       One study with level of evidence score ≥ 3  •       Two studies with level of evidence score ≥ 3  •       Three or more studies with level of evidence score ≥ 3 | +  ++ +++ |  |
| **Number needed to genotype (NNG) in the Dutch population to prevent one clinical effect grade ≥ 3**  • 100 < NNG ≤ 1000  • 10 < NNG ≤ 100  • NNG ≤ 10 | +  ++  +++ |  |
| **PGx information in the Summary of Product Characteristics (SmPC)**  • At least one genotype/phenotype mentioned  OR  • Recommendation to genotype  OR  • At least one genotype/phenotype mentioned as a contra-indication in the corresponding section | +  ++  ++ |  |
| **Total Score:** |  |  |
| **Corresponding Clinical Implication Score:** | |  |

The clinical implication score is only calculated for gene-drug interactions for which an adjustment of therapy is advised.

For each row one score is assigned, for example the score for row one can be 0. The scores for each individual rows are added leading to a total score ranging from 0+ to 10+.The description of the “clinical effect score” and “level of evidence score” is described earlier by of Swen et al. and Deneer et al. (Swen et al., 2008, 2018; Deneer V.H.M., 2013).

CTCAE: Common Terminology Criteria for Adverse Events. PGx: pharmacogenomics. SPC, Summary of Product Characteristics.

Table 3B: Definitions of the categories of the DPWG Clinical Implication Score (Swen et al., 2008, 2018; Deneer V.H.M., 2013).

| **Potentially Beneficial** | PGx testing for this gene-drug pair is potentially beneficial. Genotyping can be considered on an individual patient basis. If, however, the genotype is available, the DPWG recommends adhering to the gene-drug guideline | 0-2 +*  (= + to ++) |
| --- | --- | --- |
| **Beneficial** | PGx testing for this gene-drug pair is beneficial. It is advised to genotype the patient before (or directly after) drug therapy has been initiated to guide drug and dose selection | 3-5 +* (=+++ to +++++) |
| **Essential** | PGx testing for this gene-drug pair is essential for drug safety or efficacy. Genotyping must be performed before drug therapy has been initiated to guide drug and dose selection | 6-10 +  (=++++++ to ++++++++++) |

* this is the sum of the number of “+”.

PGx: pharmacogenomics.

**References**

Deneer V.H.M., van S. R. H. N. (2013) (2013). Evidence Based Drug Dosing and Pharmacotherapeutic Recommendations per Genotype. *Methods Mol. Biol. (Methods Protoc.* 1015, 71–85. doi:10.1007/978-1-62703-435-7.

Swen, J. J., Nijenhuis, M., van Rhenen, M., de Boer-Veger, N. J., Buunk, A. M., Houwink, E. J. F., et al. (2018). Pharmacogenetic Information in Clinical Guidelines: The European Perspective. *Clin. Pharmacol. Ther.* 103, 795–801. doi:10.1002/cpt.1049.

Swen, J. J., Wilting, I., Goede, A. De, Grandia, L., Mulder, H., Touw, D. J., et al. (2008). Pharmacogenetics: From bench to byte. *Clin. Pharmacol. Ther.* 83, 781–787. doi:10.1038/sj.clpt.6100507.
