# Supplementary material for: Pharmacogenetics Guidelines: Overview and Comparison of the DPWG, CPIC, CPNDS, and RNPGx Guidelines
Source: Front Pharmacol. 2021 Jan 25;11:595219. doi: 10.3389/fphar.2020.595219 (PMC7868558; doi:10.3389/fphar.2020.595219)
Supplement: Supplementary file 4 [file table4.docx]

Table 4: Main discordances in pharmacotherapeutic recommendations of the DPWG, CPIC, CPNDS, and RNPGx.

| **Genotpe/phenotype** | **Drug** | **Recommendation** | **Ref** |
| --- | --- | --- | --- |
| CYP2C19 | Citalopram and escitalopram | **DPWG**:  - UM citalopram: no action is required.  - UM escitalopram: avoid.  Do not exceed the daily doses as shown in Table 1 for IM and PM:  - IM: do not exceed 75% of the standard maximum dose.  - PM: do not exceed 50% of the standard maximum dose.  **CPIC:**  - UM: consider an alternative.  - IM: initiate therapy with recommended dose.  - PM: consider a 50% reduction of recommended starting dose and titrate to response or select alternative drug not predominantly metabolized by CYP2C19. | (1,2) |
| CYP2C19 | Clopidogrel | **DPWG**:  - IM: choose an alternative for clopidogrel for the indications of percutaneous coronary intervention (PCI), stroke, or Transient Ischemic Attack (TIA), or double the daily dosage to 150 mg.  - No action is needed in case of other indications.  - PM: consider an alternative in case of percutaneous coronary intervention (PCI), stroke, or TIA.  **CPIC**: choose an alternative in CYP2C19 IM and PM patients for patients with an acute coronary syndrome undergoing PCI.  **RNPGx**: choose an alternative in CYP2C19 IM and PM patients. | (1,3,4) |
| CYP2C19 | Sertraline | **DPWG**:  - UM and IM: no action is needed.  - PM: do not give doses exceeding 75 mg/day.  **CPIC:**  - UM: initiate therapy with recommended starting dose. In case of no response, consider alternative drug.  - IM: initiate therapy with recommended starting dose.  - PM: consider a 50% reduction of recommended starting dose and titrate to response or select alternative drug. | (1,2) |
| CYP2C19-CYP2D6 | Tricyclic antidepressants (TCA) | **DPWG**:  - CYP2D6 UM: increase the dose, see Table 1.  - CYP2D6 IM and PM: decrease the dose, see Table 1.  - CYP2C19 UM: no additional action is required for patients starting amitriptyline.  **CPIC**:  - CYP2C19 UM and PM: avoid high dose.  - CYP2D6 UM and PM: avoid high dose.  - CYP2D6 IM: reduce the dose (−25% of the recommended dose).  **RNPGx**:  - CYP2C19 UM and PM: avoid high dose.  - CYP2D6 UM and PM: avoid high dose.  - CYP2D6 IM: reduce the dose (−50% of the recommended dose). | (1,5–7) |
| CYP2C19 | Voriconazole | **DPWG**:  - UM: use an initial dose that is 1.5x higher and monitor the plasma concentration.  - IM: monitor the plasma concentration.  - PM: use 50% of the standard dose and monitor the plasma concentration.  **CPIC – adults:**  - UM and RM: choose an alternative.  - IM: initiate therapy with recommended standard of care dosing.  - PM: choose an alternative. In the event that voriconazole is considered to be the most appropriate agent, voriconazole should be administered at a preferably lower than standard dosage with careful therapeutic drug monitoring (TDM).  **CPIC – pediatrics:**  - UM: choose an alternative.  - RM: initiate therapy with recommended and use TDM to titrate dose.  - IM: initiate therapy with recommended standard of care dosing.  - PM: choose an alternative. In the event that voriconazole is considered to be the most appropriate agent, voriconazole should be administered at a preferably lower than standard dosage with careful TDM. | (8) |
| CYP2C9-VKORC1 | Warfarin | **DPWG**: use the EU-PACT algorithm to calculate the dose.  **CPIC**: use [www.WarfarinDosing.org](http://www.WarfarinDosing.org) to calculate the dose. Use the Gage or the IWPC algorithms or both.  **CPNDS**: use [www.WarfarinDosing.org](http://www.WarfarinDosing.org) to calculate the dose. | (4,9,10) |
| CYP2D6 | Codeine | **DPWG:**  Doses higher than 20 mg every 6 hours for adults and 10 mg every 6 hours for children aged 12 years or older and/or additional risk factors, such as co-medication with CYP3A4 inhibitors and/or reduced kidney function:  - Codeine is contra-indicated, if possible, select an alternative  - For pain: do not select tramadol, see Table 1.  - For cough: noscapine is not metabolized by CYP2D6.  **CPIC:**  - UM: avoid codeine use due to potential for toxicity.  - IM: use label-recommended age or weight-specific dosing. If no response, consider alternative analgesics such as morphine or a non-opioid.  - PM: avoid codeine use due to lack of efficacy.  **CPNDS:**  - UM: avoid codeine for pain relief and receive alternative analgesics that do not have potent CYP2D6 metabolites.  - PM: these patients should not receive codeine for pain relief. | (1,5,11–13) |
| CYP2D6 | Tamoxifen | **DPWG**:  - UM: no action is needed.  - IM:  1. Select an alternative or measure the endoxifen concentration.  2. If tamoxifen is selected: avoid co-medication with CYP2D6 inhibitors.  - PM: select an alternative or increase the dose to 40 mg/day and monitor the endoxifen concentration.  **CPIC**:  - UM: avoid moderate and strong CYP2D6 inhibitors. Initiate therapy with recommended standard of care dosing.  - IM (AS 1.0 and 0.5): consider hormonal therapy, see Table 1. If aromatase inhibitor use is contraindicated, consideration should be given to use a higher but FDA approved tamoxifen dose (40 mg/day).  - PM: recommend alternative hormonal therapy, see Table 1. Note, higher dose tamoxifen (40 mg/day) increases but does not normalize endoxifen concentrations and can be considered if there are contraindications to aromatase inhibitor therapy.  **CPNDS:**  IM/PM: tamoxifen 40mg/day. | (1,5,14,15) |
| DPYD AS: 0 | Fluorouracil or capecitabine | **DPWG**: avoid. If not possible determine residual DPD activity in mononuclear cells from peripheral blood and adjust the dose accordingly.  **CPIC**: Avoid.  **RNPGx**: contraindicated. | (1,7,16–18). |
| SLCO1B1 | Statins | **DPWG**:  - In patients with SLCO1B1 521 CC and TC, and additional risk factors for statin induced myopathy - choose an alternative for simvastatin and atrovastatin.  - In SLCO1B1 TC patients: simvastatin doses exceeding 40 mg/day should be avoided, if selecting an alternative is not an option.  **CPIC**:  - Reduce the normal dose for patients with intermediate or low function of the transporter or choose an alternative (e.g. rosuvastatin or pravastatin).  - The CPIC has no recommendations for atorvastatin.  **RNPGx**:  - SLCO1B1 *5/*5 and *1/*5: avoid high doses of statins and the concomitant use of OATP1B1 inhibitors and/or CYP3A4 inhibitors (such as amiodarone, verapamil, and diltiazem).  - Lower the simvastatin dose to 20 mg per day or select another statin. | (1,4,12,19) |
| UGT1A1 PM | Irinotecan | **DPWG**: start with 70% of standard dose.  **RNPGx:**  - Start with 70-75% of standard dose in case of 180-230 mg/m^2^ spaced by 2 – 3 weeks.  - Contraindicated in case of 240 mg/m^2^ or higher in spaced by 2 – 3 weeks. | (1,7) |

TDM: Therapeutic Drug Monitoring. UM: ultrarapid metabolizer. RM: rapid metabolizer. NM: normal metabolizer. IM: intermediate metabolizer. PM: poor metabolizer.

**References:**

1. The Dutch Pharmacogenomic Working Group (DPWG). Phamacogenomic recommendations, farmacogenetica-update [Internet]. 2020 [cited 2020 Jul 12]. Available from: www.knmp.nl/

2. Hicks J, Bishop J, Sangkuhl K, Ji Y, Leckband S, Leeder J, et al. Clinical Pharmacogenetics Implementation Consortium (CPIC) Guideline for CYP2D6 and CYP2C19 Genotypes and Dosing of Selective Serotonin Reuptake Inhibitors. [cited 2020 Jul 10]; Available from: www.pharmgkb.org,

3. Scott S, Sangkuhl K, Stein C, Hulot J, Mega J, Roden D, et al. Clinical Pharmacogenetics Implementation Consortium Guidelines for CYP2C19 Genotype and Clopidogrel Therapy: 2013 Update. 2013 [cited 2020 Jul 10]; Available from: www.pharmgkb.

4. Lamoureux F, Duflot T. Pharmacogenetics in cardiovascular diseases: State of the art and implementation-recommendations of the French National Network of Pharmacogenetics (RNPGx). Therapie [Internet]. 2017 Apr 1 [cited 2020 Jul 9];72(2):257–67. Available from: https://linkinghub.elsevier.com/retrieve/pii/S0040595717300100

5. Swen J, Nijenhuis M, De Boer A, Grandia L, Maitland-van der Zee A, Mulder H, et al. Pharmacogenetics: From Bench to Byte— An Update of Guidelines. Clin Pharmacol Ther |. 2011;89(5):662–73.

6. Hicks JK, Sangkuhl K, Swen JJ, Ellingrod VL, M€ Uller DJ, Shimoda K, et al. Clinical Pharmacogenetics Implementation Consortium Guideline (CPIC) for CYP2D6 and CYP2C19 Genotypes and Dosing of Tricyclic Antidepressants: 2016 Update. [cited 2020 Jul 10]; Available from: www.cpt-journal.com

7. Quaranta S, Thomas F. Pharmacogenetics of anti-cancer drugs: State of the art and implementation – recommendations of the French National Network of Pharmacogenetics. Therapie [Internet]. 2017 Apr 1 [cited 2020 Jul 9];72(2):205–15. Available from: https://linkinghub.elsevier.com/retrieve/pii/S0040595717300082

8. Moriyama B, Obeng AO, Barbarino J, Penzak SR, Henning SA, Scott SA, et al. Clinical Pharmacogenetics Implementation Consortium (CPIC) Guidelines for CYP2C19 and Voriconazole Therapy. [cited 2020 Jul 10]; Available from: http://www.pharmgkb.organdhttps//cpicpgx.org/guidelines/.

9. The Dutch Pharmacogenomic Working Group (DPWG). VKORC1: warfarin [Internet]. 2016 [cited 2020 Jul 11]. p. 16. Available from: https://www.g-standaard.nl/risicoanalyse/B0006235.PDF

10. Shaw K, Amstutz U, Kim RB, Lesko LJ, Turgeon J, Michaud V, et al. Clinical Practice Recommendations on Genetic Testing of CYP2C9 and VKORC1 Variants in Warfarin Therapy [Internet]. Vol. 37, Therapeutic Drug Monitoring. Lippincott Williams and Wilkins; 2015 [cited 2020 Jul 6]. p. 428–36. Available from: http://journals.lww.com/00007691-201508000-00002

11. Parvaz Madadi UAMRSIVFSHJTVMGKBCCCCRG. Clinical Practice Guideline: CYP2D6 Genotyping for Safe and Efficacious Codeine Therapy - PubMed. J Popul Ther Clin Pharmacol [Internet]. 2013 Nov 6 [cited 2020 Jul 6];20(3):369–96. Available from: https://pubmed.ncbi.nlm.nih.gov/24214521/

12. Picard N, Boyer JC, Etienne-Grimaldi MC, Barin-Le Guellec C, Thomas F, Loriot MA. Pharmacogenetics-based personalized therapy: Levels of evidence and recommendations from the French Network of Pharmacogenetics (RNPGx). Therapie [Internet]. 2017;72(2):185–92. Available from: http://dx.doi.org/10.1016/j.therap.2016.09.014

13. Crews K, Gaedigk A, Dunnenberger H, Leeder J, Klein T, Caudle K, et al. Clinical Pharmacogenetics Implementation Consortium Guidelines for Cytochrome P450 2D6 Genotype and Codeine Therapy: 2014 Update. 2014 [cited 2020 Jul 10]; Available from: www.cypalleles.ki.se/cyp2d6.htm,

14. Goetz MP, Sangkuhl K, Guchelaar H-J, Schwab M, Province M, Whirl-Carrillo M, et al. Clinical Pharmacogenetics Implementation Consortium (CPIC) Guideline for CYP2D6 and Tamoxifen Therapy. [cited 2020 Jul 10]; Available from: www.cpicpgx.org/guidelines/.

15. Drögemöller BI, Wright GEB, Shih J, Monzon JG, Gelmon KA, Ross CJD, et al. CYP2D6 as a treatment decision aid for ER-positive non-metastatic breast cancer patients: a systematic review with accompanying clinical practice guidelines [Internet]. Vol. 173, Breast Cancer Research and Treatment. Springer New York LLC; 2019 [cited 2020 Jul 6]. p. 521–32. Available from: http://link.springer.com/10.1007/s10549-018-5027-0

16. Lunenburg CATC, van der Wouden CH, Nijenhuis M, Crommentuijn-van Rhenen MH, de Boer-Veger NJ, Buunk AM, et al. Dutch Pharmacogenetics Working Group (DPWG) guideline for the gene–drug interaction of DPYD and fluoropyrimidines. Eur J Hum Genet [Internet]. 2020;28(4):508–17. Available from: http://dx.doi.org/10.1038/s41431-019-0540-0

17. CPIC. Clinical Pharmacogenetics Implemetation Consortium (CPIC) [Internet]. Guidelines. 2020 [cited 2020 Jul 11]. Available from: https://cpicpgx.org/guidelines

18. Caudle KE, Sangkuhl K, Whirl-Carrillo M, Swen JJ, Haidar CE, Klein TE, et al. Standardizing CYP2D6 Genotype to Phenotype Translation: Consensus Recommendations from the Clinical Pharmacogenetics Implementation Consortium and Dutch Pharmacogenetics Working Group. Clin Transl Sci. 2020;13(1):116–24.

19. Ramsey L, Johnson S, Caudle K, Haidar C, Voora D, Wilke R, et al. The Clinical Pharmacogenetics Implementation Consortium Guideline for SLCO1B1 and Simvastatin-Induced Myopathy: 2014 Update. 2014 [cited 2020 Jul 10]; Available from: http://www.pharmgkb.org.
