# Supplementary material for: Pharmacogenetics Guidelines: Overview and Comparison of the DPWG, CPIC, CPNDS, and RNPGx Guidelines
Source: Front Pharmacol. 2021 Jan 25;11:595219. doi: 10.3389/fphar.2020.595219 (PMC7868558; doi:10.3389/fphar.2020.595219)
Supplement: Supplementary file 5 [file table5.docx]

Table 5: Gene and drug combinations with the highest level of genetic testing recommendation of the DPWG, CPNDS, and RNPGx.

|  | **Gene** | **Drug** | **Indication** | **Ref** |
| --- | --- | --- | --- | --- |
| DPWG | HLA-B*5701 | Abacavir |  | (1) |
|  | NUDT15 | Azathioprine, mercaptopurine, and thioguanine |  | (1) |
|  | DPYD | Capecitabine, fluorouracil (systemic), and tegafur |  | (1) |
|  | CYP2C19 | Clopidogrel | PCI, stroke or TIA | (1) |
|  | CYP2D6 | Codeine | Planned doses > 20 mg every 6 hours for adults and > 10 mg every 6 hours for children aged ≥ 12 years or additional risk factors, such as comedication with CYP3A4-inhibitors and/or reduced kidney function | (1,2) |
|  | UGT1A1 | Irinotecan |  | (1) |
|  |  |  |  |  |
| CPNDS | CYP2D6 | Codeine |  | (3) |
|  | TPMT | Cisplatin | TPMT variants (*3A, *3B, and*3C) in all patients, and functionally inactive TPMT *2 variant in children | (4) |
|  |  |  |  |  |
| RNPGx | UGT1A1 | Irinotecan | 240 mg/m^2^ or higher spaced by 2 – 3 week intervals | (5) |
|  | CYP2C19 | Clopidogrel |  | (6) |
|  | DPYD | Capecitabine and Fluorouracil |  | (5) |

PCI: percutaneous coronary intervention. TIA: Transient Ischemic Attack.

**References**

1. The Dutch Pharmacogenomic Working Group (DPWG). Phamacogenomic recommendations, farmacogenetica-update [Internet]. 2020 [cited 2020 Jul 12]. Available from: www.knmp.nl/

2. Swen J, Nijenhuis M, De Boer A, Grandia L, Maitland-van der Zee A, Mulder H, et al. Pharmacogenetics: From Bench to Byte— An Update of Guidelines. Clin Pharmacol Ther |. 2011;89(5):662–73.

3. Parvaz Madadi UAMRSIVFSHJTVMGKBCCCCRG. Clinical Practice Guideline: CYP2D6 Genotyping for Safe and Efficacious Codeine Therapy - PubMed. J Popul Ther Clin Pharmacol [Internet]. 2013 Nov 6 [cited 2020 Jul 6];20(3):369–96. Available from: https://pubmed.ncbi.nlm.nih.gov/24214521/

4. Lee JW, Pussegoda K, Rassekh SR, Monzon JG, Liu G, Hwang S, et al. Clinical Practice Recommendations for the Management and Prevention of Cisplatin-Induced Hearing Loss Using Pharmacogenetic Markers [Internet]. Vol. 38, Therapeutic Drug Monitoring. Lippincott Williams and Wilkins; 2016 [cited 2020 Jul 6]. p. 423–31. Available from: http://journals.lww.com/00007691-201608000-00001

5. Quaranta S, Thomas F. Pharmacogenetics of anti-cancer drugs: State of the art and implementation – recommendations of the French National Network of Pharmacogenetics. Therapie [Internet]. 2017 Apr 1 [cited 2020 Jul 9];72(2):205–15. Available from: https://linkinghub.elsevier.com/retrieve/pii/S0040595717300082

6. Lamoureux F, Duflot T. Pharmacogenetics in cardiovascular diseases: State of the art and implementation-recommendations of the French National Network of Pharmacogenetics (RNPGx). Therapie [Internet]. 2017 Apr 1 [cited 2020 Jul 9];72(2):257–67. Available from: https://linkinghub.elsevier.com/retrieve/pii/S0040595717300100
